# Syntheses and Reactions of Pyrroline, Piperidine Nitroxide Phosphonates

**DOI:** 10.3390/molecules25102430

**Published:** 2020-05-22

**Authors:** Mostafa Isbera, Balázs Bognár, József Jekő, Cecilia Sár, Kálmán Hideg, Tamás Kálai

**Affiliations:** 1 Institute of Organic and Medicinal Chemistry, Medical School, University of Pécs, Szigeti st. 12, 7624 Pécs, Hungary; mostafaisbera@gmail.com (M.I.); balazs.bognar@aok.pte.hu (B.B.); cecilia.sar@aok.pte.hu (C.S.); kalman.hideg@aok.pte.hu (K.H.); 2 Department of Chemistry, University of Nyíregyháza, Sóstói st. 31/B, 4440 Nyíregyháza, Hungary; jjozsi@gmail.com; 3 Szentágothai Research Centre, Ifjúság st. 20, H-7624 Pécs, Hungary

**Keywords:** antioxidant activity, Horner-Wadsworth-Emmons olefination, nitroxides, phosphonates

## Abstract

Organophosphorus compounds occupy a significant position among the plethora of organic compounds, but a limited number of paramagnetic phosphorus compounds have been reported, including paramagnetic phosphonates. This paper describes the syntheses and further transformations of pyrroline and piperidine nitroxide phosphonates by well-established methods, such as the Pudovik, Arbuzov and Horner-Wadsworth-Emmons (HWE) reactions. The reaction of paramagnetic α-bromoketone produced a vinylphosphonate in the Perkow reaction. Paramagnetic α-hydroxyphosphonates could be subjected to oxidation, elimination and substitution reactions to produce various paramagnetic phosphonates. The synthesized paramagnetic phosphonates proved to be useful synthetic building blocks for carbon-carbon bond-forming reactions in the Horner-Wadsworth-Emmons olefination reactions. The unsaturated compounds achieved could be transformed into various substituted pyrroline nitroxides, proxyl nitroxides and paramagnetic polyaromatics. The Trolox^®^ equivalent antioxidant capacity (TEAC) of new phosphonates was also screened, and tertiary α-hydroxyphosphonatate nitroxides exhibited remarkable antioxidant activity.

## 1. Introduction

**Figure 1:**
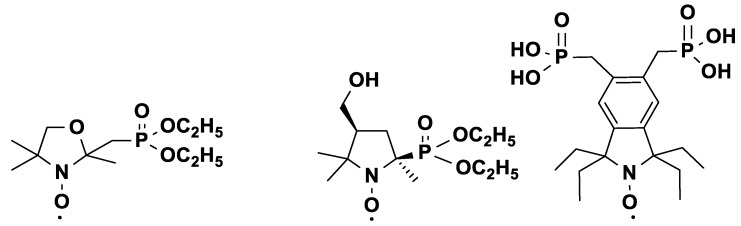
Previously reported paramagnetic phosphonates.

Functionalized phosphonates are fascinating organophosphorus compounds used in biology, pharmacology, agriculture and organic chemistry [1,2,3]. The main interest in preparation of these compounds originated from their application in the Horner-Wadsworth-Emmons (HWE) olefination reaction to produce various unsaturated compounds [4]. Despite the simplicity of the syntheses of phosphonates or α-hydroxyphosphonates or trialkylphosphates by the Arbuzov [5], Pudovik [6] or Perkow reactions [7], these reactions were applied limitedly to access paramagnetic phosphorus compounds, although many phosphorus containing nitroxides have been published [8,9,10,11]. Remarkable part of these materials are mainly 2-substituted β- or γ-phosphorylated five-membered nitroxides exhibiting a second notably large hyperfine splitting with the one-half spin nucleus of the phosphorus atom [12,13,14,15,16] (Figure 1). However, no further transformations of these paramagnetic phosphonates were reported beyond phosphonate hydrolysis [8] or transesterification [16]. In this paper, we report the syntheses of new pyrroline and piperidine nitroxide phosphonates starting from nitroxide halogenides, acetylenes, aldehydes and ketones. Our purpose was to evaluate the scope and limitations of the reactions of the newly synthesized paramagnetic phosphonates or α-hydroxyphosphonates as potential paramagnetic building blocks for spin labeling or construction of more complex paramagnetic scaffolds. Although paramagnetic phosphonium salts and their use in C=C bond-forming reactions have been published [17], considering the advantages of use of phosphonates [18] over phosphonium ylides (e.g., avoiding the formation of non-water-soluble triphenylphosphine oxide), paramagnetic phosphonates can be more appropriate building blocks for synthetic chemists working in this field.

## 2. Results and Discussion

### 2.1. Use of Arbusov Reaction

**Scheme 1:**
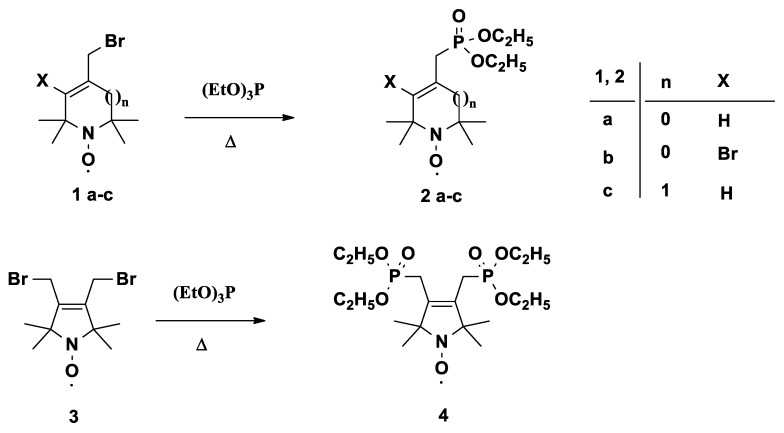
Synthesis of paramagnetic phosphonates by the Arbusov reaction.

Treatment of five- and six-membered allylic bromides **1a–c [19,20,21]** with triethyl phosphite at 120 °C with stirring in an open vessel resulted in the formation of phosphonates **2a–c** in 65–81% yield (monitored by thin layer chromatography). As expected in the case of compound **1b,** only the more reactive allylic bromide was converted to a phosphonate, and the vinyl bromine atom was not substituted. Under these conditions, we did not observe the reduction of nitroxide function. The same reaction could be performed with dibromo compound **3 [22]** to furnish bisphosphonate ester **4** (Scheme 1).

### 2.2. Use of HWE and Perkow Reaction

**Scheme 2:**
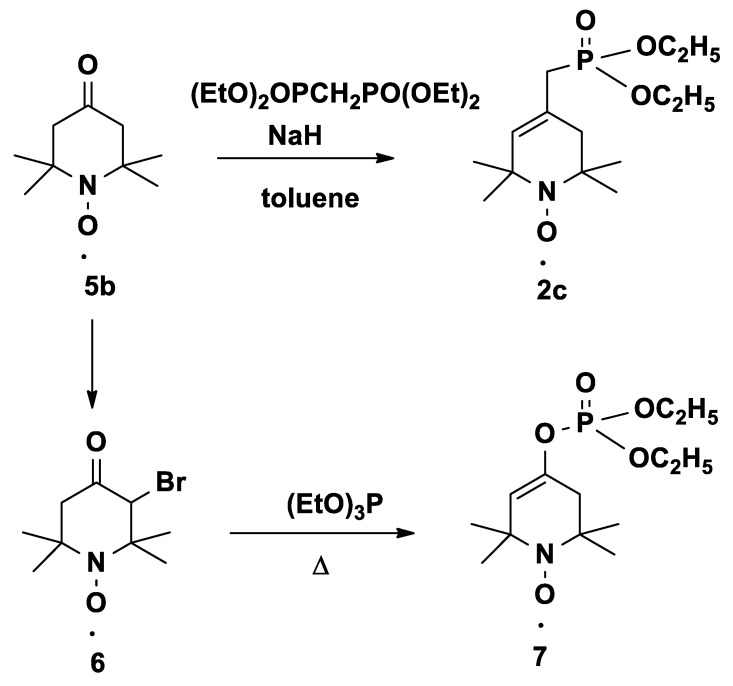
Synthesis of paramagnetic phosphonate (**2c**) by a HWE reaction and phosphate **7** by a Perkow reaction from 4-oxo-TEMPO (**5b**).

Because the synthesis of compound **1c** is a long multistep procedure from the readily available 4-oxo-TEMPO (1-oxyl-4-oxo-2,2,6,6-tetramethyplpiperidine radical) (**5b**) [21,23,24], we are pleased to report a simpler and more direct method that heats the sodium salt of tetraethyl methylenediphosphonate with compound **5b** in toluene at reflux temperature to produce compound **2c** in a HWE reaction, although at a slightly lower 58% yield. It is well known that upon heating, α-bromoketones with trialkylphosphites furnish dialkyl vinylphosphates [7]. The same reaction was observed with 3-bromo-1-oxyl-4-oxo-2,2,6,6-tetramethylpiperidine radical **6 [25]**, which upon heating with triethylphosphite at 120 °C furnished the paramagnetic vinylphosphate ester **7** in 34% yield (Scheme 2).

The formation of ketophosphonate in an Arbusov reaction can be excluded because the appearance of the vinyl proton at 5.43 ppm and the ^31^P-NMR shift at −6.22 ppm verify the formation of diethylvinyl phosphate **7**. The latter ^31^P-NMR data show good correlation with the reported values [26].

### 2.3. Pudovik Hydroxyphosphonate Synthesis and Transformations

**Scheme 3:**
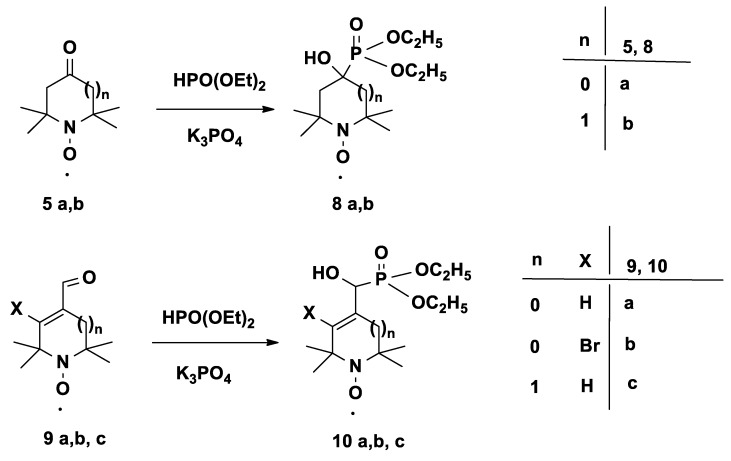
Synthesis of α-hydroxyphosphonates.

The above results drove our decision to study the reactions of paramagnetic aldehydes and ketones with diethyl phosphite to produce α-hydroxyphosphonates because these derivatives have biological importance, i.e., herbicidal, antibacterial, antifungal and antioxidant effects, to mention but a few [27,28,29]. To access paramagnetic α-hydroxyphosphonates among the possible reaction conditions [30,31] tested, we choose the methodology of Kulkarni et al. [32], e.g., solvent-free conditions in the presence of 0.05 eq. K_3_PO_4_. Therefore, treatment of ketones **5a [33]** or **5b [23]** or five- or six-membered nitroxide aldehydes **9a [34]**, **9b [20]**, or **9c [21]** with diethyl phosphite in the presence of 0.05 eq. K_3_PO_4_ offered the α-hydroxyphosphonates **8a** or **8b** or **10a** or **10b** or **10c**, respectively, in 78–92% yield (Scheme 3). The structure of these compounds is proven by the appearance of hydroxyl band of OH groups at ~3200 cm^−1^ compared with compounds **2a–c**. We attributed the conversion of α-hydroxyphosphonates **8a** or **8b** to the corresponding vinyl phosphonate by water elimination. By treatment of compound **8a** or **8b** with POCl_3_ in anhydr. pyridine [23] after 48 h at room temperature, **11** vinylphosphonate could be isolated from **8b** in 29% yield, but the expected five-membered vinylphosphonate was not formed under these conditions. The structure of vinylphosphonate **11** is proven by the split vinyl proton at 6.62 ppm with *J* = 21.5 Hz and the upfield shift of the ^31^P-NMR signal at 19.3 ppm compared with that of the compound **2c** ^31^P signal at 27.1 ppm (see Supplementary Materials). Further attempts to eliminate the water from compound **8a** with sulfuric acid [35] or FeCl_3_/silica gel microwave heating [36] did not produce the required vinyl phosphonate. Our efforts to substitute the tertiary alcohols **8a** or **8b** with various nucleophiles via mesylate did not succeed, similar to the same experiments with the secondary alcohols **10a–c**. For further possible transformations, we focused on compound **10a** conversions, which could be smoothly oxidized to α-ketophosphonate **12** with 3.0 eq. Dess–Martin periodinane (1,1,1-tris(acetyloxy)-1,1-dihydro-1,2-benziodoxol-3-(1*H*)-one) [37] in CH_2_Cl_2_ at room temperature. With the reaction of compound **10a** with DEAD (diethyl azodicarboxylate) and PPh_3_ in the presence of HN_3_ under Mitsonubu reaction conditions [38], we created paramagnetic α-azidophosphonate **13**.

**Scheme 4:**
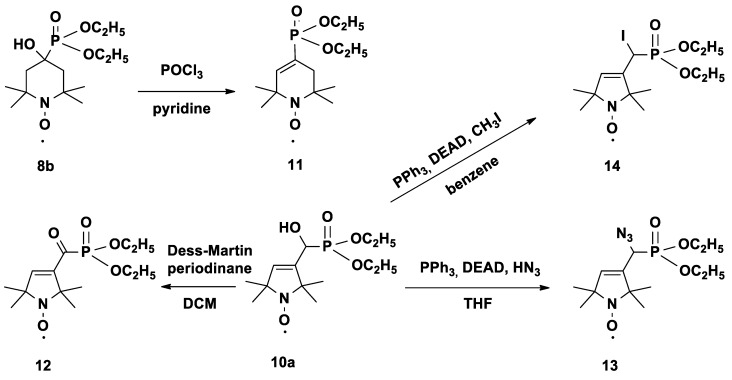
Further transformations of α-hydroxyphosphonates.

Under similar conditions and using methyl iodide as a source for the I^−^ nucleophile [39], we obtained iodo compound **14**, which was rather inert for attempts at further nucleophilic substitution conditions (Scheme 4). The limited success of these transformations is attributed to the sterically hindered allylic position, which is surrounded by a bulky phosphonate group and a densely substituted pyrroline nitroxide ring.

### 2.4. Phosphonate Synthesis with Lithiation

**Scheme 5:**
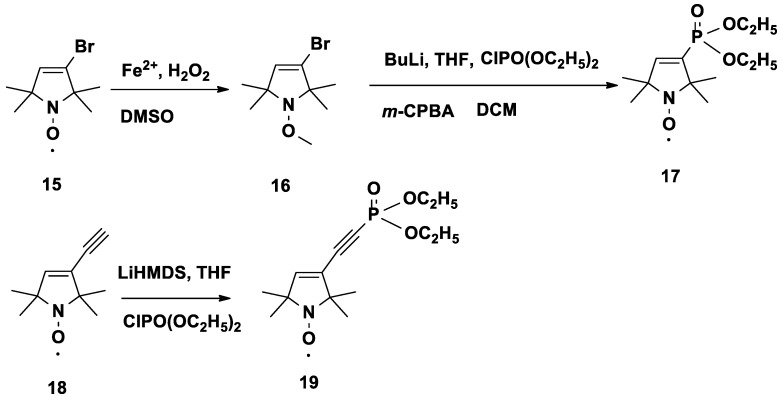
Synthesis of paramagnetic phosphonate esters by lithiation.

To obtain the five-membered vinylphosphonate, we attempted heating of compound **15** [40] with diethylphosphite in the presence of a catalytic amount of NiCl_2_ [41], but no conversion was observed. Our efforts to construct a P-C bond with diethylphospite via the Pd–catalyzed Hirao reaction with the conventional or microwave-assisted method [42] also failed. As a result, we finally decided to lithiate [43] the *O*-methyl derivative **16**, as achieved via Fenton reaction in a dimethylsulfoxide/H_2_O_2_/Fe^2+^ system [44], followed by treatment with 1.0 eq. BuLi (buthyl lithium) and addition of diethylchlorophosphate to produce the diamagnetic vinyl phosphonate, which was not isolated but the crude product was treated with meta-chloroperoxybenzoic acid [45]. Thus we obtained compound **17**, fortunately without epoxidation of the double bond. The paramagnetic acetylene phosphonate can be prepared by deprotonating acetylene **18 [46]** at a terminal acetylene carbon with lithium hexamethyldisilazane (LiHMDS) followed by treatment with diethylchlorophosphate to give compound **19** (Scheme 5). The formation of acetylenephosphonate is proven by the shielded ^31^P signal at −6.4 ppm (see Supplementary Materials).

### 2.5. Horner-Wathsworth-Emmons (HWE) Reactions of Synthesized Paramagnetic Phosphonates

**Scheme 6:**
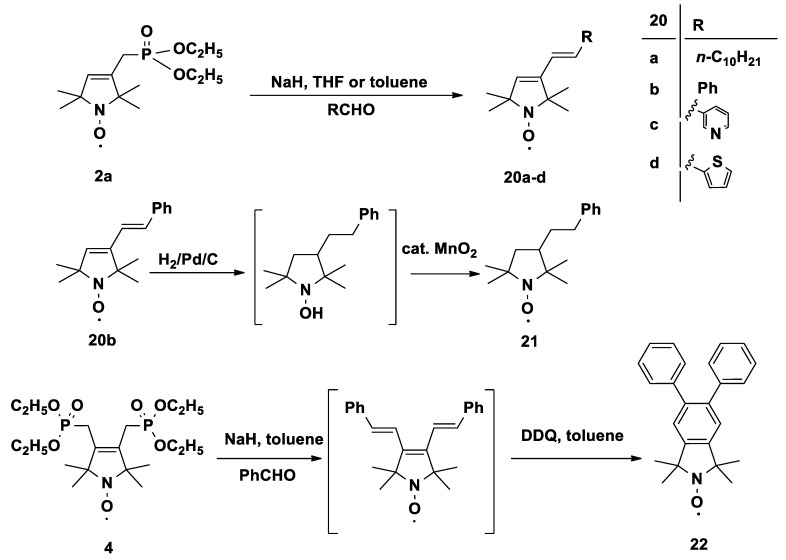
HWE reactions of phosphonates to various alkenes and aromatic compounds including a reduction of **20b** compound to a substituted **21** proxyl nitroxide.

Deprotonation of compound **2a** with sodium hydride in toluene followed by treatment with aliphatic, aromatic or heteroaromatic aldehydes offered *E* paramagnetic alkenes **20a–d**, as proven by the ~16 Hz coupling of the newly formed double bond protons. Saturation of compound **20a** with hydrogen in a continuous flow hydrogenation system (H-Cube Mini Plus) by 10% Pd/C catalyst offered the fully saturated *N*-hydroxylamine, which could be oxidized back to a R,S racemic mixture of 1-oxyl-3-phenethyl-2,2,5,5-tetramethylpyrrolidine radical **21** by a catalytic amount of MnO_2_. Double deprotonation of bisphosphonate witH-NaH followed by addition of an excess of benzaldehyde produced triene, which upon heating spontaneously was cyclized by 6π–electrocyclization to *cis*-5,6-diphenyl-2-oxyl-1,1,3,3-tetramethyl-5,6-dihydro-1*H*-isoindole radical, which partially oxidized to the 5,6-diphenyl-2-oxyl-1,1,3,3-tetramethylisoindoline radical. To complete the oxidation, the worked-up crude product was subjected to oxidation with 2,3-dichloro-5,6-dicyano-1,4-benzoquinone (DDQ) in refluxing benzene to yield **22** isoindoline radical (Scheme 6).

### 2.6. Antioxidant Activity of Nitroxide Phosphonate Esters

The antioxidant (proton and electron donating) activities of phosphonates **2a, 2c** and α-hydroxyphosphonates **8a, 8b, 10a, 10c** were tested [47] in terms of trolox (±)-6-hydroxy-2,5,7,8-tetramethylchromane-2-carboxylic acid equivalent capacity (TEAC). This method is based on reduction of the green-colored 2,2′-azinobis(3-ethylbenzothiazoline-6-sulfonic acid) radical (ABTS^· +^), which is detected at 734 nm. Our results suggest (Table 1) that both the piperidine ring unit (**2c** versus **2a** or **10c** versus **10a**) and hydroxyl group presence (compare **2a** with **10a**) increase the antioxidant activity. The TEAC values of tertiary α-hydroxyphosphonate nitroxides **8a** (0.96) and **8b** (0.93) are almost the same as the trolox activity (1.0) but do not reach the antioxididant activity of 4-hydroxy-1-oxyl-2,2,6,6-tetramethylpiperidin radical (TEMPOL) [48].

## 3. Materials and Methods

### 3.1. General Methods and Reagents

Mass spectra were recorded with a Thermoquest Automass Multi system (ThermoQuest, CE, Instruments, Milan, Italy), a GCMS-2020 (Shimadzu, Tokyo, Japan) both operated in EI mode (70 eV) and a Thermo Q-Exactive HPLC/MS/MS (Thermo Scientific, Waltham, MA, USA) with ESI(+) ionization. Elemental analyses were obtained with a Fisons EA 1110 CHNS elemental analyzer (Fisons Instruments, Milan, Italy). The melting points were determined with a Boetius micromelting point apparatus (Franz Küstner Nachf. K. G., Dresden, Germany). The ^1^H-NMR spectra were recorded with a Bruker Avance 3 Ascend 500 system (Bruker BioSpin Corp., Karslruhe, Germany) operated at 500 MHz, and the ^13^C-NMR spectra were obtained at 125 MHz and ^31^P-NMR 202 MHz in CDCl_3_ or DMSO-*d*_6_ at 298 K. The “in situ” reduction of the nitroxides was achieved by addition of five equivalents of hydrazobenzene ((PhNH)_2_/radical). The *O*-acetyl derivative of compound **22** for NMR measurement was prepared as described previously [49]. The EPR (electron paramagnetic resonance) spectra were recorded on MiniScope MS 200 (Magnettech GMBH, Berlin, Germany) instrument in CHCl_3_ solution, and the concentrations were 1.0 × 10^−4^ M. All radicals gave a 3-line spectra characteristic of monoradicals, a_N_ = 14.4–15.6 G, radical concentration was > 98% in each case and referred for TEMPO (1-oxyl-2,2,6,6-tetramethylpiperidine. The IR spectra were obtained using a Bruker Alpha FT-IR instrument (Bruker Optics, Ettlingen, Germany) with ATR support on a diamond plate. Spectrophotometric measurements were performed on a Specord 40 UV/VIS Spectrophotometer (Specord, Jena, Germany) at 732 nm in a 1 × 1 cm quartz cuvette. Hydrogenation was performed with an H-Cube Mini Plus, ThalesNano, Budapest, Hungary) instrument with a 10%Pd/C cartridge at 5 bar hydrogen pressure, 35 °C, and a flow rate of 1 mL/min. Flash column chromatography was performed on a Kieselgel 60 (0.040–0.063 mm) column (Merck, Darmstadt, Germany). Qualitative TLC was performed on commercially available plates (20 cm × 20 cm × 0.02 cm) coated with Merck Kieselgel GF_254_. Compounds **1a** [19], **1b** [20], **1c** [21], **3** [22], **5a** [33], **5b** [23], **6** [25], **9a** [34], **9b** [20], **9c** [21], **15** [40], **18** [46], TEMPO [23] and TEMPOL [23] were synthesized as previously described. The reagents LiHMDS, Trolox^®^, *m*-CPBA, diethylphosphite, triphenyl-phosphine, triethylphosphite, DEAD, FeCl_3,_ MnO_2_, NaH_,_ NaN_3_, DDQ, POCl_3_, ABTS, Dess–Martin periodinane, benzaldehyde, 2-thiophencarbaldehyde, undecanal, 3-pyridinecarbaldehyde, NiCl_2_, diethyl chlorophosphate, BuLi, DMSO-*d*_6_, CDCl_3_, hydrazobenzene were purchased from Sigma Aldrich (St. Louis, MO, USA) and hexane, DCM, CHCl_3_, methanol (MeOH), methyliodide (MeI), ethyl acetatate (EtOAc), toluene, benzene, THF, MgSO_4_, FeSO_4_^.^7H_2_O, NaCl, Na_2_HPO_4_, KH_2_PO_4_ from Molar Chemicals (Halásztelek, Hungary).

### 3.2. General Procedure for Arbusov Reactions *(**2a–c, 4**)*

In a well-ventilated hood, a mixture of compound **1a** or **1b** or **1c** or **3** (10.0 mmol) and triethylphosphite (2.5 g, 15.0 mmol, or 5.0 g, 30.0 mmol, for compound **3**) was stirred in an open vessel at 120 °C in an oil bath. The ethylbromide byproduct was allowed to escape. The reaction mixture was monitored by TLC, and after consumption of the starting material (~2 h), the mixture was allowed to cool spontaneously with stirring. After cooling, the resulting mixture was purified by flash column chromatography to give the allylic phosphonates.

#### 3.2.1. Diethyl ((1-oxyl-2,2,5,5-tetramethyl-2,5-dihydro-1*H*-pyrrol-3-yl)methyl)phosphonate Radical (**2a**)

Purified by flash column chromatography (eluent: hexane/EtOAc, 1:1) to produce an orange oil (1.88 g, 65%); TLC (CHCl_3_/Et_2_O, 2:1): R*_f_* = 0.33. ^31^P-NMR (CDCl_3_ + (PhNH)_2_) δ:26.9. ^13^C-NMR (CDCl_3_ + (PhNH)_2_) δ: 16.6 (d, *J* = 6.0 Hz, 2C), 24.2 (2C), 24.3 (d, *J* = 143.0 Hz, 1C), 25.8 (2C), 62.2 (d, *J* = 6.6 Hz, 2C), 68.2 (d, *J* = 1.1 Hz, 1C) 71.6 (d, *J* = 9 Hz, 1C), 132.6 (d, *J* = 8.0 Hz, 1C), 134.0 (d, *J* = 8.0 Hz, 1C). ^1^H-NMR (CDCl_3_ + (PhNH)_2_) δ: 1.32 (s, 6H), 1.36 (s, 6H), 1.39 (t*, J* = 6.9 Hz, 6H), 2.56 (d, *J* = 22 Hz, 2H), 4.19 (quint, *J* = 1.2 Hz, 4H), 5.86 (s, 1H). IR: 2976, 2931, 1650 cm^−1^. MS (EI): *m*/*z* (%): 290 (M^+^, 13) 260 (70), 245 (15), 138 (22), 122 (100).

#### 3.2.2. Diethyl ((4-bromo-1-oxyl-2,2,5,5-tetramethyl-2,5-dihydro-1*H*-pyrrol-3-yl)methyl)phosphonate Radical (**2b**)

Purified by flash column chromatography (eluent: hexane/EtOAc, 1:1) to afford an orange oil (2.83 g, 77%); TLC (CHCl_3_/Et_2_O, 2:1): R*_f_* =0.48. ^31^P-NMR (CDCl_3_ + (PhNH)_2_) δ:26.9. ^13^C-NMR (CDCl_3_ + (PhNH)_2_) δ: 16.5 (d, *J* = 6.0 Hz, 2C), 24.1 (d, *J* = 143.0 Hz, 1C), 24.2 (2C), 25.8 (2C), 62.1 (d, *J* = 6.7 Hz, 2C), 68.0 (d, *J* = 2.1 Hz, 1C) 71.4 (d, *J* = 8.8 Hz, 1C), 132.6 (d, *J* = 8.1 Hz, 1C), 133.9 (d, *J* = 11.1 Hz, 1C). ^1^H-NMR (CDCl_3_ + (PhNH)_2_) δ: 1.35 (s, 6H), 1.37 s (6H), 1.45 (bs, 6H)., 2.58 (d, *J* = 21.5 Hz, 2H), 4.25 (bs, 4H). IR: 2979, 2932, 1644 cm^−1^. MS (EI): *m*/*z* (%): 370/368 (M^+^, 44), 340/338 (4/4), 259(35), 121 (100).

#### 3.2.3. Diethyl ((1-oxyl-2,2,6,6-tetramethyl-1,2,3,6-tetrahydropyridin-4-yl)methyl)phosphonate Radical (**2c**)

Obtained by method A: Purified by flash column chromatography (eluent: hexane/EtOAc, 1:1) to afford a red oil (2.46 g, 81%); TLC (CHCl_3_/Et_2_O, 2:1): R*_f_* = 0.35. ^31^P-NMR (CDCl_3_ + (PhNH)_2_) δ: 27.1. ^13^C-NMR (CDCl_3_ + (PhNH)_2_) δ: 16.5 (d, *J* = 6.1 Hz, 2C),25.0 (1C), 26.3 (bs, 1C), 34.3 (d, *J* = 38.1 Hz, 2C), 44.0 (d, *J* = 2.3 Hz, 1C), 57.7 (1C), 59.0 (d, *J* = 2.3 Hz, 1C), 61.9 (d, *J* = 6.8 Hz, 2C), 122.5 (d, *J* = 11.0 Hz, 1C), 134.1 (d, *J* = 12.0 Hz, 1C). ^1^H-NMR (CDCl_3_ + (PhNH)_2_) δ: 1.28 (s, 6H), 1.32 (s, 6H), 1.38 (t, *J* = 7 Hz, 6H), 2.29 (d, *J* = 3.5 Hz, 2H), 2.55 (d, *J* = 21.5 Hz, 2H), 4.13–4.20 (m, 4H), 5,43 (d, *J* = 5.3 Hz, 1H). IR: 2977, 2932, 1645 cm^−1^. MS (EI): *m*/*z* (%): 304 (M^+^, 27) 274 (100), 259 (27), 152 (16), 81 (60).

#### 3.2.4. Diethyl ((1-oxyl-2,2,6,6-tetramethyl-1,2,3,6-tetrahydropyridin-4-yl)methyl)phosphonate Radical (**2c**)

Obtained by method B: To a stirred suspension of NaH (240 mg, 10.0 mmol) in toluene (10 mL), a solution of tetraethyl methylenediphosphonate (2.88 mg, 10.0 mmol) in toluene (10 mL) was added dropwise at 0 °C under N_2_. After 30 min, a solution of compound **5b** (1.7 g, 10.0 mmol) in toluene (10 mL) was added dropwise at 0 °C. The mixture was refluxed for 3 hours. After cooling, the solvent was evaporated, and the residue was partitioned between water (30 mL) and EtOAc (50 mL). The organic phase was separated, dried (MgSO_4_), filtered, and evaporated, and the residue was purified by flash column chromatography (eluent: hexane/EtOAc, 1:1) to give a red oil (1.77 g, 58%), TLC (CHCl_3_/Et_2_O 2:1): R*_f_* = 0.35. IR: 2977, 2932, 1645 cm^−1^, and all other spectral data were identical to those of one of the compounds obtained with method A.

#### 3.2.5. Tetraethyl ((1-oxyl-2,2,5,5-tetramethyl-2,5-dihydro-1*H*-pyrrole-3,4-diyl)bis(methylene)) bisphosphonate Radical (**4**)

Purified by flash column chromatography (eluent: CHCl_3_/Et_2_O, 2:1) to give a brownish powder (3.1 g, 70%); mp 85–87 °C; TLC (CHCl_3_/MeOH 29:1): R*_f_* = 0.33. ^31^P-NMR (DMSO-*d*_6_ + (PhNH)_2_) δ:27.4. ^13^C-NMR ((DMSO-*d*_6_ + (PhNH)_2_) δ: 16.7 (4C), 23.6 (d, *J* = 133.0 Hz, 2C), 24.7 (4C), 61.7 (4C), 69.4 (2C), 132.7 (2C). ^1^H-NMR (DMSO-*d*_6_ + (PhNH)_2_) δ: 1.11 (s, 12H), 1.23 (t, *J* = 6.8 Hz, 12H), 2.92 (d, *J* = 20.0 Hz, 4H), 4.01 (quint, *J* = 6.5 Hz, 8H). IR: 2982, 2933, 2920 cm^−1^. MS (EI): *m*/*z* (%): 440 (M+, 10), 410 (38), 395 (28), 273 (77), 152 (8), 135 (100)

### 3.3. Diethyl (1-oxyl-2,2,6,6-tetramethyl-1,2,3,6-tetrahydropyridin-4-yl)phosphate Radical *(**7**)*

In a well-ventilated hood, a mixture of compound **6** (2.49 g, 10.0 mmol) and triethylphosphite (2.5 g, 15.0 mmol) was stirred in an open vessel at 60 °C in an oil bath. The ethylbromide byproduct was allowed to escape. The reaction mixture was monitored by TLC, and after ~ 2 h, the temperature was increased to 100 °C for ~ 1 h. The mixture was allowed to cool spontaneously with stirring. After cooling, the resulted mixture was purified by flash column chromatography to give the Perkow product, which was purified by flash column chromatography (hexane/EtOAc, 1:1) to give a red oil (1.05 g, 34%); TLC (CHCl_3_/Et_2_O, 2:1): R*_f_* = 0.50. ^31^P-NMR (CDCl_3_ + (PhNH)_2_) δ: −6.2. ^13^C-NMR ((CDCl_3_ + (PhNH)_2_) δ: 16.2 (d, *J* = 6.5 Hz, 2C), 25.3 (2C), 26.7 (2C), 42.1 (d, *J* = 3.8 Hz, 1C), 58.4 (1C), 59.1 (1C), 64.3 (d, *J* = 6.1 Hz, 2C), 118.0 (d, *J* = 5.4 Hz, 1C), 142.3 (d, *J* = 8.8 Hz, 1C). ^1^H-NMR (DMSO-*d*_6_ + (PhNH)_2_) δ: 1.30 (s, 6H), 1.35 (s, 6H), 1.43 (t, *J* = 7.1 Hz, 6H), 2.38 (s, 2H), 4.23 (quint, *J* = 7.1 Hz, 2H), 5.43 (d, *J* = 1.8 Hz, 1H). IR: 2980, 2935, 2911, 1696 cm^−1^. MS (EI): *m*/*z* (%): 306 (M^+^, 8), 276(10), 155 (70) 107 (100).

### 3.4. General Procedure for Pudovik α-hydroxyphosphonate Synthesis from Paramagnetic Aldehydes and Ketones ***8a, 8b, 10a–c***

To a stirred mixture of compound **5a** or **5b** or **9a** or **9b** or **9c** and diethyl phosphite (1.38 g, 10.0 mmol), K_3_PO_4_ (106 mg, 0.5 mmol) was added, and the stirring continued at room temperature for 1 h. Subsequently, 10% aq. Na_2_CO_3_ (50 mL) was added, followed by extraction with EtOAc (2 × 50 mL). The combined organic phases were dried (MgSO_4_), filtered, and evaporated, and the residue was purified by flash column chromatography (eluent: hexane/EtOAc, 1:1) to give the α-hydroxy-phosphonate products.

#### 3.4.1. Diethyl (3-hydroxy-1-oxyl-2,2,5,5-tetramethylpyrrolidin-3-yl)phosphonate Radical (**8a**)

Purified by flash column chromatography (eluent: CHCl_3_/Et_2_O, 2:1) to give a yellow powder (2.7 g, 92%); mp 100–103 °C; TLC (CHCl_3_/MeOH, 56:4): R*_f_* = 0.51. ^31^P-NMR (DMSO-*d*_6_ (PhNH)_2_) δ: 23.2. ^13^C-NMR ((DMSO-*d*_6_ + (PhNH)_2_) δ: 17.0 (d, *J* = 5.2 Hz, 2C), 20.0 (1C), 22.1 (1C), 27.0 (1C), 31.1 (1C), 46.5 (d, *J* = 4.0 Hz, 1C), 61.9 (d, *J* = 8.2 Hz, 1C), 62.6 (d, *J* = 5.6 Hz, 1C), 77.8 (1C), 79.1 (1C). ^1^H-NMR (DMSO-*d*_6_ + (PhNH)_2_) δ: 1.11–1.25 (m, 18H), 1.85 (d, *J* = 13.4 Hz, 1H), 2.35 (t, *J* = 11.9 Hz, 1H), 4.04–4.11 (m, 4H). IR: 3258, 2982, 2938, 2910 cm^−1^. MS (EI): *m*/*z* (%): 294 (M^+^, 12), 264(2), 249 (5) 180 (100), 138 (78).

#### 3.4.2. Diethyl (4-hydroxy-1-oxyl-2,2,6,6-tetramethylpiperidin-4-yl)phosphonate Radical (**8b**)

Purified by flash column chromatography (eluent: CHCl_3_/Et_2_O, 2:1) to give red crystals (2.77 g, 90%); mp 115–117 °C; TLC (CHCl_3_/MeOH, 56:4): R*_f_* = 0.53. ^31^P-NMR (CDCl_3_ (PhNH)_2_) δ: 24.4. ^13^C-NMR ((CDCl_3_ + (PhNH)_2_) δ: 16.6 (d, *J* = 5.1 Hz, 2C), 21.0 (4 C), 33.3 (2C), 43.1 (2C), 57.9 (d, *J* = 14.5 Hz, 1C), 63.1 (d, *J* = 7.5 Hz), 71.3 (1C), 72.6 (1C). ^1^H-NMR (CDCl_3_ + (PhNH)_2_) δ: 1.28 (s, 6H), 1.40 (t, *J* = 7 Hz, 6H), 1.48 (s, 6H), 2.02 (d, *J* = 4.01 Hz, 4H), 3.11 (bs, 1H), 4.23 (quint, *J* = 7.1 Hz, 4H), 4.69 (bs, 1H). IR: 3198, 2993, 2973, 2929 cm^−1^. MS (EI): *m*/*z* (%): 308 (M^+^, 13), 259(10), 222 (38), 194 (16), 156 (18), 138 (100).

#### 3.4.3. Diethyl (hydroxy(1-oxyl-2,2,5,5-tetramethyl-2,5-dihydro-1*H*-pyrrol-3-yl)methyl)phosphonate Radical (**10a**)

Purified by flash column chromatography (eluent: hexane/EtOAc, 1:1) to give an orange oil (2.61 g, 85%); TLC (CHCl_3_/MeOH, 58:2): R*_f_* = 0.33. ^31^P-NMR (CDCl_3_ (PhNH)_2_) δ: 21.8. ^13^C-NMR ((CDCl_3_ + (PhNH)_2_) δ: 16.5 (t, *J* = 5.1 Hz, 2C), 24.5. (1C), 24.9 (1C), 25.4 (1C), 25.5 (1C), 63.1 (d, *J* = 185.0 Hz, 1C), 63.9 (d, *J* = 164.0 Hz, 2C), 68.0 (1C), 71.2 (d, *J* = 9.4 Hz, 1C), 135.1 (d, *J* = 6.2 Hz, 1C), 140.3 (1C). ^1^H-NMR (CDCl_3_ + (PhNH)_2_) δ: 1.34–1.42 (m, 18H), 4.26 (q, *J* = 7.0 Hz, 4H), 4.35 (d, *J* = 10.8 Hz, 1H), 6.13 (s, 1H). IR: 3286, 2977, 2931, 1645 cm^−1^. MS (EI): *m*/*z* (%): 306 (M^+^, 7), 276 (9), 154 (26), 138 (100).

#### 3.4.4. Diethyl (hydroxyl(4-bromo-1-oxyl-2,2,5,5-tetramethyl-2,5-dihydro-1*H*-pyrrol-3-yl)methyl)phosphonate Radical (**10b**)

Purified by flash column chromatography (eluent: hexane/EtOAc, 1:1) to give an orange powder (2.98 g, 78%); mp 107–109 °C; TLC (CHCl_3_/MeOH, 58:2): R*_f_* = 0.34. ^31^P-NMR (CDCl_3_ (PhNH)_2_) δ: 20.7. ^13^C-NMR ((CDCl_3_ + (PhNH)_2_) δ: 16.5 (d, *J* = 5.7 Hz, 2C), 23.7 (1C), 24.5 (1C) 24.9 (1C), 25.1 (1C), 63.0 (d, *J* = 7.2 Hz, 1C) 63.6 (d, *J* = 7.2 Hz, 1C), 67.5 (d, *J* = 162.1 Hz, 1C), 70.8 (1C), 71.5 (1C), 127.1 (d, *J* = 12.6 Hz, 1C), 137.5 (1C). ^1^H-NMR (CDCl_3_ + (PhNH)_2_) δ: 1.33–1.47 (m, 18H), 4.18-4.30 (m, 4H), 4.94 (d, *J* = 16.7 Hz, 1H). IR: 3263, 2980, 2934, 2908, 1631 cm^−1^. MS (EI): *m*/*z* (%): 386/384 (M^+^, 16/16), 356/354 (4/4), 275 (38), 138 (100).

#### 3.4.5. Diethyl (hydroxy(1-oxyl-2,2,6,6-tetramethyl-1,2,3,6-tetrahydropyridin-4-yl)methyl)-phosphonate Radical (**10c**)

Purified by flash column chromatography (eluent: hexane/EtOAc, 1:1) to give a red oil (2.56 g, 80%); TLC (CHCl_3_/MeOH, 58:2): R*_f_* = 0.38. ^31^P-NMR (CDCl_3_ (PhNH)_2_) δ: 22.0. ^13^C-NMR ((CDCl_3_ + (PhNH)_2_) δ: 16.5 (d, *J* = 5.7 Hz, 2C), 39.8. (1 C), 57.7 (1C), 59.8 (1C), 62.8 (d, *J* = 7.4 Hz, 1C) 63.1 (d, *J* = 7 Hz, 1C), 71.3 (d, *J* = 158.1 Hz, 1C), 127.4 (d, *J* = 4.3 Hz, 1C), 132.8 (d, *J* = 11.5 Hz, 1C). ^1^H-NMR (CDCl_3_ + (PhNH)_2_) δ: 1.27 (s, 6H), 1.33 (s, 6H), 1.34 (s, 6H), 1.38 (t, *J* = 7 Hz, 3H), 2.30 (dq, *J_1_*= 2.5 Hz, *J_2_*=9.9 Hz, 2H), 4.18–4.27 (m, 4H), 4.38 (d, *J* = 10 Hz, 1H), 5.67 (d, *J* = 4.6 Hz, 1H). IR: 3290, 2978, 2933, 1649 cm^−1^. MS (EI): *m*/*z* (%): 320 (M^+^, 5), 290 (7), 272 (8), 182 (10), 152 (100).

### 3.5. Diethyl (1-oxyl-2,2,6,6-tetramethyl-1,2,3,6-tetrahydropyridin-4-yl)phosphonate Radical *(**11**)*

To a solution of compound **8b** (1.54 g, 5.0 mmol) in anhydrous pyridine (10 mL), POCl_3_ (1.0 mL, 10.6 mmol) was added dropwise at 0 °C, and the mixture was allowed to remain at r.t for 48 h. The mixture was poured onto 100 g crushed ice, extracted with CH_2_Cl_2_ (3 × 15 mL), and the combined organic phase was washed with aq. 1N HCl (2 × 40 mL). The organic phase was dried (MgSO_4_), filtered, and evaporated, and the residue was purified by flash column chromatography (eluent: hexane/EtOAc, 2:1) to give a red powder (420 mg, 29%); mp 53–55 °C; TLC (CHCl_3_/Et_2_O, 2:1): R*_f_* = 0.44. ^31^P-NMR (CDCl_3_ (PhNH)_2_) δ: 19.3. ^13^C-NMR ((CDCl_3_ + (PhNH)_2_) δ: 16.4 (d, *J* = 6.1 Hz, 2C), 24.6 (2C), 25.5 (2C), 39.1. (d, *J* = 7.3 Hz, 1C), 57.3 (d, *J* = 4.9 Hz, 1C), 60.5 (d, *J* = 17.8 Hz, 1C), 61.7 (d, *J* = 5.4 Hz, 1C), 120.5 (d, *J* = 182.6 Hz, 1C), 149.3 (d, *J* = 7.6 Hz, 1C). ^1^H-NMR (CDCl_3_ + (PhNH)_2_) δ: 1.27 (s, 6H), 1.38–1.41 (m, 12H), 2.31 (d, *J* = 6.1 Hz, 2H), 4.10-4.2 (m, 4H), 6.6 (d, *J* = 21.5 Hz, 1H). IR: 2979, 2932, 2903, 1658 cm^−1^. MS (EI): *m*/*z* (%): 320 (M^+^, 5), 290 (7), 272 (8), 182 (10), 152 (100).

### 3.6. Diethyl (1-oxyl-2,2,5,5-tetramethyl-2,5-dihydro-1H-pyrrole-3-carbonyl)phosphonate Radical *(**12**)*

To a stirred solution of compound **10a** (1.53 g, 5.0 mmol) in anhydr. CH_2_Cl_2_ (DCM) (10 mL), Dess–Martin periodinane (6.36 g, 15.0 mmol, 3 eq.) was added in 3 portions at 0 °C over a period of 10 min. The stirring was continued for 1 h at r.t. The resulting mixture was filtered on a sintered glass funnel. The filtrate was diluted with DCM (25 mL) and washed with 10% aq NaHCO_3_ solution (25 mL) and 10% aq Na_2_S_2_O_3_ (25 mL). The organic phase was separated, dried (MgSO_4_), filtered, and evaporated, and the residue was purified by flash column chromatography (eluent: hexane/EtOAc, 1:1) to give a red powder (950 mg, 62%); mp 35–37 °C; TLC (CHCl_3_/Et_2_O, 2:1): R*_f_* = 0.56, ^31^P-NMR (CDCl_3_ (PhNH)_2_) δ: −2.9. ^13^C-NMR ((CDCl_3_ + (PhNH)_2_) δ: 16.4 (d, *J* = 5.7 Hz, 2C), 24.3. (2 C), 24.7 (2C) 63.9 (d, *J* = 7.3 Hz, 2C), 69.0 (1C), 70.3 (d, *J* = 10.8 Hz, 1C), 143.2 (d, *J* = 64.0 Hz, 1C), 155.2 (1C), 196 (d, *J* = 174.0 Hz, 1C). ^1^H-NMR (CDCl_3_ + (PhNH)_2_) δ: 1.42-1.46 (m, 18H), 4.28 (quint, *J* = 7.24 Hz, 4H). IR: 3067, 2976, 2931, 1637, 1601 cm^−1^. MS (EI): *m*/*z* (%): 304 (M^+^, 4), 274 (6), 246 (3), 137 (49), 109 (100).

### 3.7. Diethyl (azido (1-oxyl-2,2,5,5-tetramethyl-2,5-dihydro-1H-pyrrol-3-yl)methyl)phosphonate Radical *(**13**)*

To a stirred suspension of Ph_3_P (3.14 g, 12 mmol) in anhydrous DCM (10 mL), a solution of DEAD 5.2 mL (2.09 g, 12.0 mmol in 40% toluene) diluted anhydr. DCM (5 mL) was added dropwise at −78 °C under N_2_. A 1.85 M solution of HN_3_ (6.7 mL, 12.5 mmol) in benzene was added dropwise, and the stirring was continued for 5 min at 0 °C followed by dropwise addition of a solution of compound **10a** (3.06 g, 10.0 mmol) in anhydr. DCM (10 mL). After the addition was completed, the mixture was held for 30 min at 0 °C, and stirring was continued for 24 h at r.t. The resulted mixture was filtered on a sintered glass funnel, and solvents were evaporated. The residue was purified by flash column chromatography (eluent: hexane/EtOAc, 2:1) to give a yellow powder (1.97 g, 60%); mp 50–52 °C; TLC (CHCl_3_/Et_2_O, 2:1): R*_f_* = 0.60, ^31^P-NMR (CDCl_3_ (PhNH)_2_) δ: 15.6. ^13^C-NMR ((CDCl_3_ + (PhNH)_2_) δ: 16.4 (t, *J* = 5.7 Hz, 2C), 19.7 (1 C), 22.4 (1C) 25.0 (1C), 30.9 (1C), 61.7 (d, *J* = 5.6 Hz, 1C), 62.0 (d, *J* = 5.6 Hz, 1C), 66.1 (1C), 66.3 (1C), 66.5 (d, *J* = 6.4 Hz, 1c), 114.1 (d, *J* = 191 Hz, 1C), 167.0 (d, *J* = 5.4 Hz, 1C). ^1^H-NMR (CDCl_3_ + (PhNH)_2_) δ: 1.38–1.43 (m, 18H), 4.12–4.28 (m, 4H), 4.98 (s, 1H), 5.81 (d, *J* = 13.1 Hz). IR: 2981, 2935, 2096, 1635 cm^−1^. HRMS (ESI) *m*/*z* [M+H]^+^ calc. for C_13_H_25_N_4_O_4_P: 332.1613; found: 332.1609.

### 3.8. Diethyl ((1-oxl-2,2,5,5-tetramethyl-2,5-dihydro-1H-pyrrol-3-yl)iodomethyl)phosphonate Radical *(**14**)*

To a stirred suspension of compound **10a** (3.06 g, 10.0 mmol) and Ph_3_P (3.14 g, 12.0 mmol) in benzene (20 mL), a solution of DEAD (2.09 g, 12.0 mmol in 40% toluene) diluted with benzene (5 mL) was added dropwise at 0 °C under N_2_. After 10 min to complete the addition, a solution of CH_3_I (0.7 mL, 12.0 mmol) in benzene (5 mL) was added dropwise. After the addition was completed, the mixture was held for 30 min at 0 °C, and stirring was continued for 24 h at r.t. The solvent was evaporated, and the residue was partitioned between water (20 mL) and EtOAc (50 mL). The organic phase was separated, dried (MgSO_4_), filtered, and evaporated, and the crude was purified by flash column chromatography (eluent: hexane/EtOAc, 1:1) to give a yellow semi-solid (2.0 g, 48%); TLC (CHCl_3_/Et_2_O, 2:1): R*_f_* = 0.40, IR: 3040, 2990 1528. HRMS (ESI) *m*/*z* [M]^+^ calc. for C_13_H_25_INO_4_P: 417.0566; found: 417.1311; [M-HI]^+^ calc. for C_13_H_24_NO_4_P: 289.1443; found 289.1434.

### 3.9. 3-Bromo-1-methoxy-2,2,5,5-tetramethyl-2,5-dihydro-1H-pyrrole *(**16**)*

To a stirred solution of **15** (1.1 g, 5.0 mmol) and FeSO_4_·7H_2_O (6.9 g, 25.0 mmol) in DMSO (30 mL) at 0 °C, 30% aq H_2_O_2_ (5 mL) was added dropwise over 2 h. The reaction was monitored by TLC. Upon consumption of the starting material, distilled H_2_O (50 mL) was added, and the aqueous solution was extracted with Et_2_O (3 × 30 mL). The combined organic phases were dried (MgSO_4_), filtered, and evaporated, and the crude product was purified by flash column chromatography (hexane–Et_2_O, 2:1) to give a colorless oil (700 mg, 60%); TLC (hexane/Et_2_O, 9:1): R*_f_* = 0.42. ^13^C-NMR (CDCl_3_) δ: 22.3 (2C) 28.6 (2C), 65.0 (1C) 68.9 (1C), 71.7 (1C), 125.6 (1C), 134.0 (1C). ^1^H-NMR (CDCl_3_) δ: 1.27 (s, 6H), 1.29 (s, 6H), 3.69 (s, 3H), 5.69 (s, 1H). IR: 2921, 2852, 1642. MS (EI): *m*/*z* (%): 235/233 (M^+^, 3/3), 220/218 (33/33), 139 (100), 108 (25).

### 3.10. Diethyl (1-oxyl-2,2,5,5-tetramethyl-2,5-dihydro-1H-pyrrol-3-yl)phosphonate Radical *(**17**)*

To a stirred solution of compound **16** (470 mg, 2.0 mmol) in anhydrous THF (10 mL), *n*-BuLi solution in hexane (0.8 mL, 2.0 mmol, 2.5 M) diluted with anhydr. THF (10 mL) was added dropwise at −78 °C under N_2._. After the addition was completed, the mixture was continuously stirred for 1 h at −78 °C. A solution of diethylchlorophosphate (345 mg, 2.0 mmol) in anhydr. THF (10 mL) was added dropwise. After stirring at this temperature for 30 min, the reaction mixture was allowed to warm to r.t. with continuous stirring for 2 h. A sat. aq. NH_4_Cl solution (5 mL) was added, the mixture was extracted with CH_2_Cl_2_ (2 × 10 mL), and the combined organic phase was dried (MgSO_4_), filtered and evaporated. The crude residue (480 mg, 1.65 mmol) was dissolved in anhydr. DCM (10 mL), and 3-chloroperbenzoic acid (~60%, 1.18 g, 4.1 mmol, 2.5 eq) was added in 2–3 portions at 0 °C over a period of 10 min. Stirring was continued for an additional 1 h at ambient temperature. The solution was washed with 10% aq. Na_2_CO_3_ solution (2 × 20 mL), and the organic phase was separated, dried (MgSO_4_), filtered and evaporated. The residue was purified by flash column (eluent: hexane/EtOAc, 1:1) to give a yellow powder (140 mg, 50%); mp 60–62 °C; TLC (CHCl_3_/MeOH, 2:1): R*_f_* = 0.50. ^31^P-NMR (CDCl_3_ (PhNH)_2_) δ: 14.6. ^13^C-NMR ((CDCl_3_ + (PhNH)_2_) δ: 16.3 (d, *J* = 6.3 Hz, 2C), 25.0 (2C), 25.3 (2C), 61.9 (d, *J* = 5.6 Hz, 2C), 68.7 (d, *J* = 15.6 Hz, 1C), 71.3 (d, *J* = 15.6 Hz, 1C), 133.8 (d, *J* = 4.0 Hz, 1C), 150.6 (d, *J* = 8.1 Hz, 1C). ^1^H-NMR (CDCl_3_ + (PhNH)_2_) δ: 1.33 (s, 6H), 1.40 (t, *J* = 7.5 Hz, 6H), 1.44 (s, 6H) 4.13–4.21 (m, 4H), 6.57 (d, *J* = 13.5 Hz, 1H). IR: 3079, 2977, 2931, 2866, 1609 cm^−1^. MS (EI): *m*/*z* (%): 276 (M^+^, 15), 246 (65), 231 (100), 203 (5), 175 (44), 107 (78).

### 3.11. Diethyl ((1-oxyl-2,2,5,5-tetramethyl-2,5-dihydro-1H-pyrrol-3-yl)ethynyl)phosphonate Radical *(**19**)*

To a stirred solution of compound **18** (492 mg, 3.0 mmol) in anhydr. THF (10 mL), LiHMDS (3.0 mL 3.0 mmol, 1 M THF solution) was added dropwise at −78 °C under N_2._. After the addition was completed, the mixture was stirred for 1 h at −78 °C. A solution of diethylchlorophosphate (517 mg, 3.0 mmol) in anhydr. THF (10 mL) was added dropwise, and the temperature was allowed to warm to r.t. spontaneously with stirring for 2 h. The reaction mixture was quenched with sat. NH_4_Cl solution (5 mL). The mixture was diluted with EtOAc (20 mL), the organic phase was separated, the aq. phase was extracted with EtOAc (10 mL), and the combined phases were dried (MgSO_4_), filtered and evaporated. The residue was subjected to flash column chromatography purification (eluent: hexane/EtOAc, 1:1) to offer a yellow solid (470 mg, 52%); mp 50–52 °C; TLC (CHCl_3_/Et_2_O, 2:1): R*_f_* = 0.43. ^31^P-NMR (CDCl_3_ (PhNH)_2_) δ: –6.4. ^13^C-NMR ((CDCl_3_ + (PhNH)_2_) δ: 16.1 (d, *J* = 6.9 Hz, 2C), 24.9 (2C), 25.2 (2C), 63.2 (d, *J* = 5.5 Hz, 2C), 69.2 (1C), 71.3 (1C), 81.7 (d, *J* = 297.8 Hz, 1C), 93.8 (d, *J* = 52.7 Hz, 1C), 125.6 (d, *J* = 3.6 Hz, 1C), 146.2 (d, *J* = 3.0 Hz, 1C). ^1^H-NMR (CDCl_3_ + (PhNH)_2_) δ: 1.32 (s, 6H), 1.38 (s, 6H) 1.45 (t, *J* = 7.1 Hz, 6H), 4.22–4.28 (m, 4H), 6.22 (d, *J* = 0.7 Hz, 1H). IR: 3073, 2976, 2931, 2908, 2866, 2171, 1612 cm^−1^. MS (EI): *m*/*z* (%): 300 (M^+^, 14), 285 (33), 270 (20), 241 (7), 132 (100), 117 (52).

### 3.12. General Procedure for HWE Olefination with **2a** Nitroxide Phosphonate: Compounds ***20a–d***

To a stirred suspension of oil-free NaH (120 mg, 5.0 mmol) in anhydr. toluene (10 mL), a solution of compound **2a** (1.45 g, 5.0 mmol) in anhydr. toluene (5 mL) was added dropwise at 0 °C under N_2_. After 30 min, a solution of the appropriate aldehyde (5.0 mmol) in anhydr. toluene (10 mL) was added dropwise at 0 °C. The mixture was refluxed for 3 h and allowed to stand overnight at r.t. The solvent was evaporated, and the residue was partitioned between sat. aq. NH_4_Cl solution (25 mL) and EtOAc (50 mL). The organic phase was separated, dried (MgSO_4_), filtered, and evaporated, and the crude product was purified by flash column chromatography to yield the olefinated nitroxides.

#### 3.12.1. (*E*)-3-(Dodec-1-en-1-yl)-1-oxyl-2,2,5,5-tetramethyl-2,5-dihydro-1*H*-pyrrole Radical (**20a**)

Purified by flash column chromatography (eluent: hexane/Et_2_O, 2:1) to give a brown oil (950 mg, 62%); TLC (hexane/Et_2_O, 5:1): R*_f_* = 0.56. ^13^C-NMR ((CDCl_3_ + (PhNH)_2_) δ: 24.9 (2C), 25.0 (2C) 25.7 (1C), 29.0 (1C), 29.1 (1C), 29.2 (1C), 29.3 (1C)29.4 (1C), 29.5 (1C), 33.3 (1C), 33.8 (1C), 65.4 (1C), 67.4 (1C), 114.2 (1C), 130.8 (1C), 131.25 (1C), 139.1 (1C). 139.2 (1C). ^1^H-NMR (CDCl_3_ + (PhNH)_2_) δ: 1.33–1.37 (m, 33H), 2.13 (d, J = 6.1 Hz, 2H) 5.02 (d, J =10.0 Hz, 1H), 5.08 (d, J = 17 Hz, 1H), 5.88–5.95 (m, 1H). IR: 3075, 2975, 2924, 2853, 1640 cm^−1^. MS (EI): *m*/*z* (%): 306 (M^+^, 2), 281 (7), 207 (28), 149 (25), 55 (100).

#### 3.12.2. (*E*)-1-Oxyl-3-styryl-2,2,5,5-tetramethyl-2,5-dihydro-1*H*-pyrrole Radical (**20b**)

Purified by flash column chromatography (eluent: hexane/Et_2_O, 2:1) to give a to give an orange powder; mp 67–70 °C (730 mg, 60%); TLC (hexane/Et_2_O, 2:1): R*_f_* = 0.53. ^13^C-NMR ((CDCl_3_ + (PhNH)_2_) δ: 25.4 (2C), 26.0 (2C), 67.6 (1C), 70.3 (1C), 122.4 (1C), 126.4 (2C), 127.7 (1C), 128.8 (2C), 129.9 (1C), 131.9 (1C), 137.4 (1C), 142.7 (1C). ^1^H-NMR (CDCl_3_ + (PhNH)_2_) δ: 1.45 (s, 6H), 1.56 (s, 6H) 5.86 (s, 1H), 6.7 (d, *J* = 16.5 Hz, 1H), 7.36-7.47 (m, 3H). 3H are overlapped with peaks of diphenyl hydrazine. IR: 3023, 2972, 2927, 2865, 1634, 1596 cm^−1^. MS (EI): *m*/*z* (%): 242 (M^+^, 12), 227 (22), 212 (100), 197 (71), 91 (28).

#### 3.12.3. (*E*)-1-Oxyl-3-(2-(pyridin-3-yl)vinyl)-2,2,5,5-tetramethyl-2,5-dihydro-1*H*-pyrrole Radical (**20c**)

Purified by flash column chromatography (eluent: hexane/Et_2_O, 2:1) to give an orange powder; mp 90–93 °C (680 mg, 56%); TLC (CHCl_3_/Et_2_O, 2:1): R*_f_* = 0.33. ^13^C-NMR ((CDCl_3_ + (PhNH)_2_) δ: 25.2 (2C), 25.7 (2C), 67.5 (1C), 70.0 (1C), 123.5 (1C), 124.5 (1C), 126.0 (1C), 132.5 (1C), 133.0 (1C), 133.3 (1C), 142.4 (1C), 148.4 (1C), 148.5 (1C). ^1^H-NMR (CDCl_3_ + (PhNH)_2_) δ: 1.37 (s, 6H), 1.48 (s, 6H) 5.86 (s, 1H), 6.68 (d, *J* = 16.5 Hz, 1H), 6.82 (d, *J* = 16.5 Hz, 1H), 7.76 (d, *J* = 7.8 Hz, 1H), 8.54 (d, *J* = 4.4 Hz, 1H), 8.71 (s, 1H). 1H is overlapped with diphenyl hydrazine peaks. IR: 3042, 3017, 2974, 2928, 2868, 1633, 1566 cm^−1^. MS (EI): *m*/*z* (%): 243 (M^+^, 20), 228 (42), 213 (100), 198 (75), 125 (37), 93 (61).

#### 3.12.4. (*E*)-(1-Oxyl-2,2,5,5-tetramethyl-3-(2-(thiophen-2-yl)vinyl)-2,5-dihydro-1*H*-pyrrol Radical (**20d**)

Purified by flash column chromatography (eluent: hexane/Et_2_O, 2:1) to give brown crystals; mp 75–77 °C (635 mg, 51%); TLC (CHCl_3_/Et_2_O, 2:1): R*_f_* = 0.5. ^13^C-NMR ((CDCl_3_ + (PhNH)_2_) δ: 25.3 (2C), 25.9 (2C), 67.5 (1C), 70.0 (1C), 122.1 (1C), 124.4 (1C), 125.9 (1C), 127.6 (1C), 132.1 (1C), 142.4 (1C), 143.0 (1C). ^1^H-NMR (CDCl_3_ + (PhNH)_2_) δ: 1.40 (s, 6H), 1.51 (s, 6H) 5.81 (s, 1H), 6.52 (d, *J* = 16.2 Hz, 1H), 7.03 (d, *J* = 16.2 Hz, 1H), 7.08–7.26 (m, 3H). IR: 3101, 3059, 3037, 2979, 2930, 2862. 1624 cm^−1^. MS (EI): *m*/*z* (%): 248 (M^+^, 16), 233 (24), 218 (100), 203 (59), 175 (48), 44 (73).

### 3.13. (R,S)-1-Oxyl-3-phenethyl-2,2,5,5-tetramethylpyrrolidine Radical *(**21**)*

A solution of compound **20b** (485 mg, 2.0 mmol) in anhydr. EtOH (75 mL) was subjected to continuous flow hydrogenation by a H-Cube Mini Plus apparatus with a 10% Pd/C catalyst cartridge. After consumption of the starting material (monitored by TLC and the content of the receiving flask), the solvent was evaporated, the residue was dissolved in CHCl_3_ (25 mL), MnO_2_ (17.4 mg, 0.2 mmol) was added, and the mixture was bubbled with O_2_ for 30 min., followed by filtration through a Celite pad. After rinsing the pad with CHCl_3_ (10 mL), the filtrate was evaporated and the crude product was purified by flash column chromatography (eluent: hexane/Et_2_O, 2:1) to give an orange powder; mp 60–62 °C (367 mg, 74%); TLC (hexane/Et_2_O, 2:1): R*_f_* = 0.35. ^13^C-NMR ((CDCl_3_ + (PhNH)_2_) δ: 17.2 (1C), 26.6 (1C), 27.2 (1C), 29.9 (1C), 32.4(1C), 34.7(1C), 43.0 (1C), 43.1 (1C), 61.4 (1C), 66.5(1C), 125.9 (1C), 128.4 (2C), 128.5 (2C), 142.6 (1C). ^1^H-NMR (CDCl_3_ + (PhNH)_2_) δ: 1.10 (s, 3H), 1.29 (s, 3H), 1.33 (s, 3H), 1.36 (s, 3H), 1.54–1.59 (m, 2H), 1.86–1.89 m (2H), 1.98–2.02 (m 1H), 2.61–2.67 (m, 1H), 2.77–2.82 (m, 1H), 7.42–7.45 (m, 3H). 2H are overlapped with peaks of with diphenyl hydrazine. IR: 3066, 3025, 2965, 2917, 2879, 2857, 1602 cm^−1^. MS (EI): *m*/*z* (%): 246 (M^+^, 43), 216 (26), 117 (19), 91 (100).

### 3.14. 6-Diphenyl-2-Oxyl-1,1,3,3-tetramethylisoindoline Radical *(**22**)*

To a suspension of oil-free NaH (144 mg, 6.0 mmol) in anhydrous toluene (10 mL), a solution of compound **4** (1.32 g, 3.0 mmol) in anhydrous toluene (10 mL) was added dropwise at 0 °C under N_2_. After 30 min, a solution of freshly distilled benzaldehyde (848 mg, 8.0 mmol) in toluene (10 mL) was added dropwise at 0 °C. The mixture was refluxed for 3 h. After cooling, sat. aq. NH_4_Cl solution (5 mL) and Et_2_O (30 mL) were added to the mixture and stirred for 10 min. The organic phase was separated, dried (MgSO_4_), filtered, and evaporated. The residue was dissolved in toluene (20 mL), followed by the addition of 2,3-dichloro-5,6-dicyano-1,4-benzoquinone (DDQ, 681 mg, 3.0 mmol), and the mixture was refluxed with stirring for 2 h. After cooling, the solvent was evaporated, and the residue was partitioned between 10% aq. Na_2_CO_3_ solution (25 mL) and EtOAc (50 mL). The organic phase was separated, dried (MgSO_4_), filtered, and evaporated, and the crude product was purified by flash column chromatography (eluent: hexane/Et_2_O, 2:1) to give a beige powder; mp 213–216 °C (500 mg, 48%); TLC (hexane/Et_2_O, 2:1): R*_f_* = 0.40. ^13^C-NMR of *O*-acetyl ((CDCl_3_ + (PhNH)_2_) δ: 19.3 (1C), 25.3 (1C), 28.9 (4C), 68.3 (2C), 123.7 (2C), 126.5 (2C) 127.8 (4C), 129.9 (2C), 140.4 (2C), 141.6 (2C), 143.3 (2C), 171.7 (2C). ^1^H-NMR of *O*-acetyl (CDCl_3_ + (PhNH)_2_) δ: 1.53 (s, 6H), 1.59 (s, 6H) 2.28 (s, 3H), 7.17–7.28 (m, 12H). IR: 3057, 3026, 2979, 2925, 2853, 1601 cm^−1^. MS (EI): *m*/*z* (%): 342 (M^+^, 1), 312 (100), 297 (21), 141 (10).

### 3.15. ABTS Scavenging Assay

The measurements were collected on a Specord 40 instrument. ABTS was dissolved in PBS buffer (0.136 M NaCl, 0.0027 M KCl, 0.01 M Na_2_HPO_4_, 0.00176 M KH_2_PO_4_) to a 7.0 mM concentration. ABTS radical cations (ABTS•+) were produced by reacting the ABTS stock solution with potassium persulfate at a final concentration of 2.45 mM and allowing the mixture to stand in the dark at room temperature for 16 h before use. For study of the compounds, the ABTS•+ solution was diluted with water to an absorbance of 0.70(±0.02) at 734 nm and equilibrated at 37 °C. Stock solutions of new compounds and Trolox in dimethylsulfoxide (DMSO) were added to the diluted ABTS•+solution in final concentrations of 12.5, 10, 7.5, and 2.5 µM. After addition, the mixtures were incubated for 6 min at 37 °C before measuring their absorbance at 734 nm. All determinations were repeated three times. The percentage inhibition of absorbance at 734 nm is calculated with the usual formula: (A_0_—A_antioxidant_)/A_0_, where A_0_ is the absorbance of the diluted ABTS•+ solution. The concentration–response curves of new compounds were compared with the curve of Trolox.

## 4. Conclusions

In conclusion, the Arbusov, Pudovik, Perkow and HWE reactions were adopted to access paramagnetic allylic-, vinyl-, acetylene- and α-hydroxyphosphonates or vinyl phosphates, giving the desired products with moderate to good yields. α-hydroxyphosphonates could be further transformed by oxidation, substitution or elimination reactions. We demonstrated that allylic phosphonates are good building blocks in olefination reactions for the introduction of pyrroline nitroxide rings in various scaffolds. Additionally, paramagnetic saturated α-hydroxyphosphonates exhibited remarkable antioxidant (proton and electron donor) activity against the ABTS•+ radical. Further synthetic, biological and biophysical applications of the newly synthesized nitroxide phosphonates are in progress.

## Figures and Tables

**Table 1:** TEAC activity of phosphonates.

Compound	2a	2c	8a	8b	10a	10c	TEMPOL
TEAC ^1^	0.13 ± 0.01	0.55 ± 0.03	0.96 ± 0.05	0.93 ± 0.04	0.35 ± 0.01	0.51 ± 0.02	1.27 ± 0.04

^1^ Based on *n* = 3 parallel measurements.

## Supplementary Materials

The following are available online, ^31^P-NMR, ^1^H-NMR and ^13^C-NMR spectra of reduced in situ compounds **2a, 2b, 2c, 4, 7, 8a, 8b, 8c, 10a, 10b, 10c, 11, 12, 13, 16, 17, 19, 20a, 20b, 20c, 20d, 21, 22** and structure of tempol and trolox.
