# Supplementary material for: Syntheses and Reactions of Pyrroline, Piperidine Nitroxide Phosphonates
Source: Molecules. 2020 May 22;25(10):2430. doi: 10.3390/molecules25102430 (PMC7287729; doi:10.3390/molecules25102430)
Supplement: Supplementary file 1 [file molecules-25-02430-s001.pdf]

Supplementary Material

Syntheses and reactions of pyrroline, piperidine nitroxide phosphonates

Mostafa Isbera [1], Balázs Bognár [1], József Jekő [2], Cecilia Sár [1], Kálmán Hideg [1], and Tamás Kálai [1,*]

[1] Institute of Organic and Medicinal Chemistry, Medical School, University of Pécs, Szigeti st. 12, 7624 Pécs, Hungary; tamas.kalai@aok.pte.hu
[2] Department of Chemistry, University of Nyíregyháza, Sóstói st. 31/B, 4440 Nyíregyháza, Hungary
* Correspondence: tamas.kalai@aok.pte.hu; Tel.: +36-72-536-220 (T. K.)

Contents

^{31}P NMR, ^{1}H NMR and ^{13}C NMR spectra of compounds **2a, 2b, 2c, 4, 7, 8a, 8b, 8c, 10a, 10b, 10c, 11, 12, 13, 16, 17, 19, 20a, 20b, 20c, 20d, 21, 22** **p**2 - p31

Structure of Tempol and Trolox p32

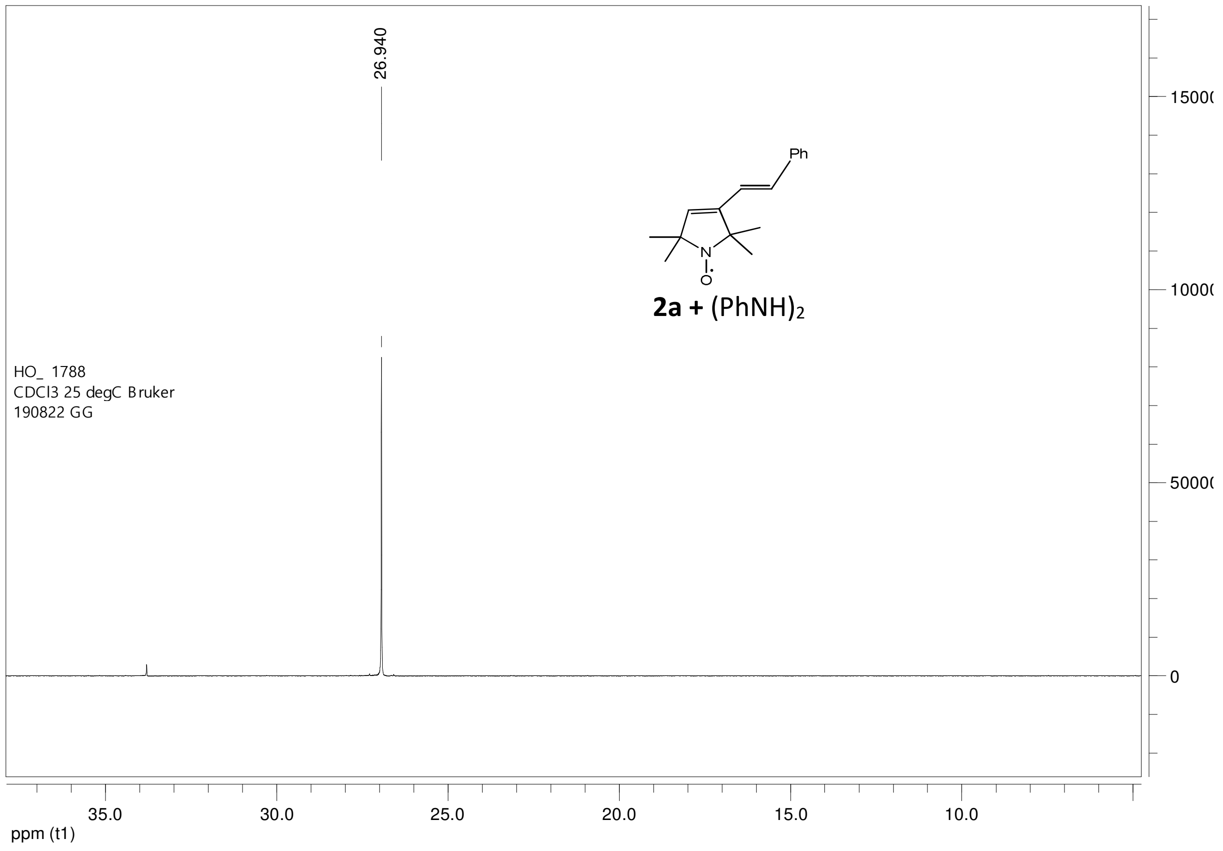
26.940
Ph
N
O
2a + (PhNH)2
HO_ 1788
CDCl3 25 degC Bruker
190822 GG
15000
10000
5000
0
35.0
30.0
25.0
20.0
15.0
10.0
ppm (t1)

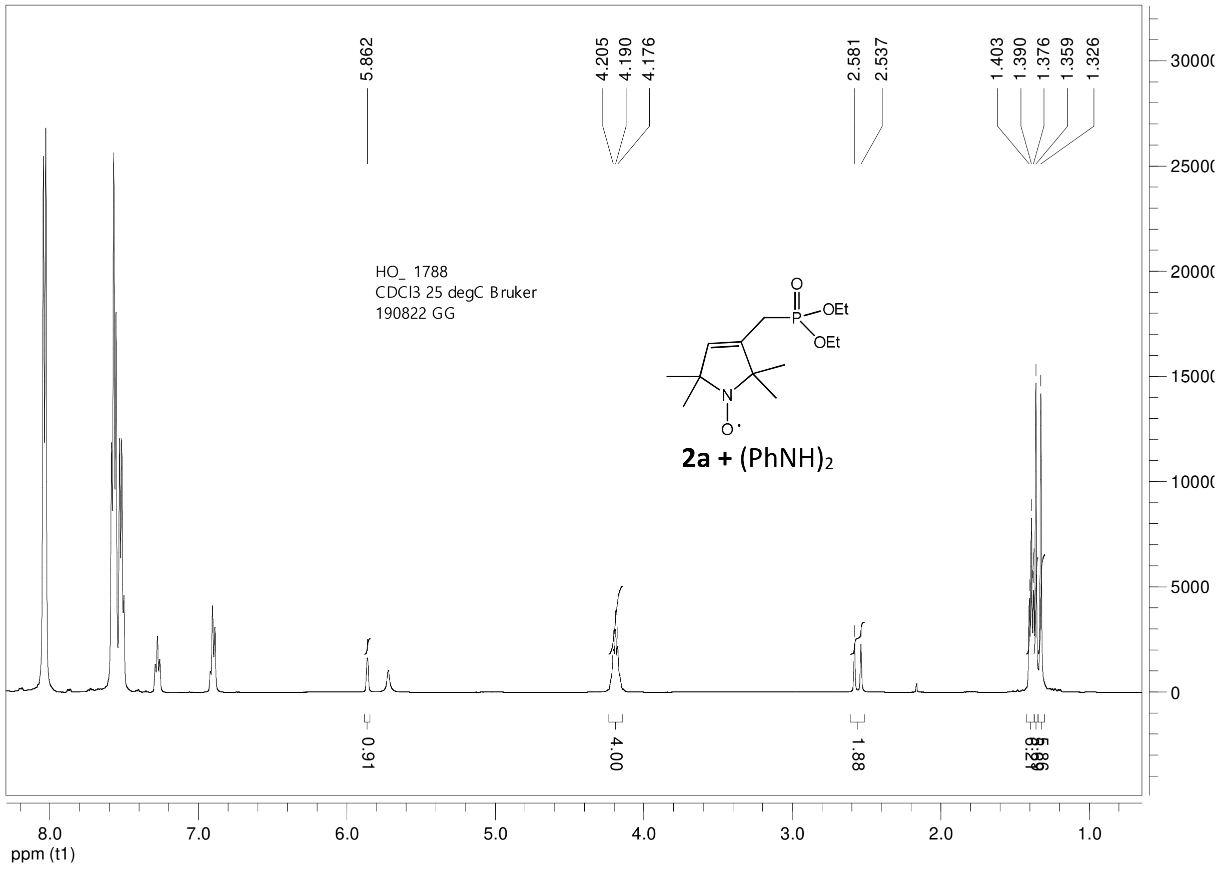
5.862
4.205
4.190
4.176
2.581
2.537
1.403
1.390
1.376
1.359
1.326
HO_ 1788
CDCl3 25 degC Bruker
190822 GG
O
P
OEt
OEt
N
O
2a + (PhNH)2
0.91
4.00
1.88
30000
25000
20000
15000
10000
5000
0
8.0
7.0
6.0
5.0
4.0
3.0
2.0
1.0
ppm (t1)

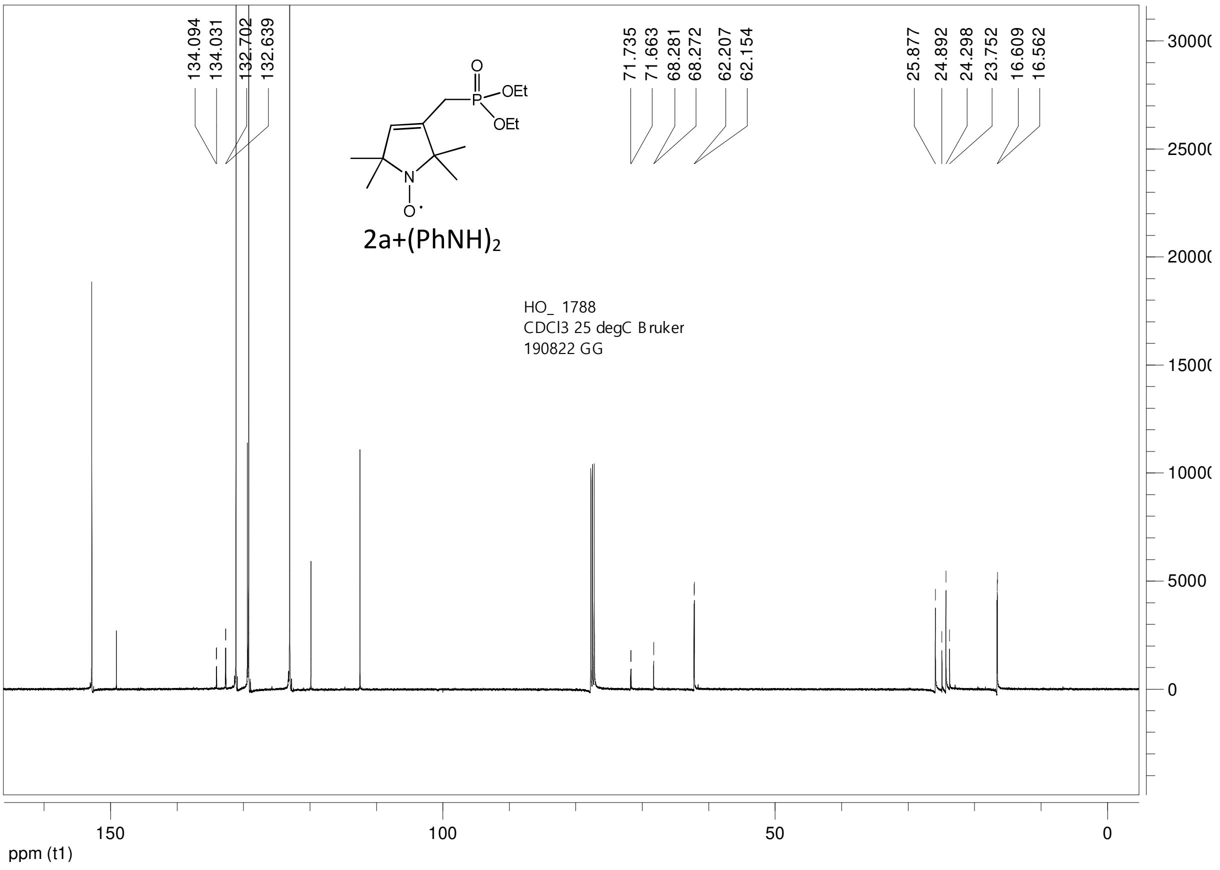
134.094
134.031
132.702
132.639
71.735
71.663
68.281
68.272
62.207
62.154
25.877
24.892
24.298
23.752
16.609
16.562
O
P
OEt
OEt
N
O·
2a+(PhNH)2
HO_ 1788
CDCl3 25 degC Bruker
190822 GG
150
100
50
0
ppm (t1)

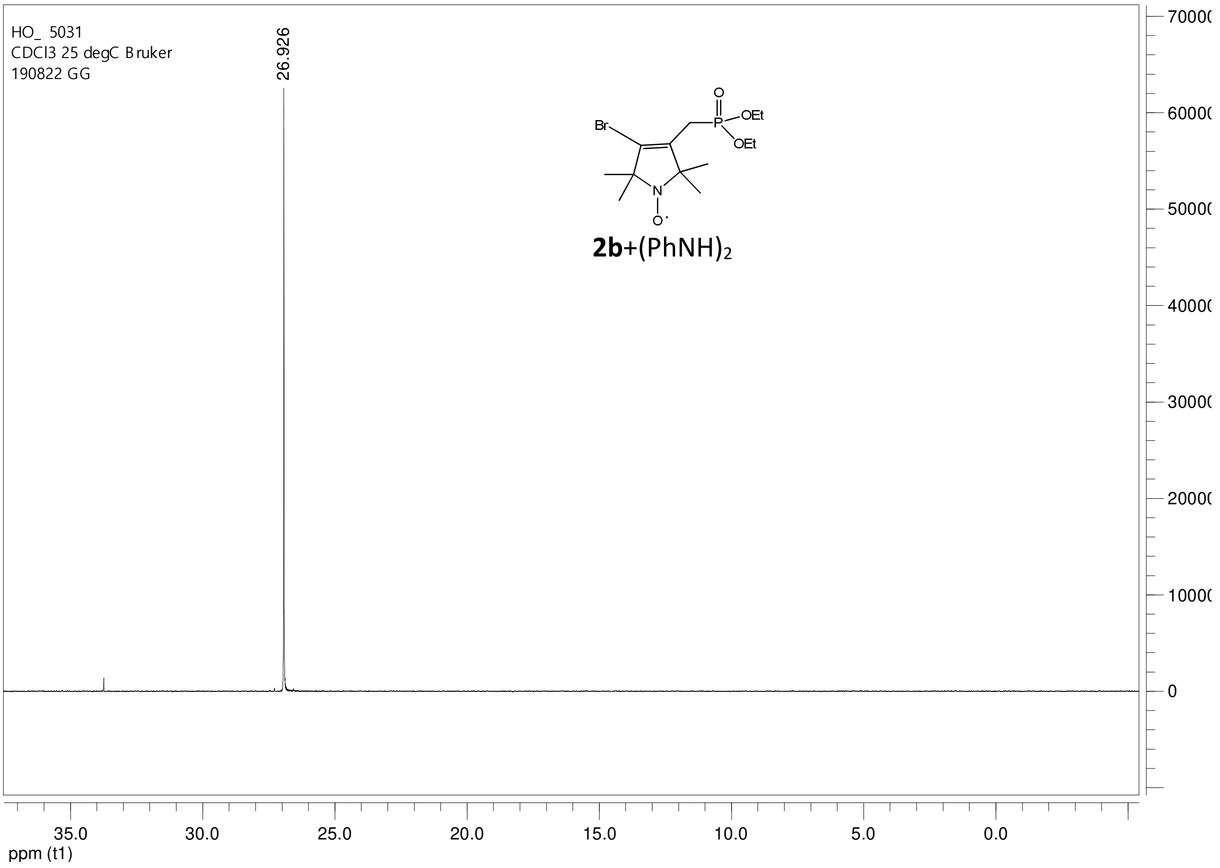
HO_ 5031
CDCl3 25 degC Bruker
190822 GG
26.926
Br
O
P
OEt
OEt
N
O·
2b+(PhNH)2
35.0
30.0
25.0
20.0
15.0
10.0
5.0
0.0
ppm (t1)

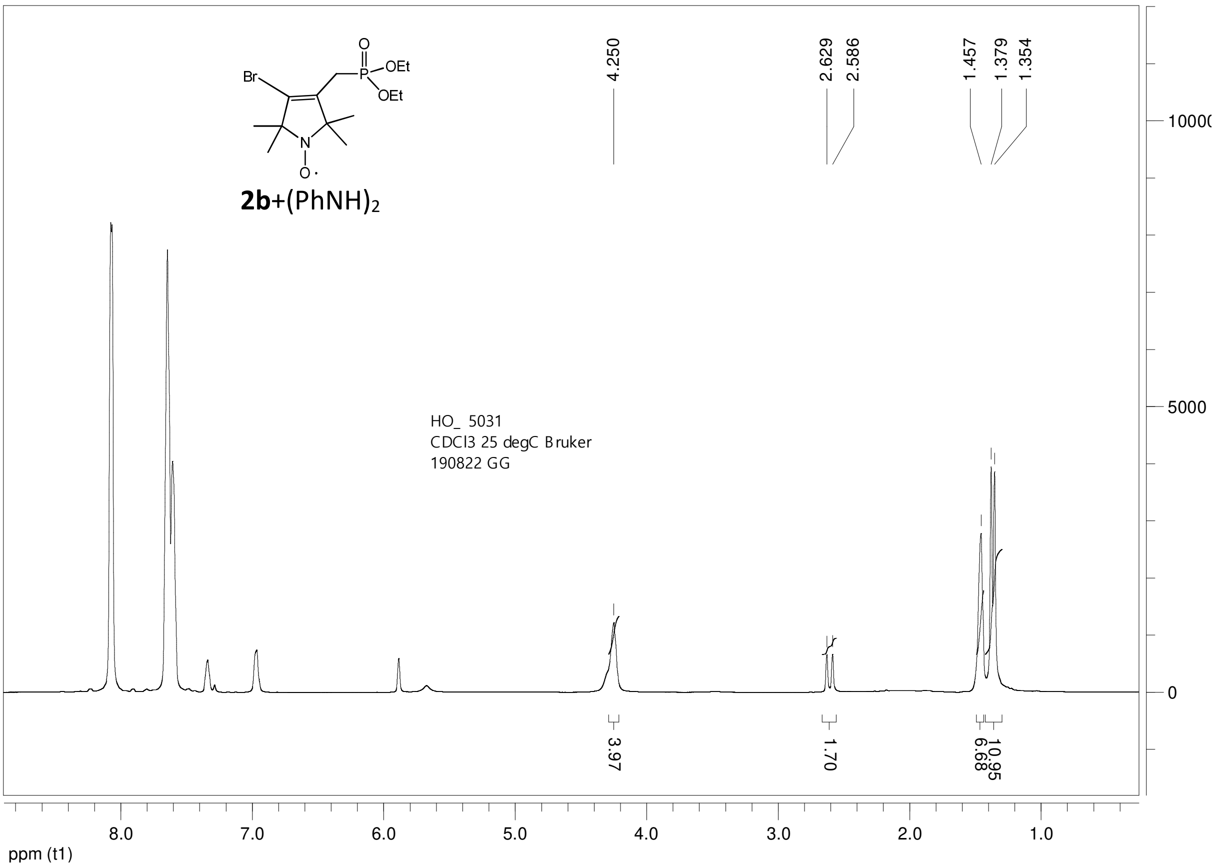

Br
O
P
OEt
OEt
N
O·
2b+(PhNH)$_2$
4.250
2.629
2.586
1.457
1.379
1.354
HO_ 5031
CDCl3 25 degC Bruker
190822 GG
3.97
1.70
6.68
10.95
10000
5000
0
8.0
7.0
6.0
5.0
4.0
3.0
2.0
1.0
ppm (t1)

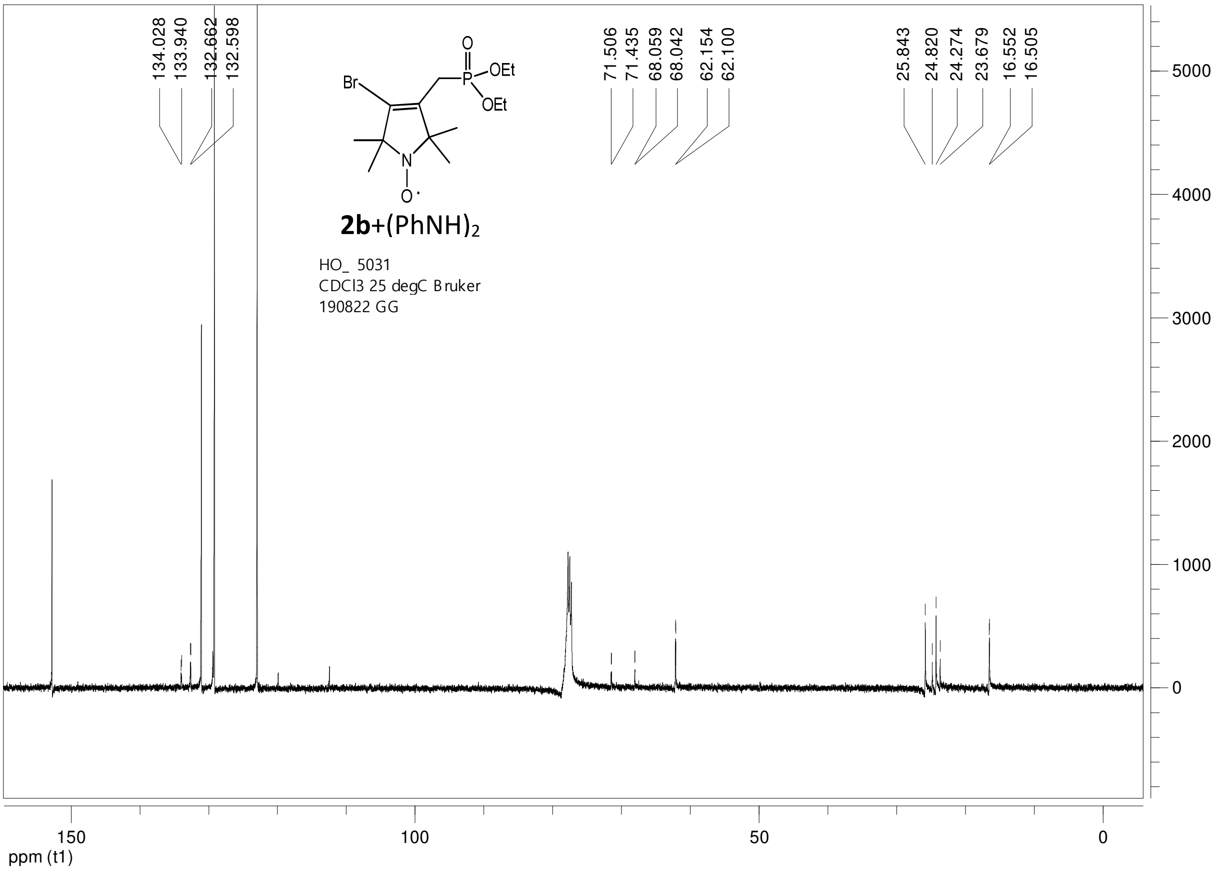

134.028
133.940
132.662
132.598
Br
O
P
OEt
OEt
N
O·
2b+(PhNH)$_2$
HO_ 5031
CDCl3 25 degC Bruker
190822 GG
71.506
71.435
68.059
68.042
62.154
62.100
25.843
24.820
24.274
23.679
16.552
16.505
5000
4000
3000
2000
1000
0
150
100
50
0
ppm (t1)

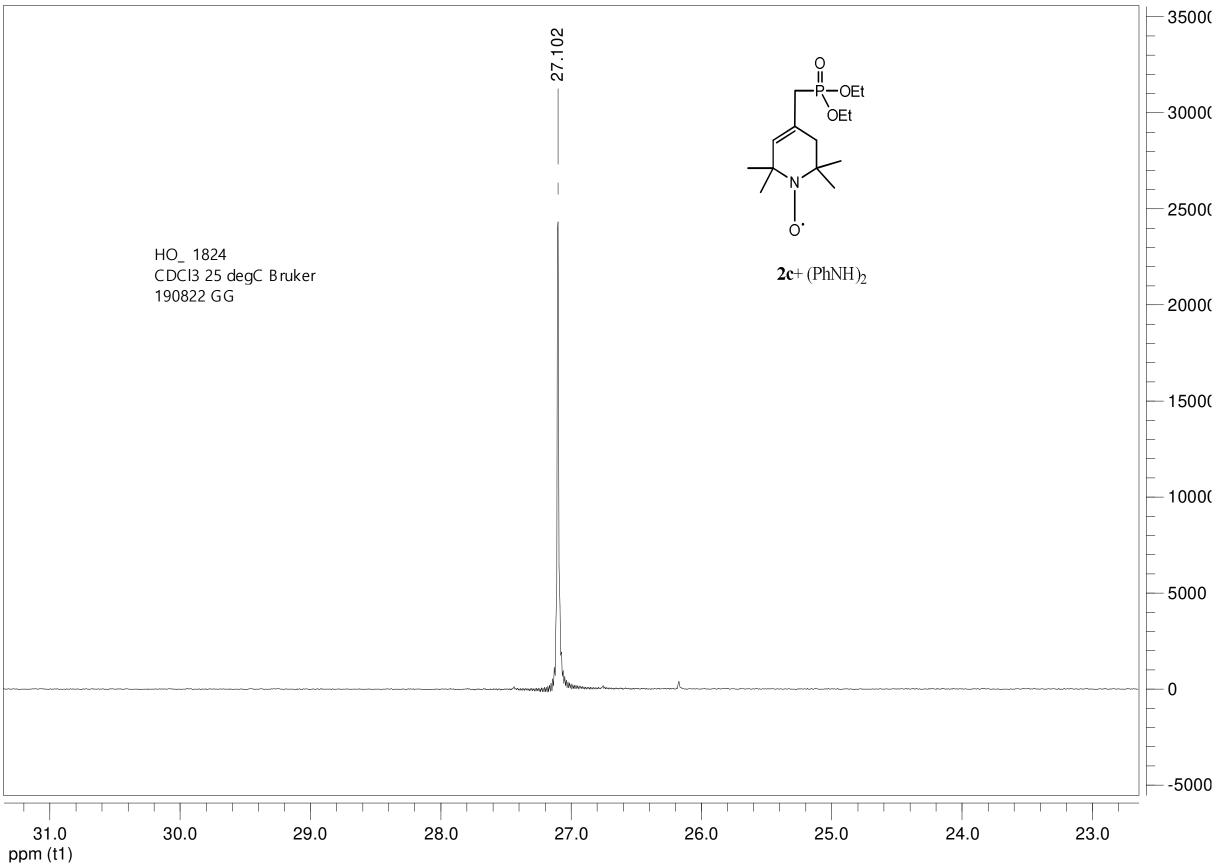
27.102
HO_ 1824
CDCl3 25 degC Bruker
190822 GG
O
P
OEt
OEt
N
O•
2c+ (PhNH)2
35000
30000
25000
20000
15000
10000
5000
0
-5000
31.0
30.0
29.0
28.0
27.0
26.0
25.0
24.0
23.0
ppm (t1)

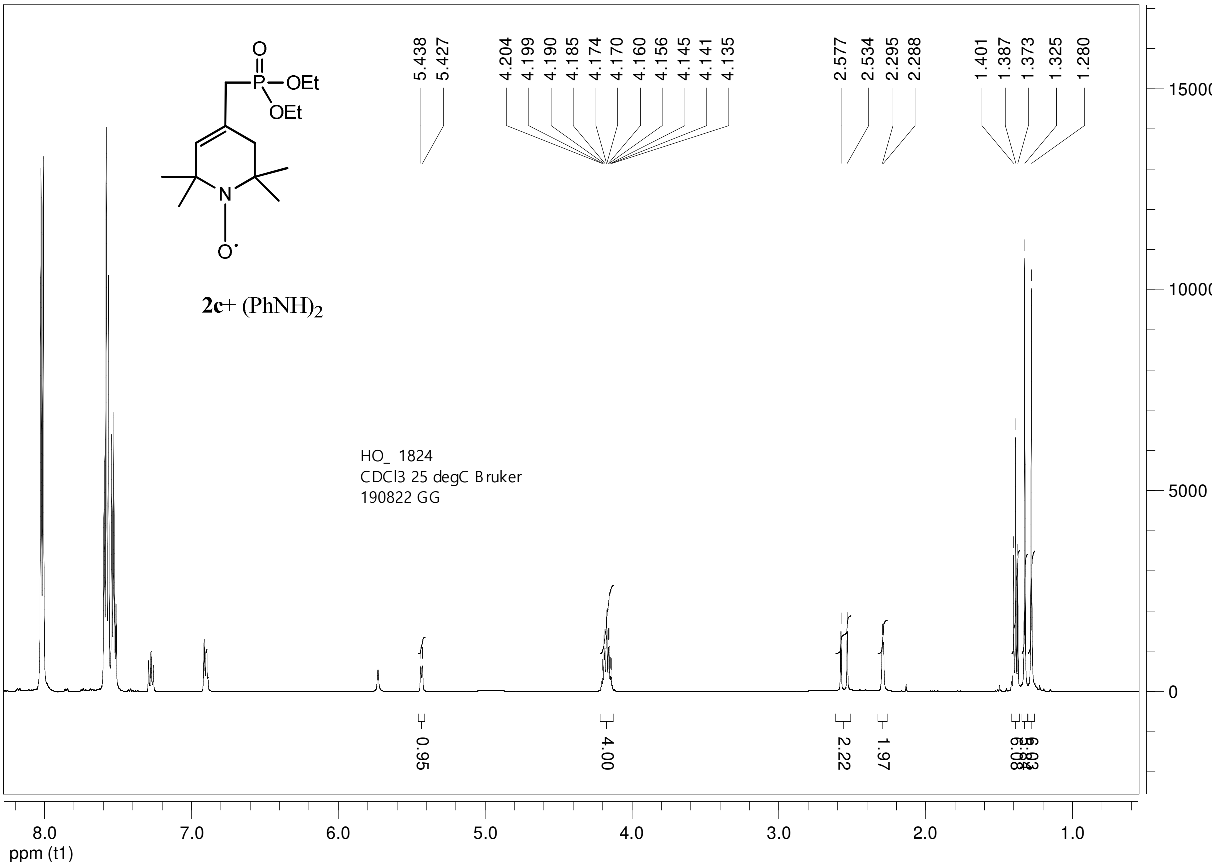
5.438
5.427
4.204
4.199
4.190
4.185
4.174
4.170
4.160
4.156
4.145
4.141
4.135
2.577
2.534
2.295
2.288
1.401
1.387
1.373
1.325
1.280
O
P
OEt
OEt
N
O•
2c+ (PhNH)2
HO_ 1824
CDCl3 25 degC Bruker
190822 GG
0.95
4.00
2.22
1.97
6.08
15000
10000
5000
0
8.0
7.0
6.0
5.0
4.0
3.0
2.0
1.0
ppm (t1)

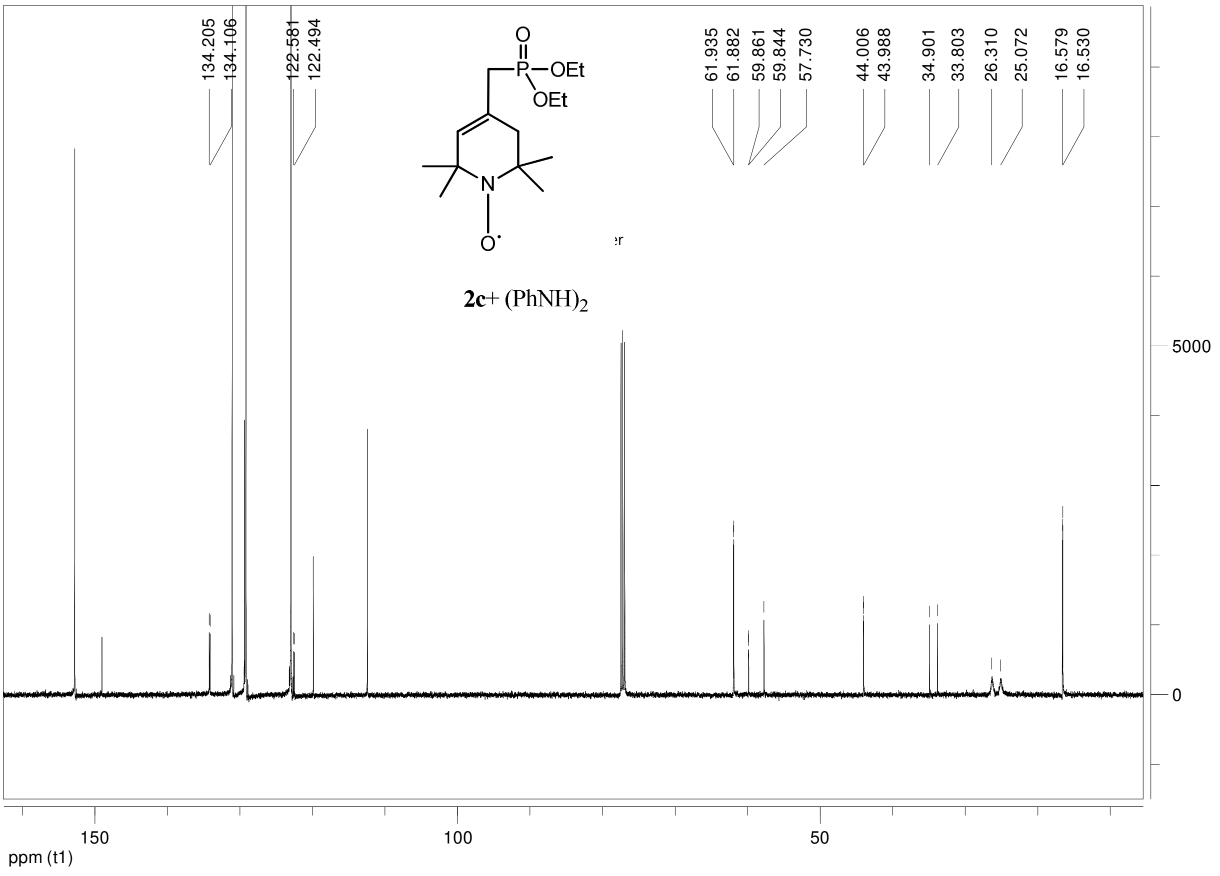
134.205
134.106
122.581
122.494
61.935
61.882
59.861
59.844
57.730
44.006
43.988
34.901
33.803
26.310
25.072
16.579
16.530
2c+ (PhNH)2
5000
0
150
100
50
ppm (t1)

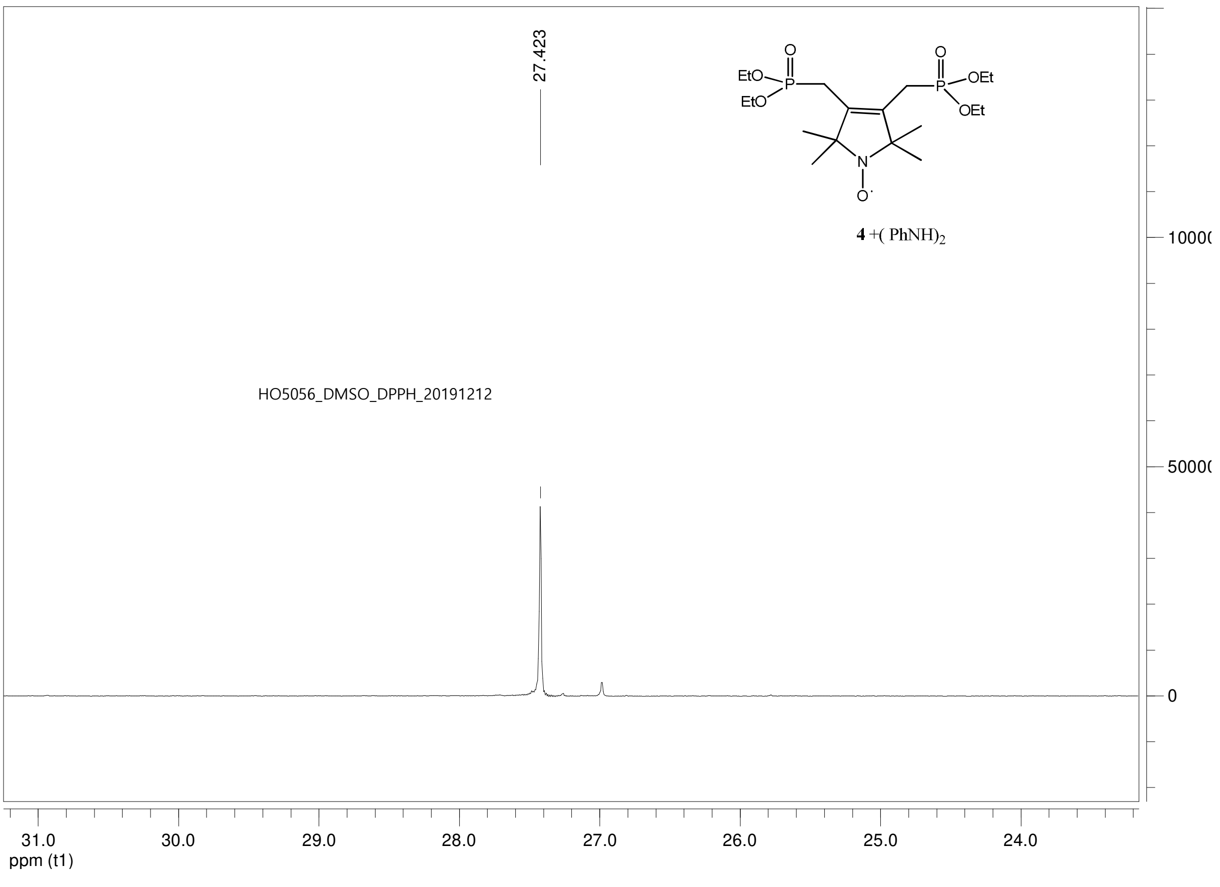
27.423
4 +(PhNH)2
HO5056_DMSO_DPPH_20191212
0
31.0
30.0
29.0
28.0
27.0
26.0
25.0
24.0
ppm (t1)

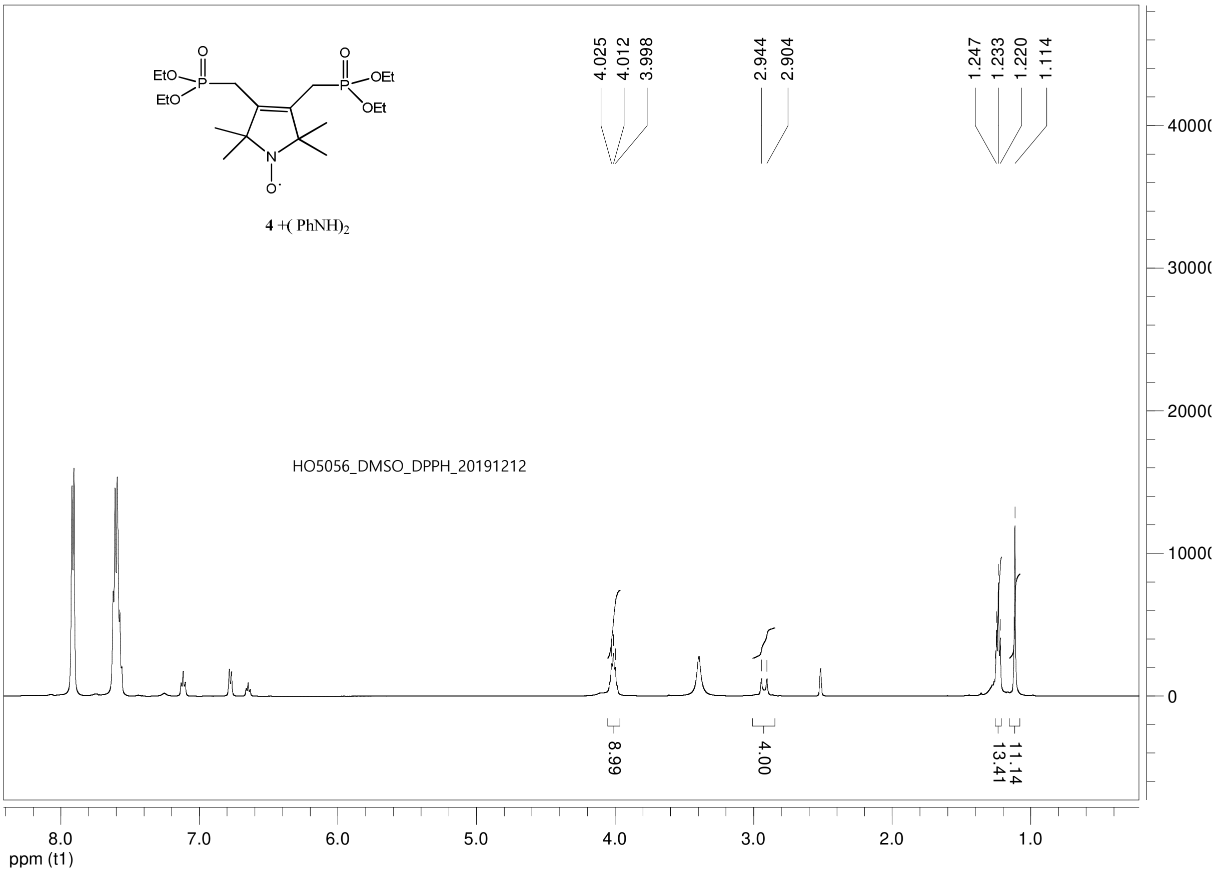

EtO
EtO
P
O
P
O
OEt
OEt
N
O·
4 +(PhNH)2
4.025
4.012
3.998
2.944
2.904
1.247
1.233
1.220
1.114
HO5056_DMSO_DPPH_20191212
8.99
4.00
13.41
11.14
40000
30000
20000
10000
0
8.0
7.0
6.0
5.0
4.0
3.0
2.0
1.0
ppm (t1)

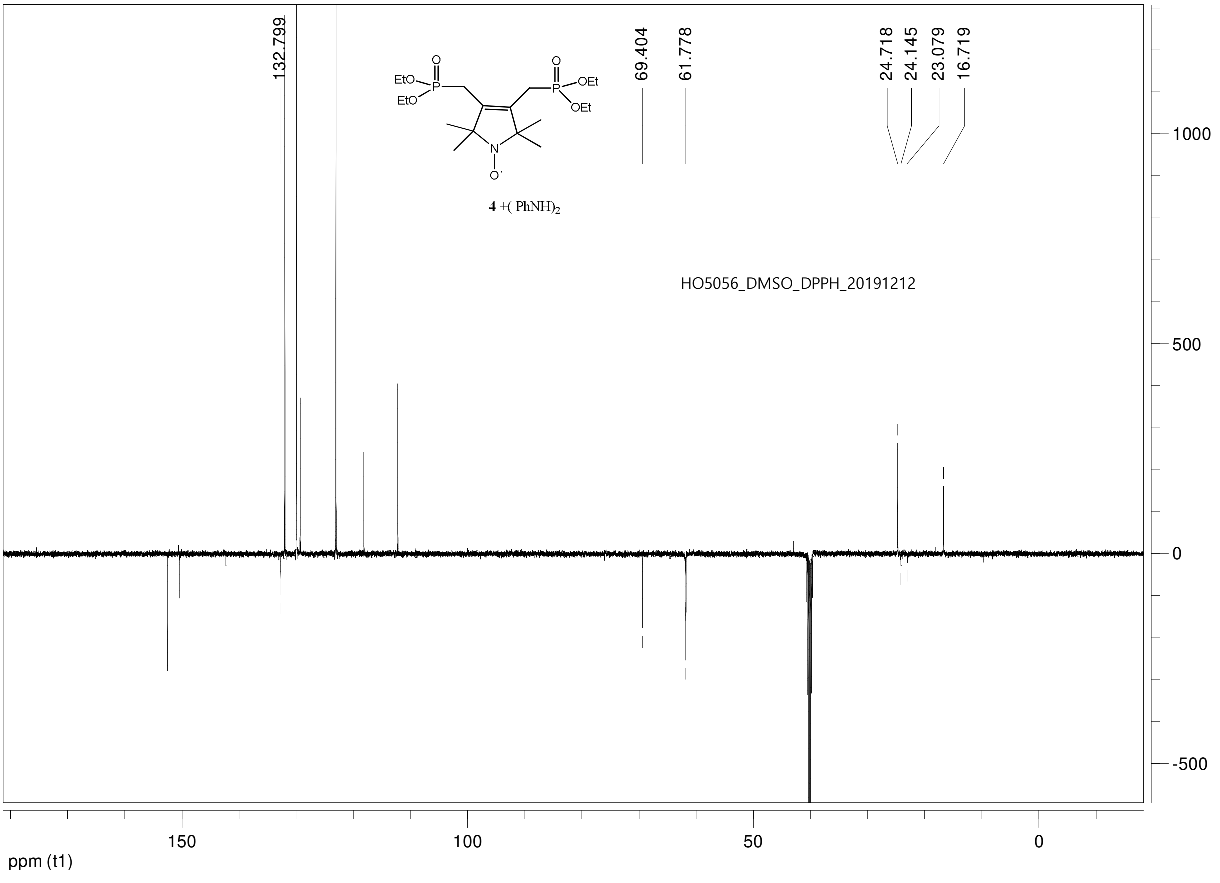

132.799
69.404
61.778
24.718
24.145
23.079
16.719
EtO
EtO
P
O
P
O
OEt
OEt
N
O·
4 +(PhNH)2
HO5056_DMSO_DPPH_20191212
1000
500
0
-500
150
100
50
0
ppm (t1)

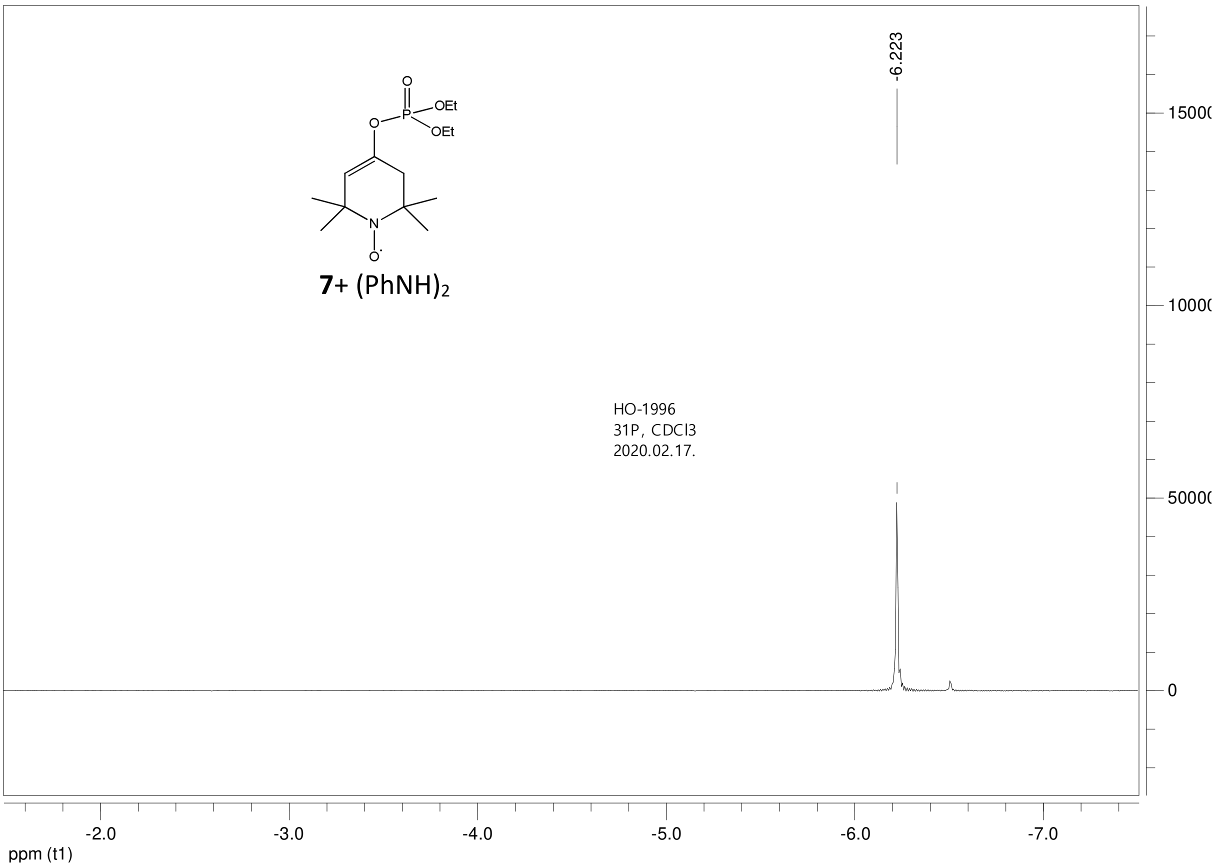
7+ $(PhNH)_2$
OEt
OEt
HO-1996
31P, CDCl3
2020.02.17.
-6.223
ppm (t1)

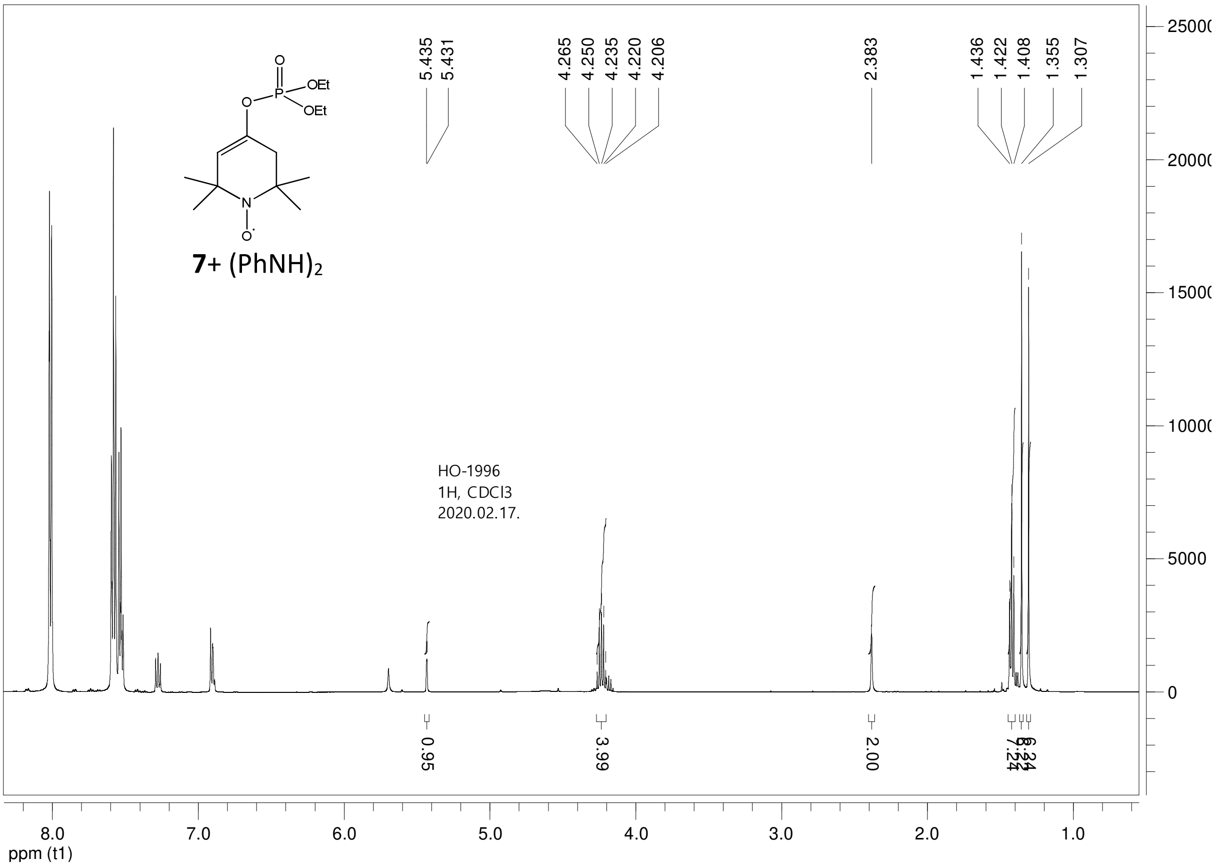
7+ $(PhNH)_2$
OEt
OEt
HO-1996
1H, CDCl3
2020.02.17.
5.435
5.431
4.265
4.250
4.235
4.220
4.206
2.383
1.436
1.422
1.408
1.355
1.307
0.95
3.99
2.00
ppm (t1)

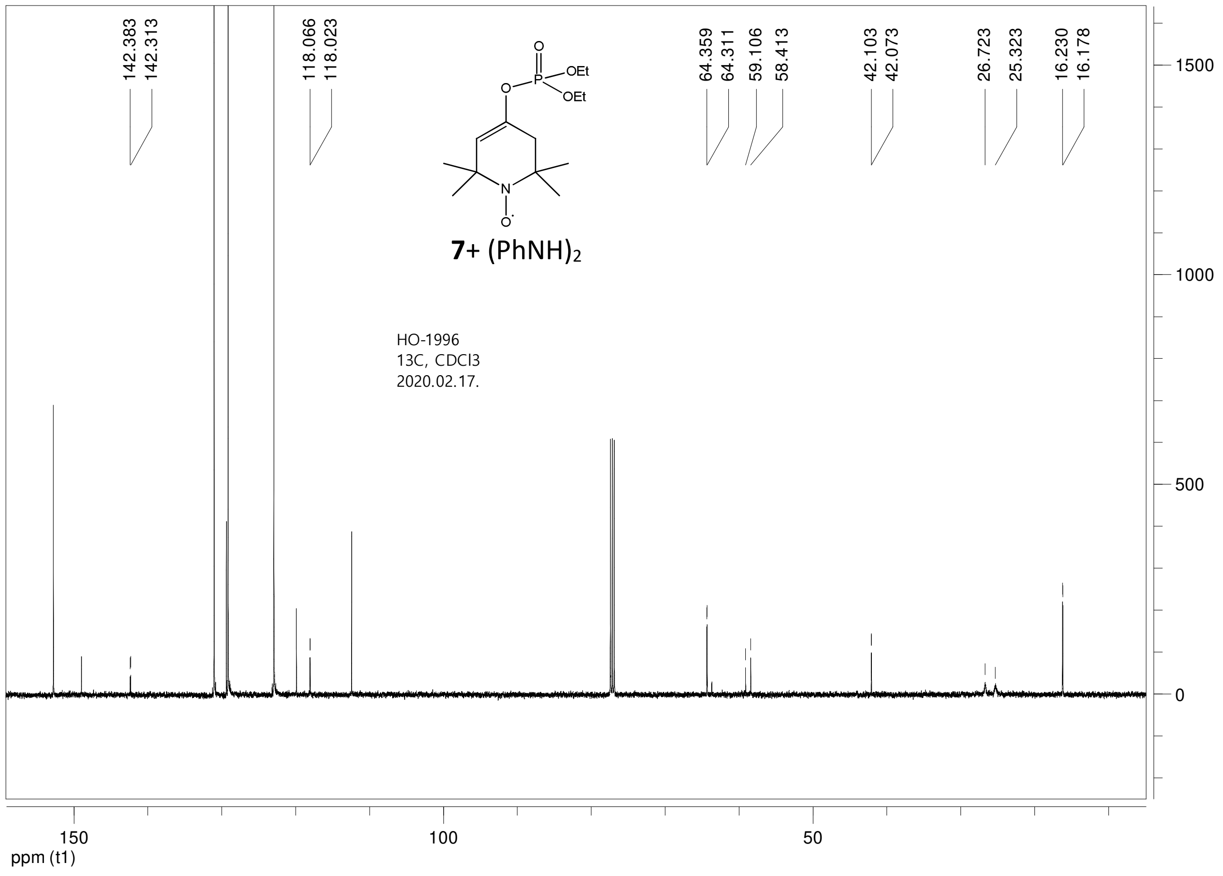
142.383
142.313
118.066
118.023
64.359
64.311
59.106
58.413
42.103
42.073
26.723
25.323
16.230
16.178
O
P
OEt
OEt
N
O
7+ (PhNH)2
HO-1996
13C, CDCl3
2020.02.17.
1500
1000
500
0
150
100
50
ppm (t1)

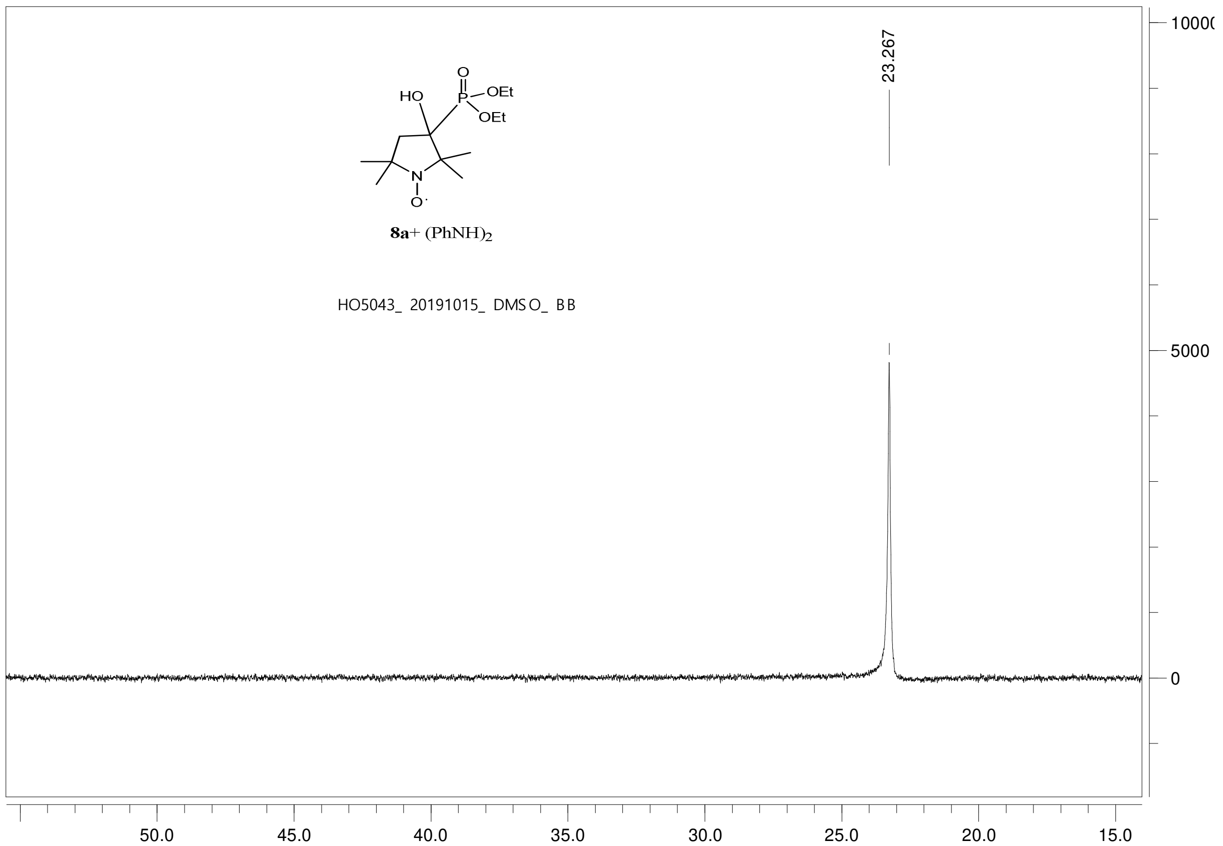
23.267
HO
O
P
OEt
OEt
N
O
8a+ (PhNH)2
HO5043_ 20191015_ DMSO_ BB
10000
5000
0
50.0
45.0
40.0
35.0
30.0
25.0
20.0
15.0
ppm (t1)

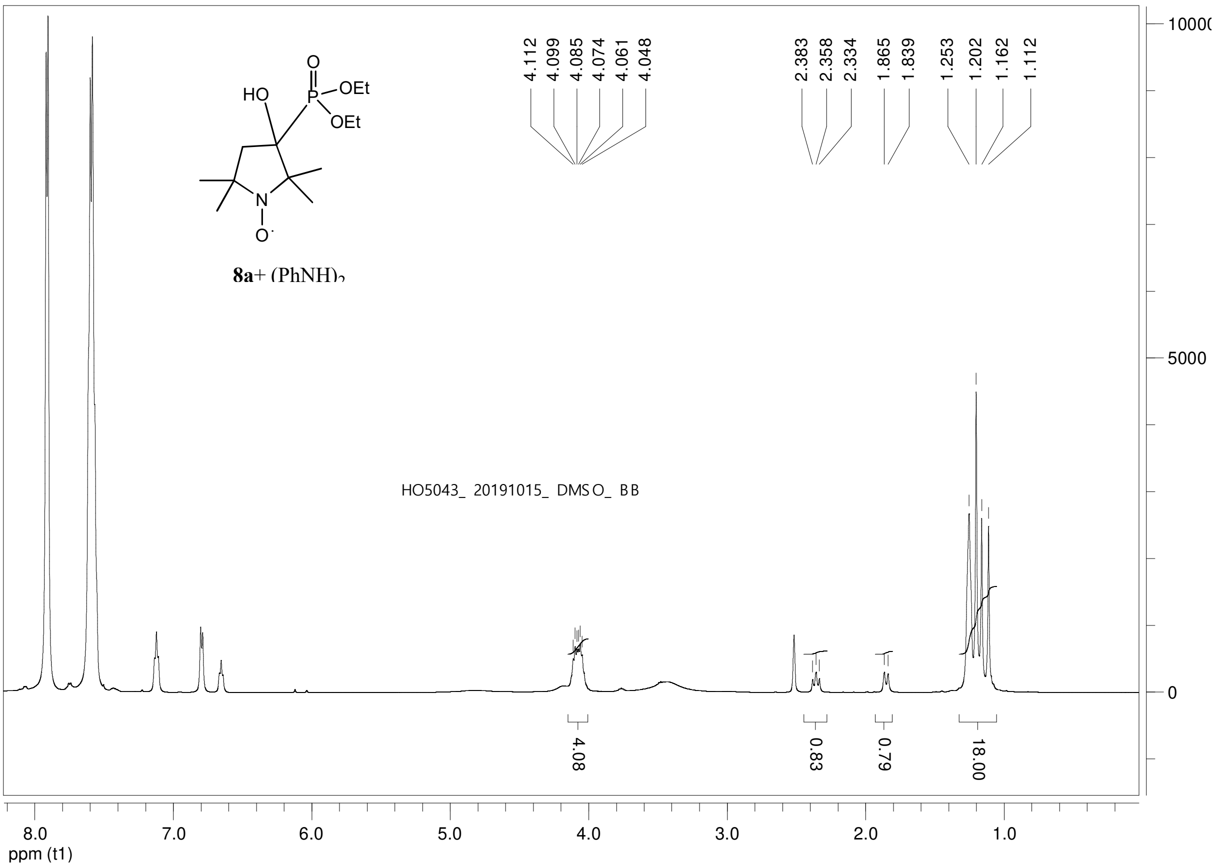

HO
O
P
OEt
OEt
N
O·
8a+ (PhNH)2
4.112
4.099
4.085
4.074
4.061
4.048
2.383
2.358
2.334
1.865
1.839
1.253
1.202
1.162
1.112
HO5043_ 20191015_ DMSO_ BB
4.08
0.83
0.79
18.00
10000
5000
0
8.0
7.0
6.0
5.0
4.0
3.0
2.0
1.0
ppm (t1)

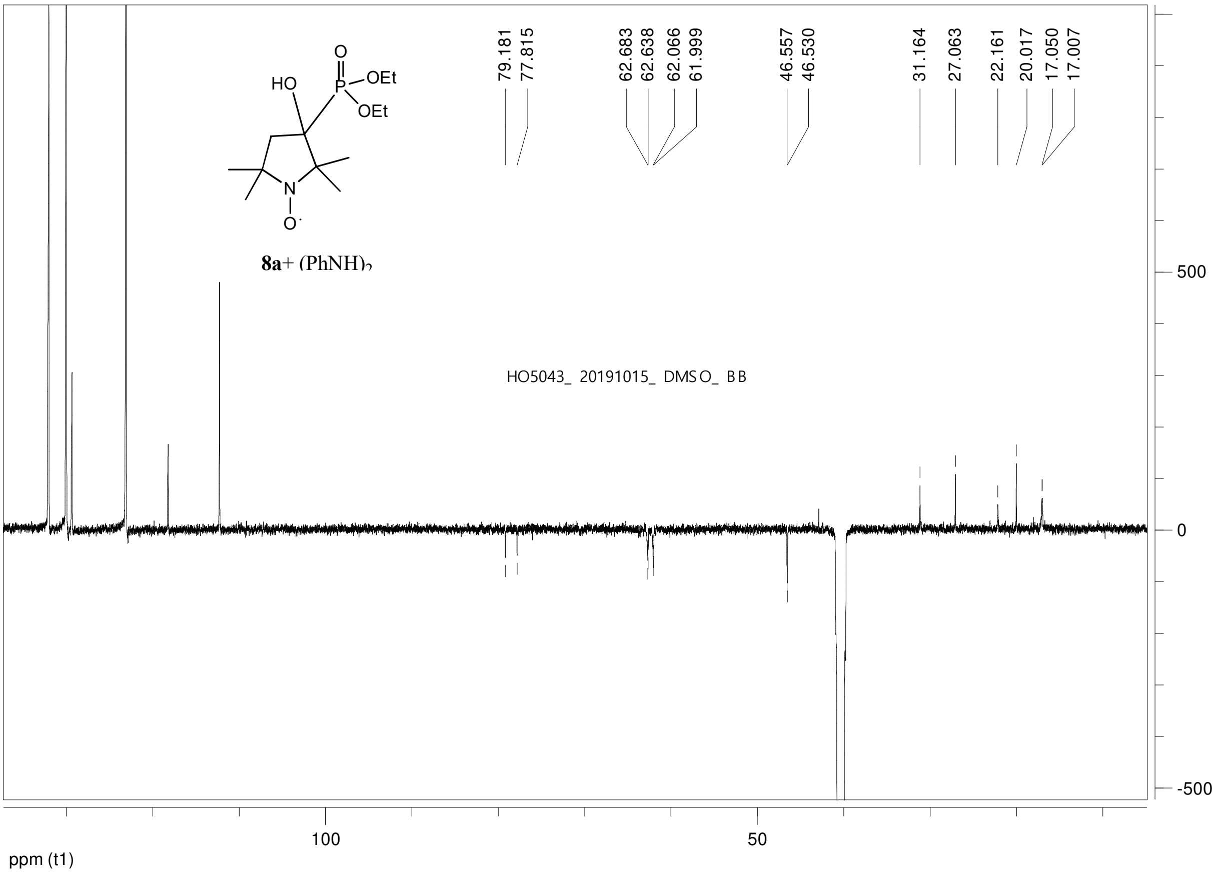

HO
O
P
OEt
OEt
N
O·
8a+ (PhNH)2
79.181
77.815
62.683
62.638
62.066
61.999
46.557
46.530
31.164
27.063
22.161
20.017
17.050
17.007
HO5043_ 20191015_ DMSO_ BB
500
0
-500
100
50
ppm (t1)

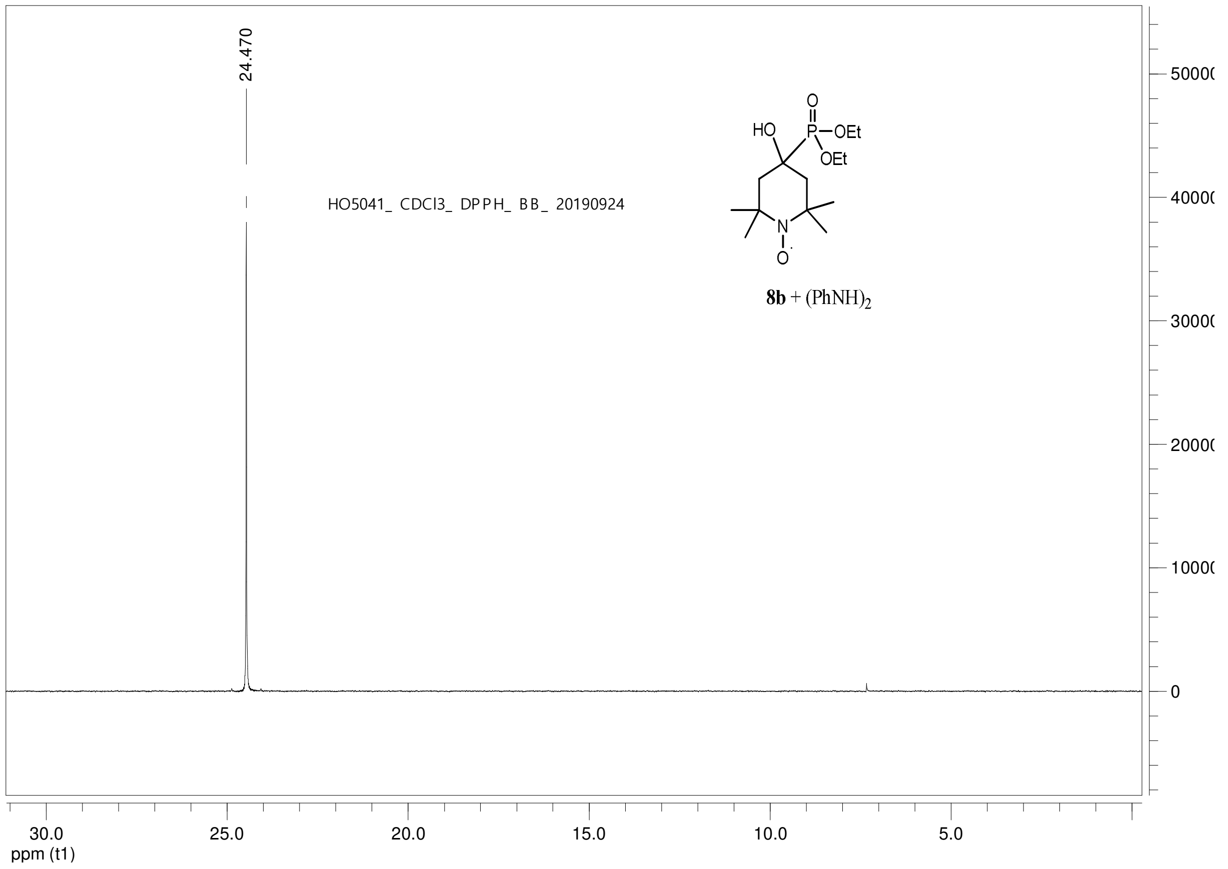
24.470
HO5041_ CDCl3_ DPPH_ BB_ 20190924
HO
P
O
OEt
OEt
N
O
8b + $(PhNH)_2$
50000
40000
30000
20000
10000
0
30.0
25.0
20.0
15.0
10.0
5.0
ppm (t1)

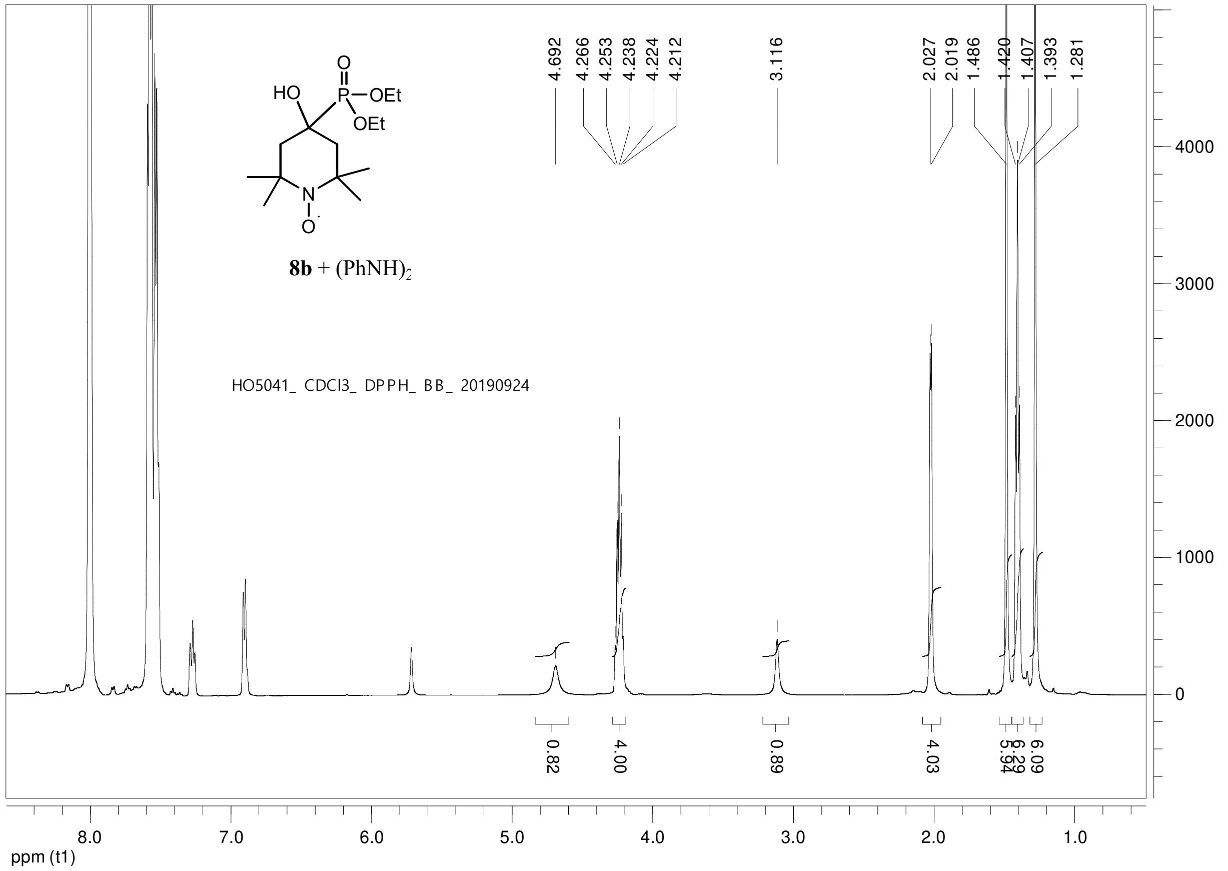
4.692
4.266
4.253
4.238
4.224
4.212
3.116
2.027
2.019
1.486
1.420
1.407
1.393
1.281
HO
P
O
OEt
OEt
N
O
8b + $(PhNH)_2$
HO5041_ CDCl3_ DPPH_ BB_ 20190924
0.82
4.00
0.89
4.03
6.09
4000
3000
2000
1000
0
8.0
7.0
6.0
5.0
4.0
3.0
2.0
1.0
ppm (t1)

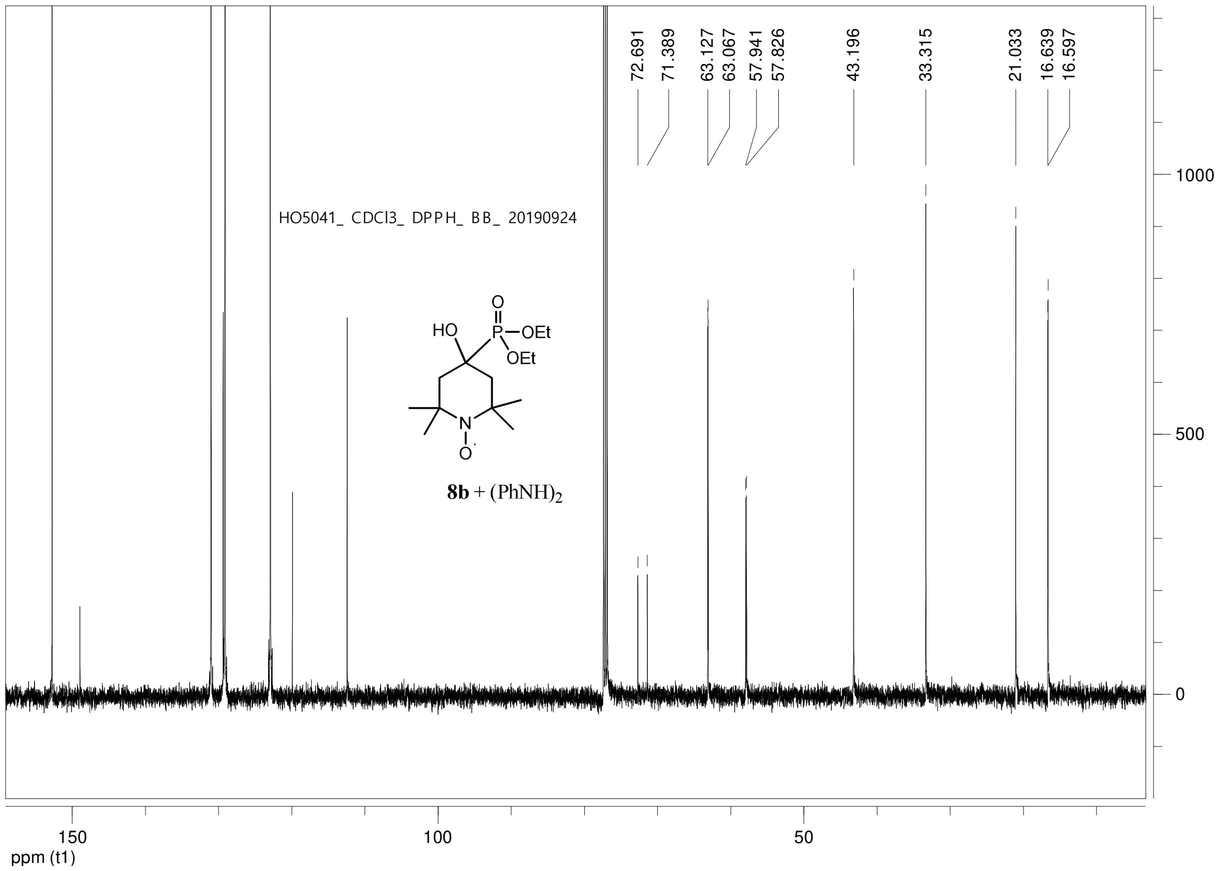
72.691
71.389
63.127
63.067
57.941
57.826
43.196
33.315
21.033
16.639
16.597
HO5041_ CDCl3_ DPPH_ BB_ 20190924
HO
O
P
OEt
OEt
N
O
8b + (PhNH)2
1000
500
0
150
100
50
ppm (t1)

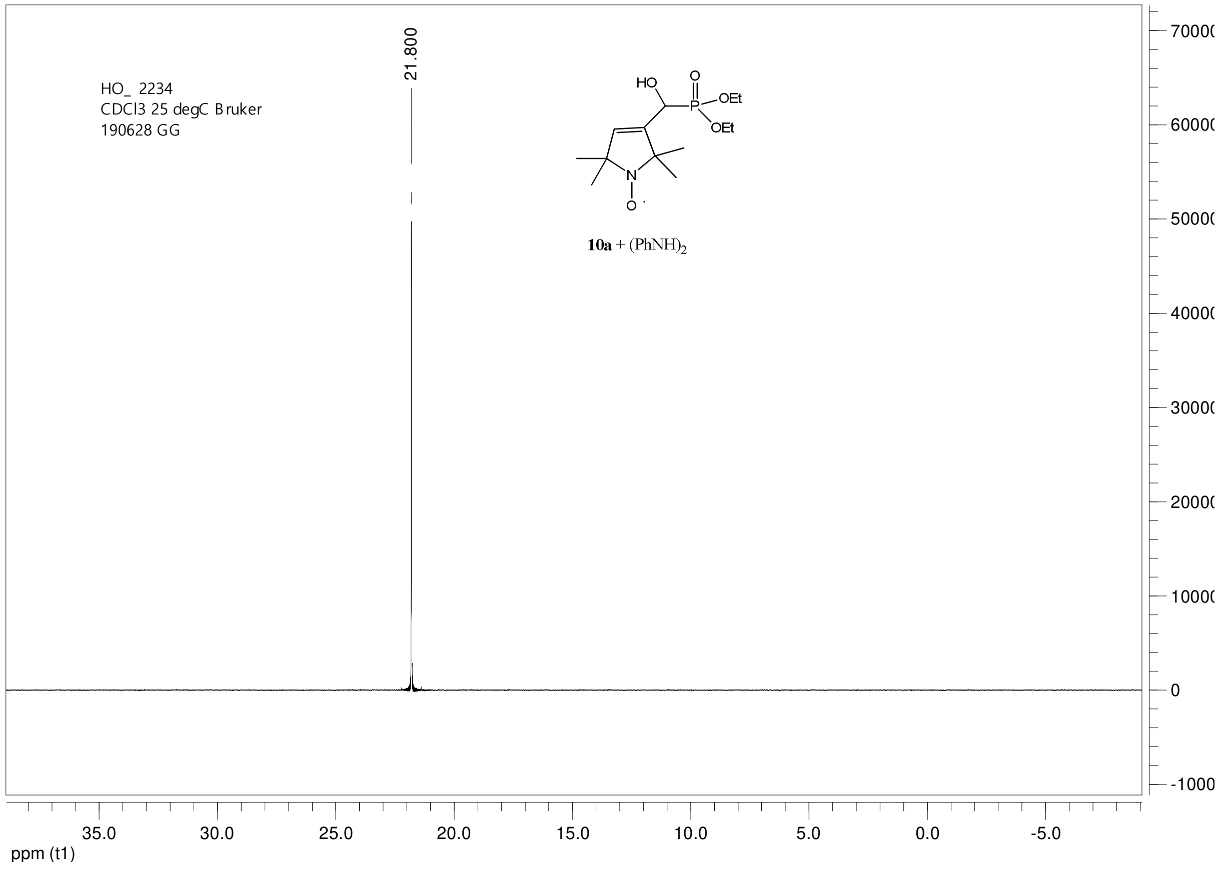
21.800
HO_ 2234
CDCl3 25 degC Bruker
190628 GG
HO
O
P
OEt
OEt
N
O
10a + (PhNH)2
70000
60000
50000
40000
30000
20000
10000
0
-1000
35.0
30.0
25.0
20.0
15.0
10.0
5.0
0.0
-5.0
ppm (t1)

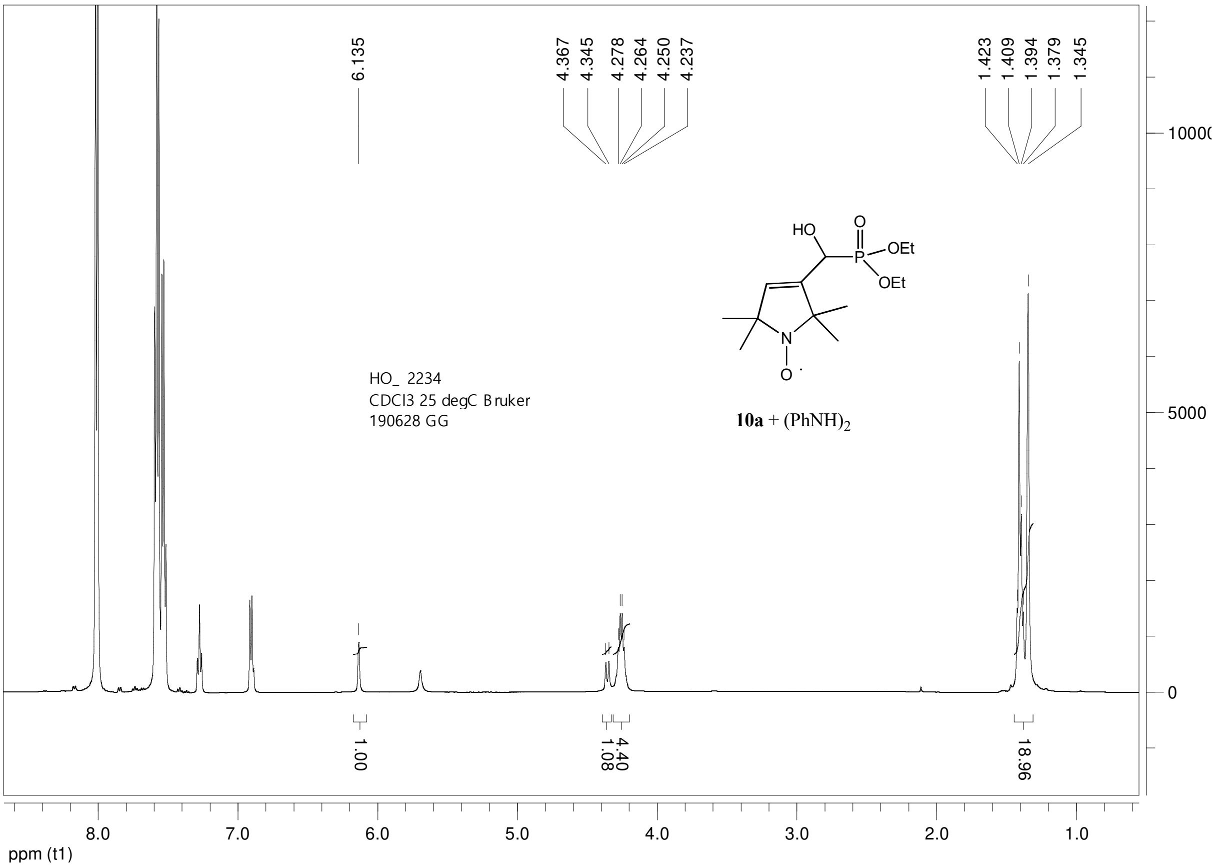
6.135
4.367
4.345
4.278
4.264
4.250
4.237
1.423
1.409
1.394
1.379
1.345
HO
O
P
OEt
OEt
N
O
10a + (PhNH)2
HO_ 2234
CDCl3 25 degC Bruker
190628 GG
1.00
1.08
4.40
18.96
8.0
7.0
6.0
5.0
4.0
3.0
2.0
1.0
ppm (t1)
10000
5000
0

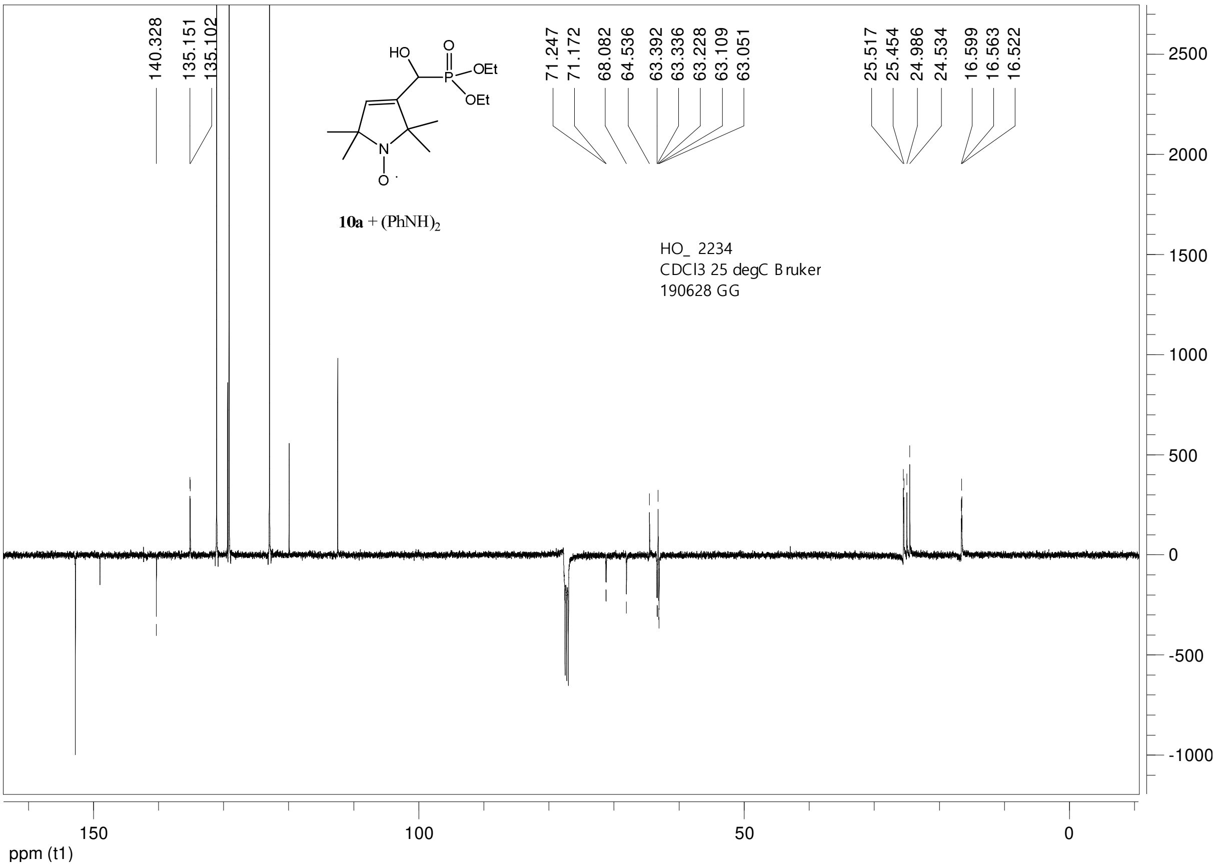
140.328
135.151
135.102
71.247
71.172
68.082
64.536
63.392
63.336
63.228
63.109
63.051
25.517
25.454
24.986
24.534
16.599
16.563
16.522
HO
O
P
OEt
OEt
N
O
10a + (PhNH)2
HO_ 2234
CDCl3 25 degC Bruker
190628 GG
150
100
50
0
ppm (t1)
2500
2000
1500
1000
500
0
-500
-1000

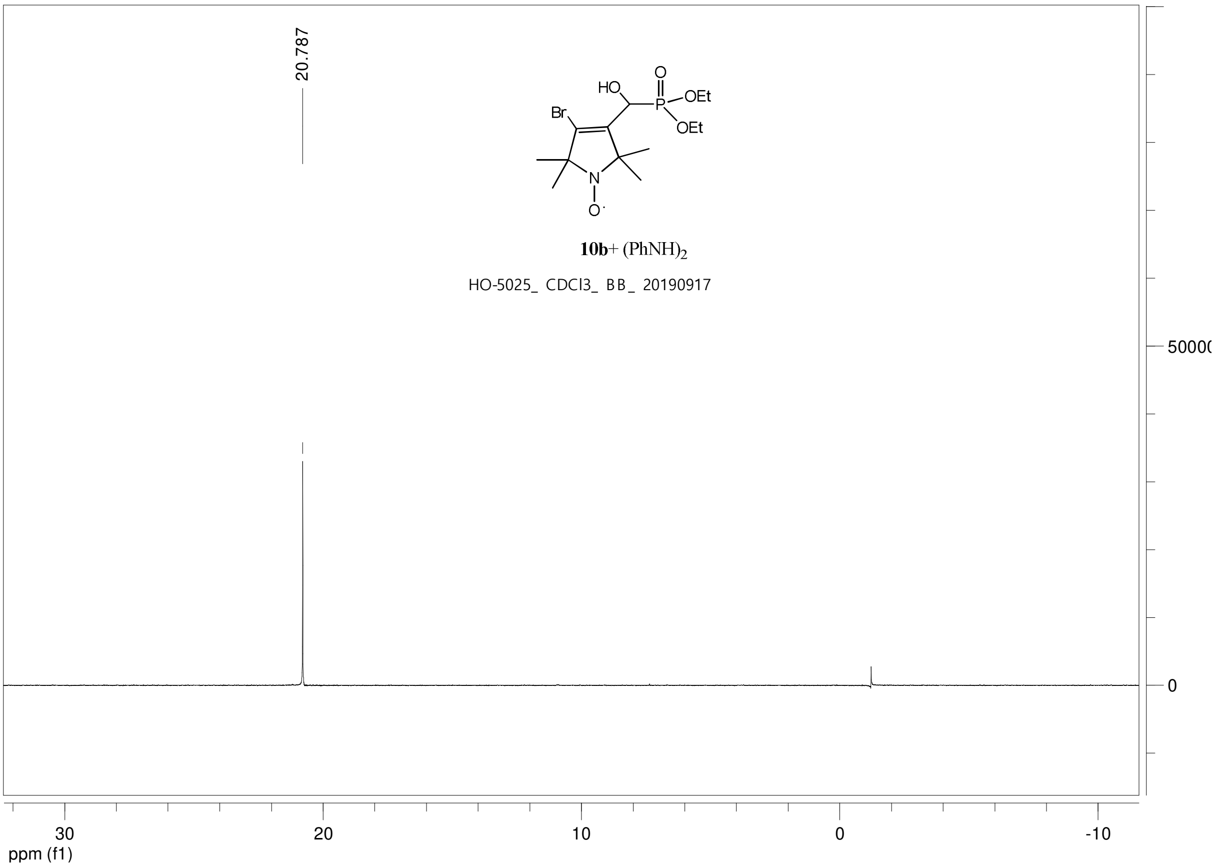
20.787
HO
O
P
OEt
OEt
Br
N
O·
10b+ (PhNH)2
HO-5025_ CDCl3_ BB_ 20190917
50000
0
30
20
10
0
-10
ppm (f1)

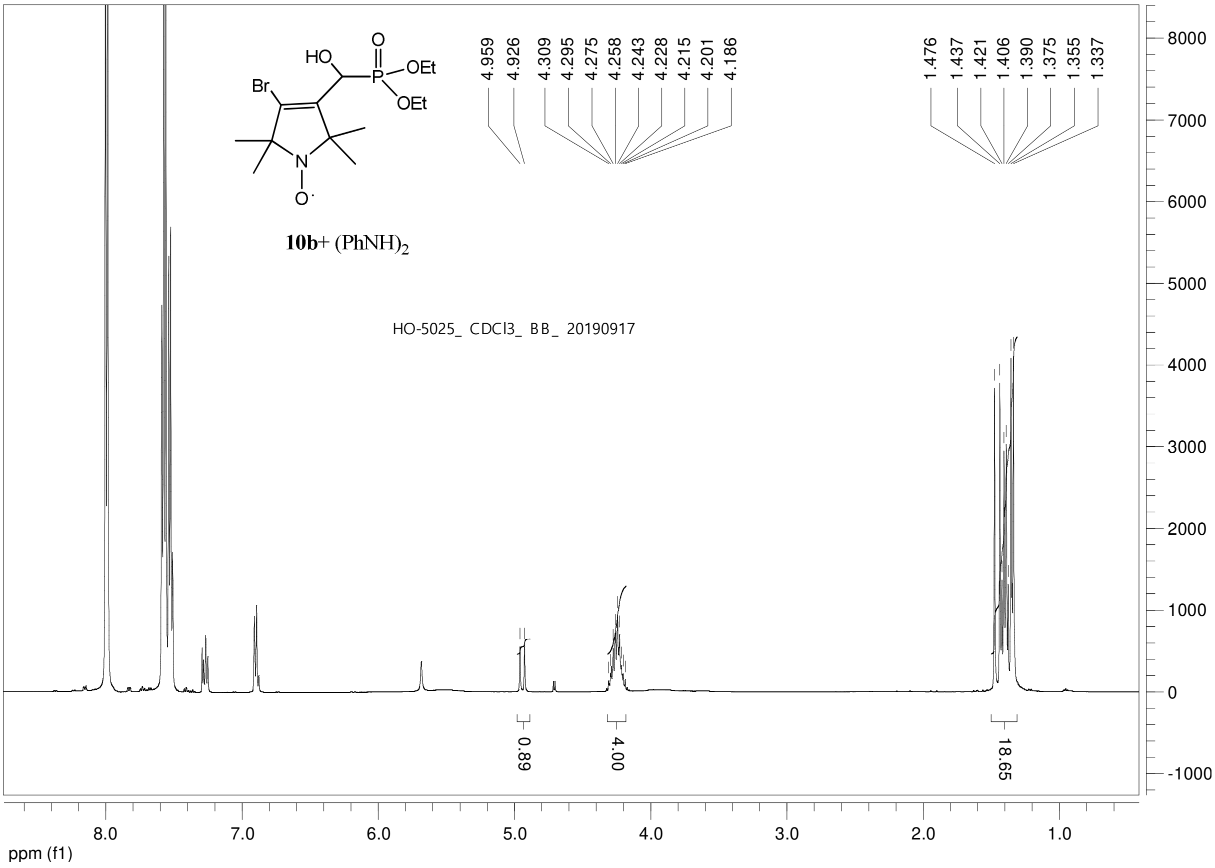
HO
O
P
OEt
OEt
Br
N
O·
10b+ (PhNH)2
4.959
4.926
4.309
4.295
4.275
4.258
4.243
4.228
4.215
4.201
4.186
1.476
1.437
1.421
1.406
1.390
1.375
1.355
1.337
HO-5025_ CDCl3_ BB_ 20190917
0.89
4.00
18.65
8000
7000
6000
5000
4000
3000
2000
1000
0
-1000
8.0
7.0
6.0
5.0
4.0
3.0
2.0
1.0
ppm (f1)

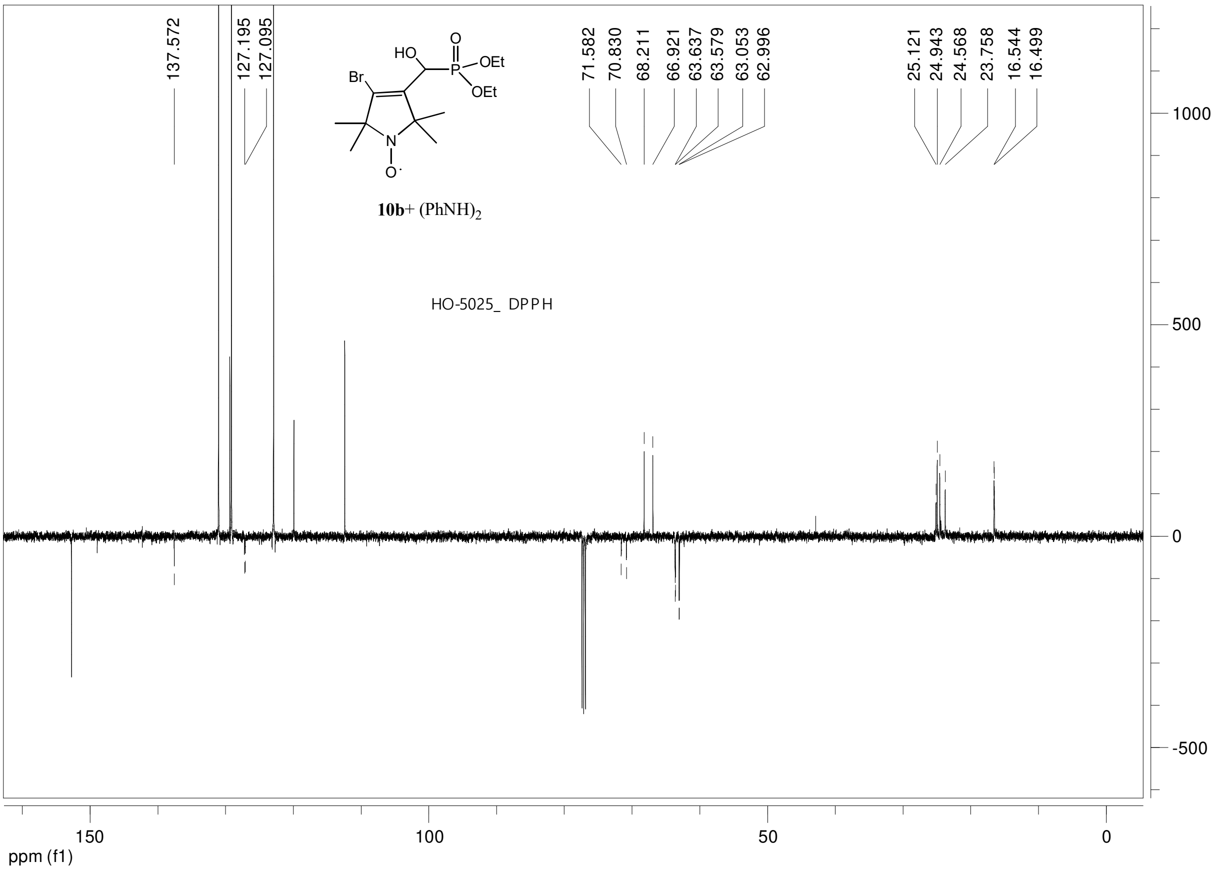
137.572
127.195
127.095
71.582
70.830
68.211
66.921
63.637
63.579
63.053
62.996
25.121
24.943
24.568
23.758
16.544
16.499
HO
O
P
OEt
OEt
Br
N
O
10b+ (PhNH)2
HO-5025_ DPPH
1000
500
0
-500
150
100
50
0
ppm (f1)

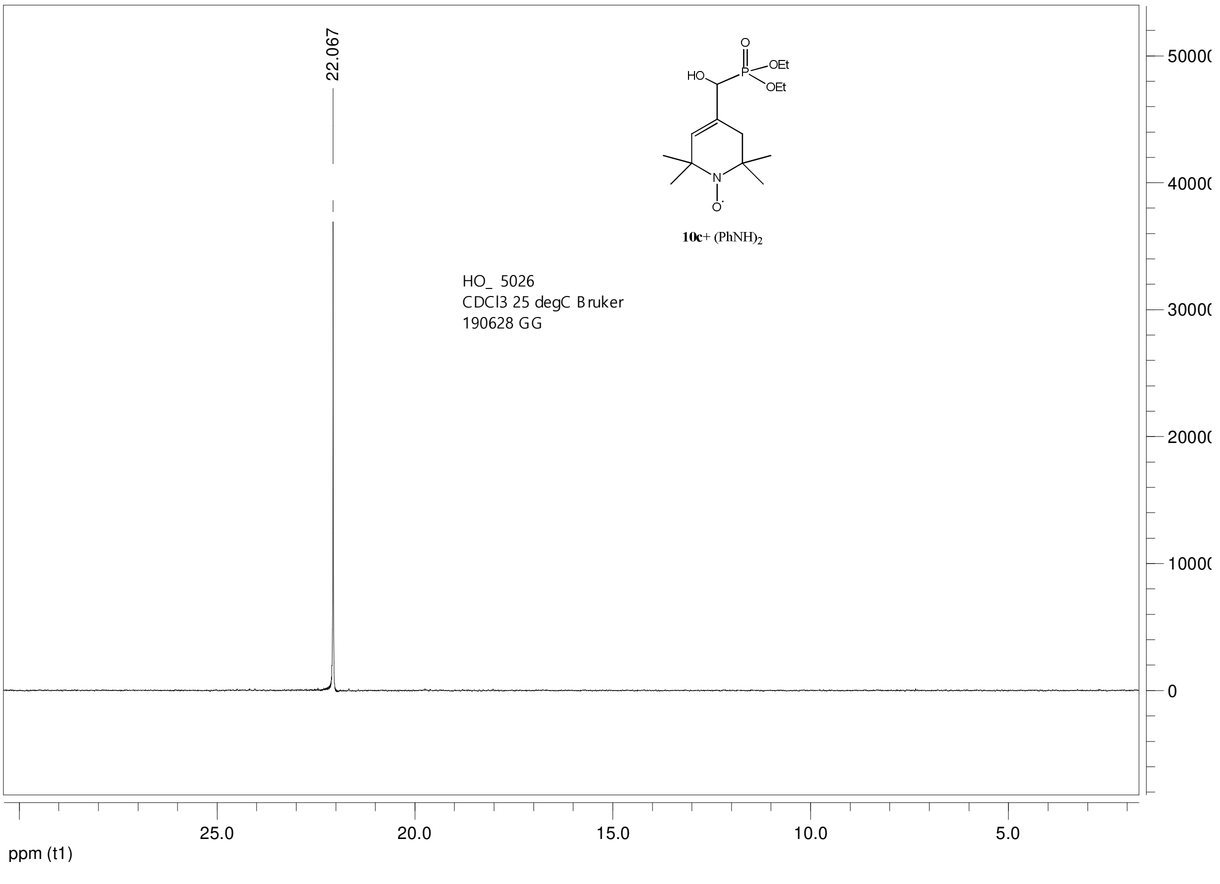
22.067
HO
O
P
OEt
OEt
N
O
10c+ (PhNH)2
HO_ 5026
CDCl3 25 degC Bruker
190628 GG
25.0
20.0
15.0
10.0
5.0
ppm (t1)

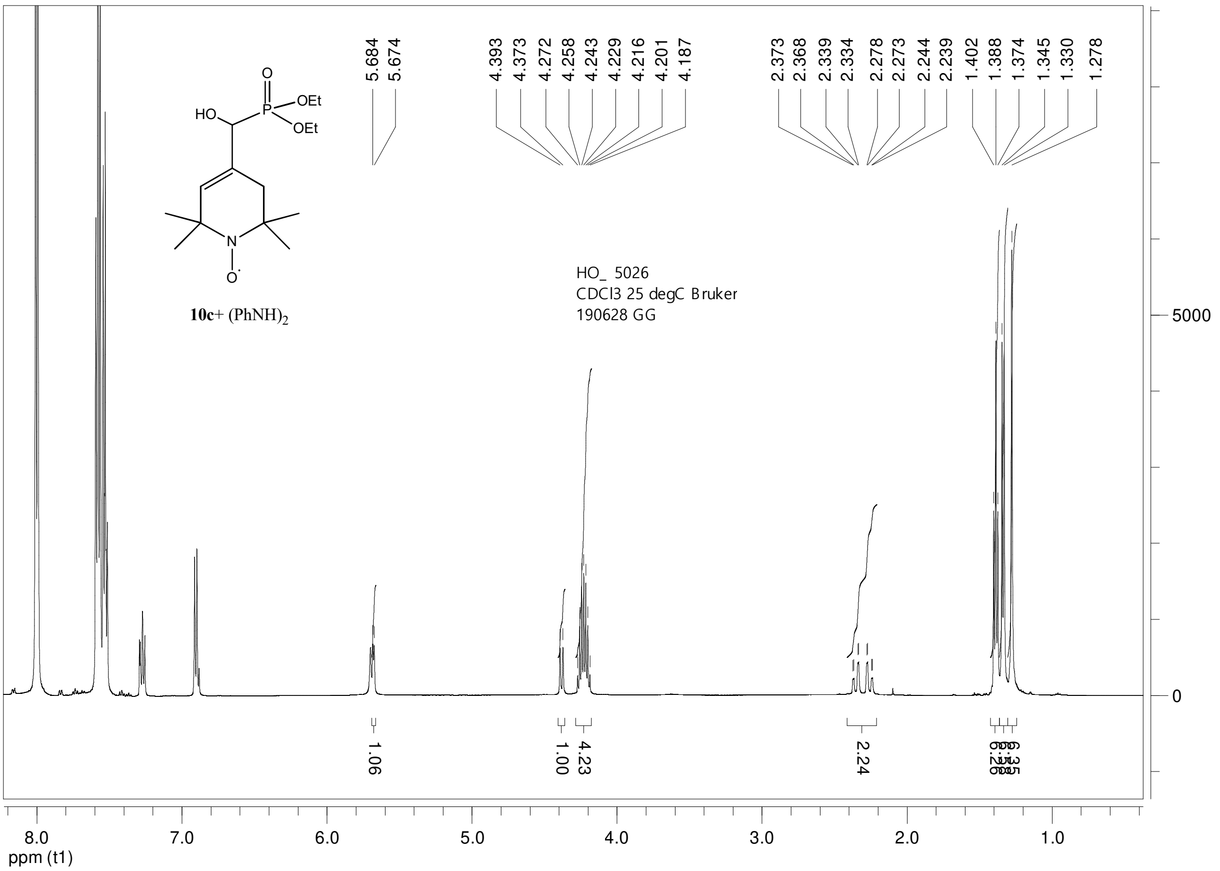
5.684
5.674
4.393
4.373
4.272
4.258
4.243
4.229
4.216
4.201
4.187
2.373
2.368
2.339
2.334
2.278
2.273
2.244
2.239
1.402
1.388
1.374
1.345
1.330
1.278
HO
O
P
OEt
OEt
N
O
10c+ (PhNH)2
HO_ 5026
CDCl3 25 degC Bruker
190628 GG
1.06
1.00
4.23
2.24
5000
0
8.0
7.0
6.0
5.0
4.0
3.0
2.0
1.0
ppm (t1)

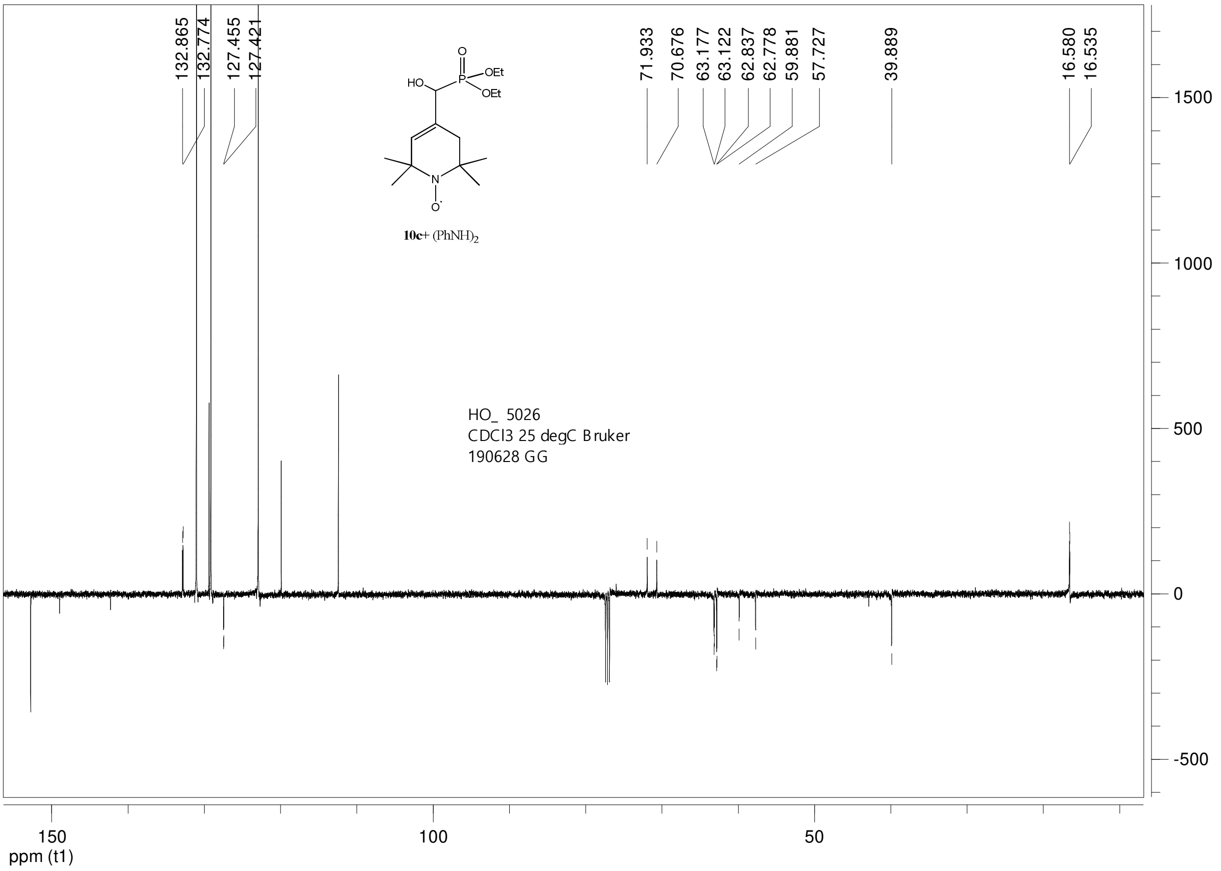
132.865
132.774
127.455
127.421
71.933
70.676
63.177
63.122
62.837
62.778
59.881
57.727
39.889
16.580
16.535
HO
O
P
OEt
OEt
N
O
10c+ (PhNH)2
HO_ 5026
CDCl3 25 degC Bruker
190628 GG
1500
1000
500
0
-500
150
100
50
ppm (t1)

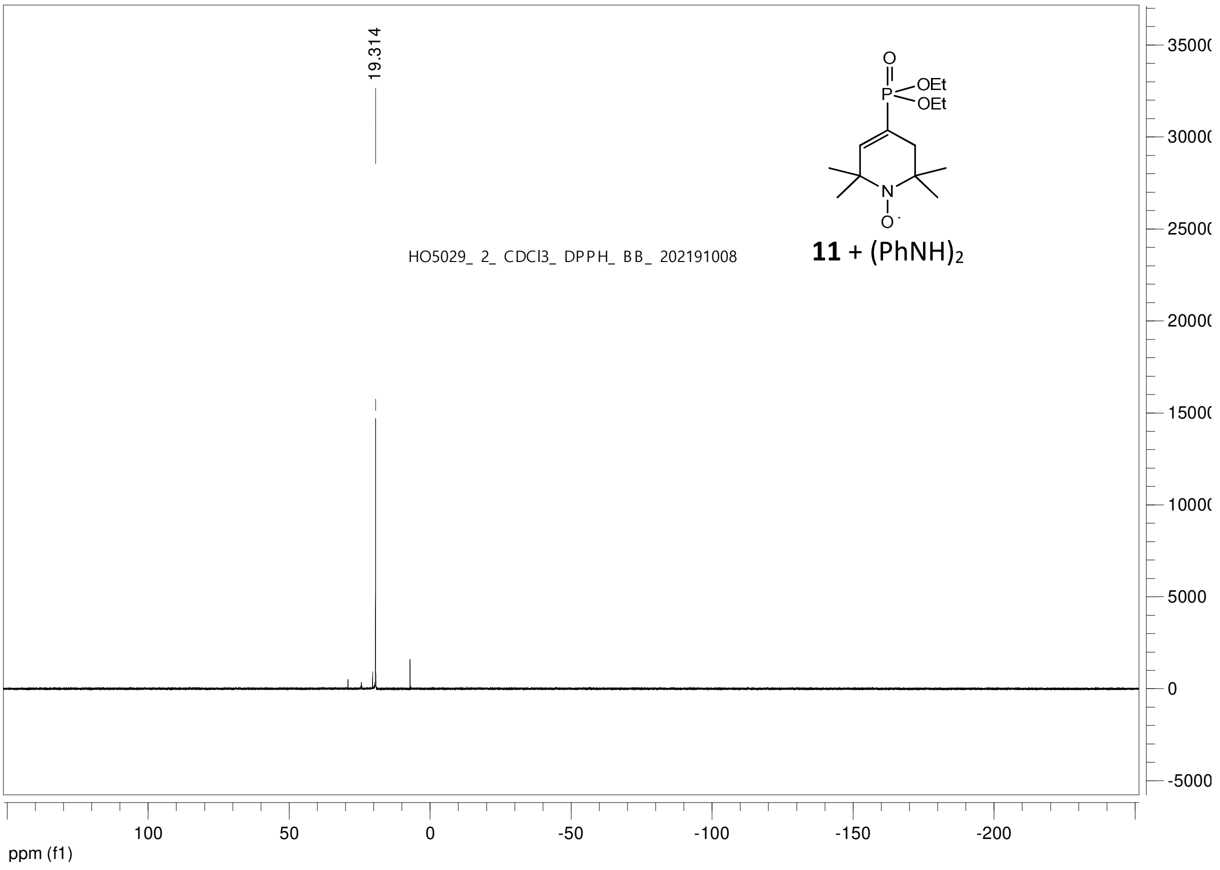
19.314
HO5029_ 2_ CDCl3_ DPPH_ BB_ 202191008
11 + (PhNH)2
ppm (f1)

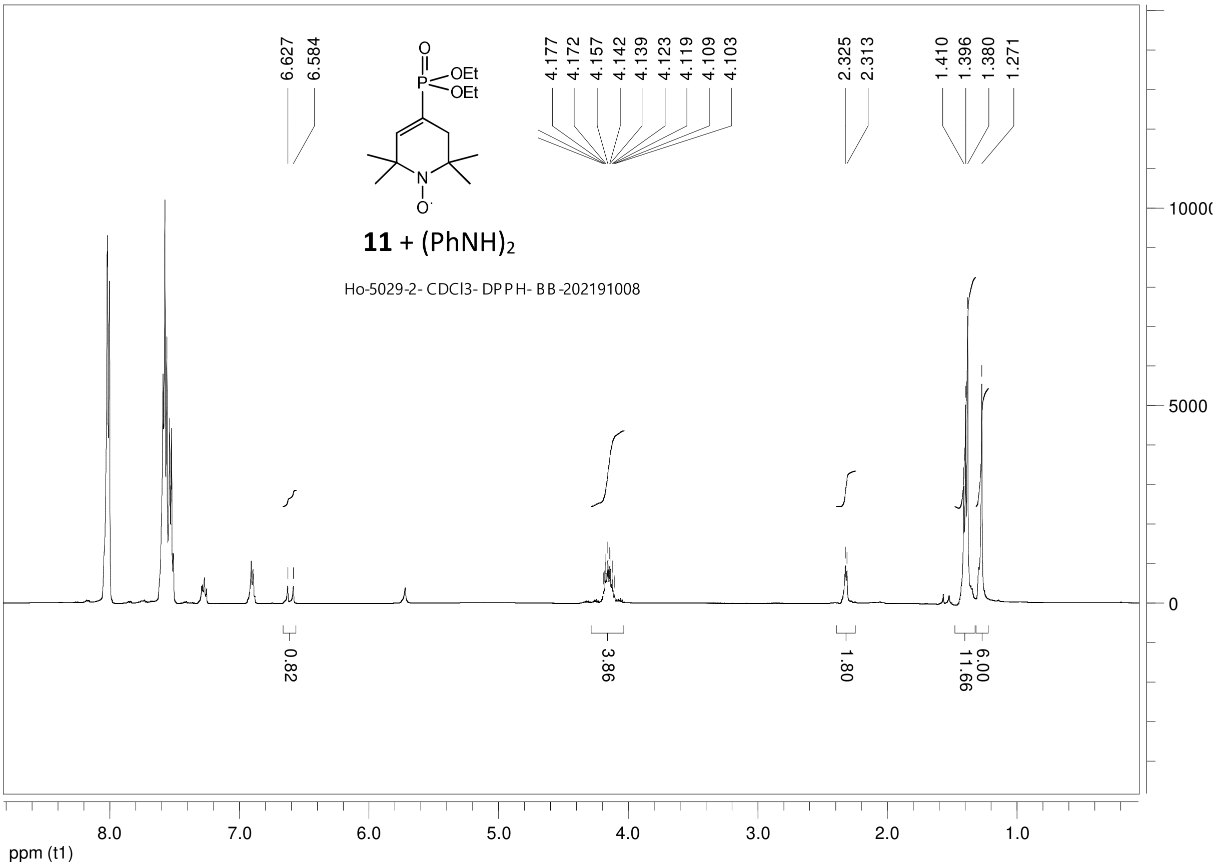
6.627
6.584
4.177
4.172
4.157
4.142
4.139
4.123
4.119
4.109
4.103
2.325
2.313
1.410
1.396
1.380
1.271
11 + (PhNH)2
Ho-5029-2- CDCl3- DPPH- BB-202191008
0.82
3.86
1.80
11.66
6.00
ppm (t1)

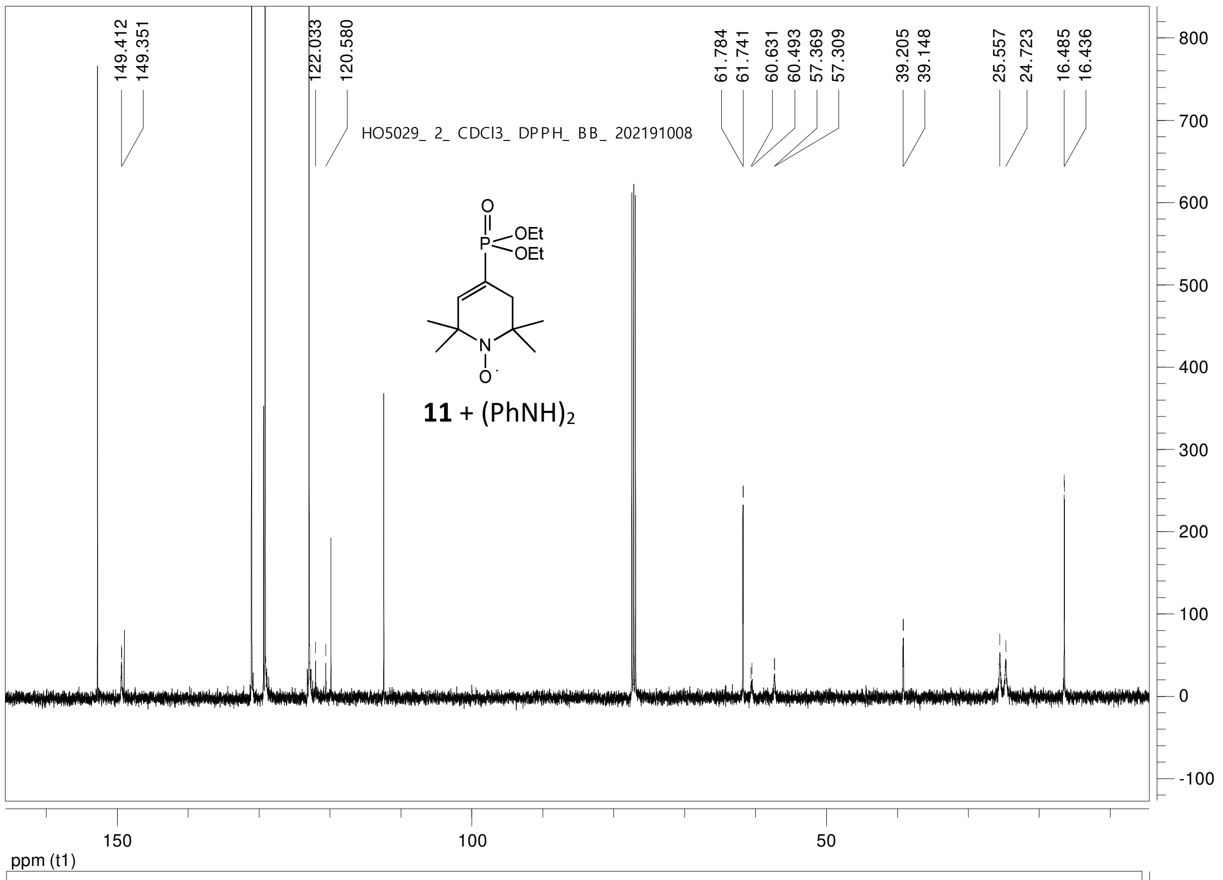
149.412
149.351
122.033
120.580
61.784
61.741
60.631
60.493
57.369
57.309
39.205
39.148
25.557
24.723
16.485
16.436
HO5029_ 2_ CDCl3_ DPPH_ BB_ 202191008
11 + (PhNH)2
ppm (t1)

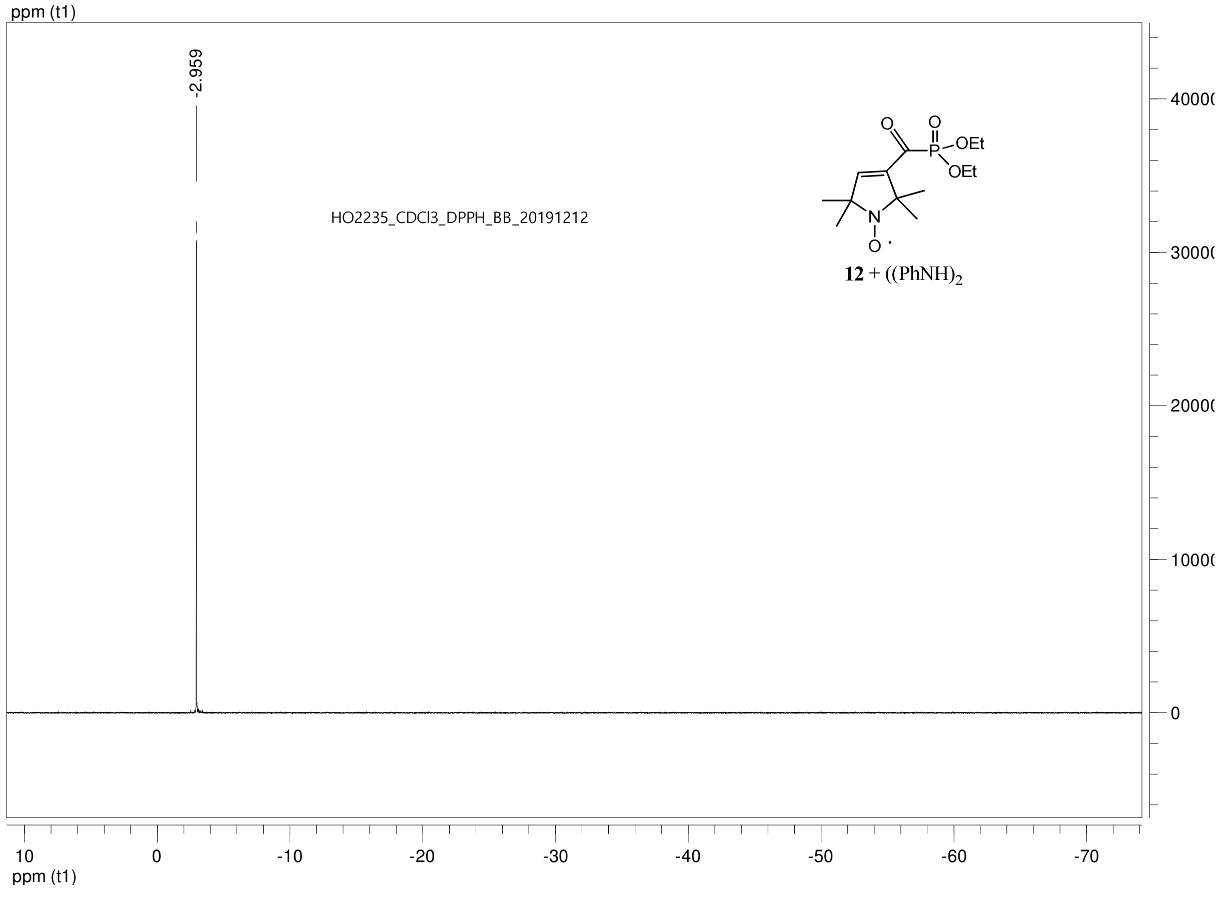
-2.959
HO2235_CDCl3_DPPH_BB_20191212
12 + ((PhNH)2
ppm (t1)

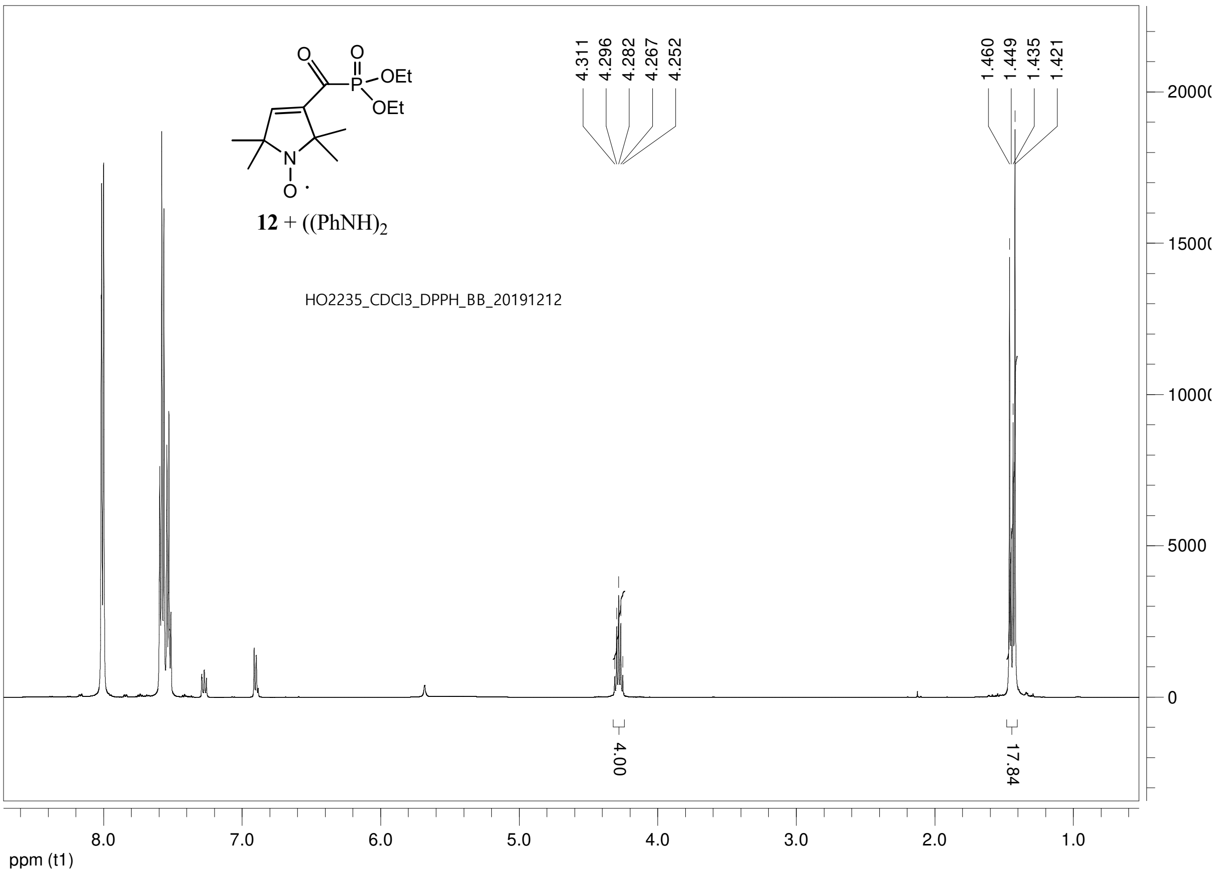

4.311
4.296
4.282
4.267
4.252
1.460
1.449
1.435
1.421
OEt
OEt
12 + ((PhNH)2
HO2235_CDCl3_DPPH_BB_20191212
4.00
17.84
8.0
7.0
6.0
5.0
4.0
3.0
2.0
1.0
ppm (t1)

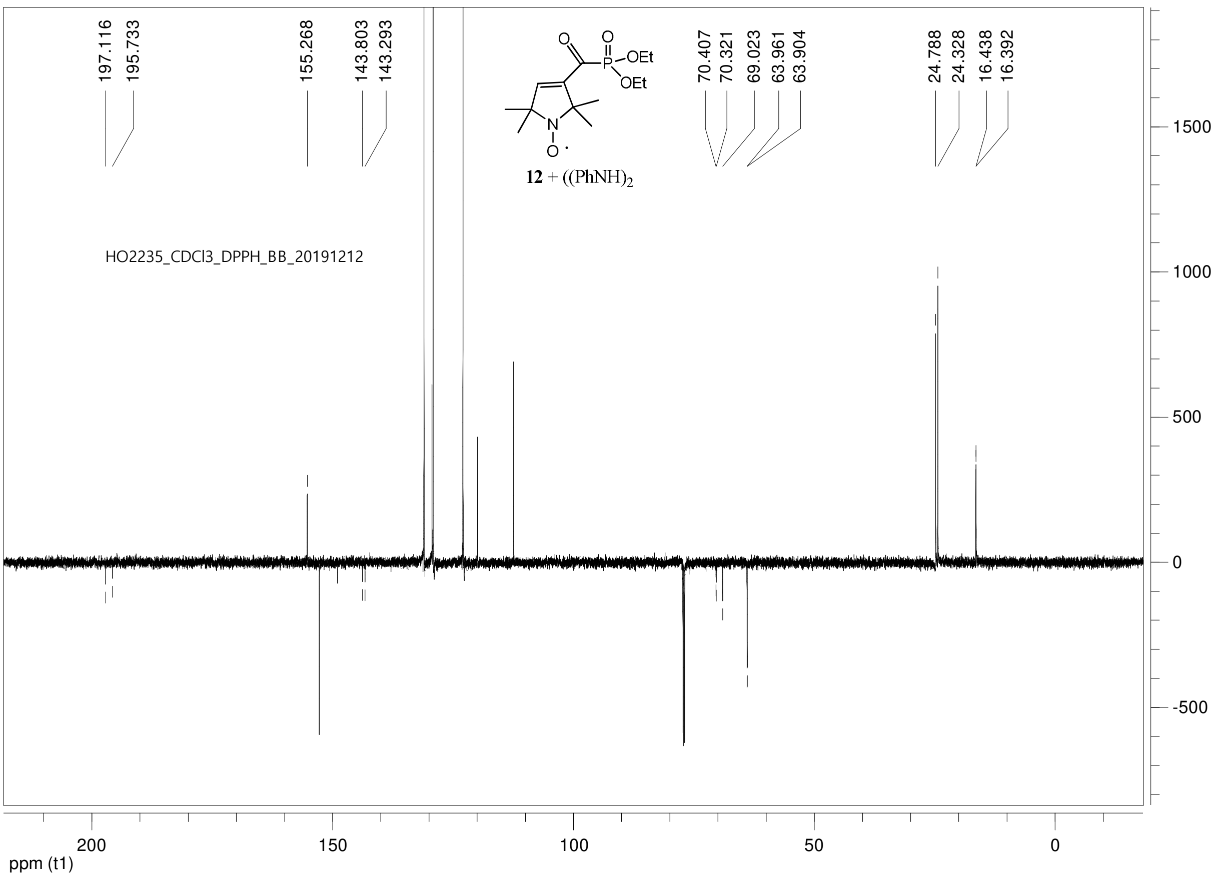

197.116
195.733
155.268
143.803
143.293
70.407
70.321
69.023
63.961
63.904
24.788
24.328
16.438
16.392
OEt
OEt
12 + ((PhNH)2
HO2235_CDCl3_DPPH_BB_20191212
1500
1000
500
0
-500
200
150
100
50
0
ppm (t1)

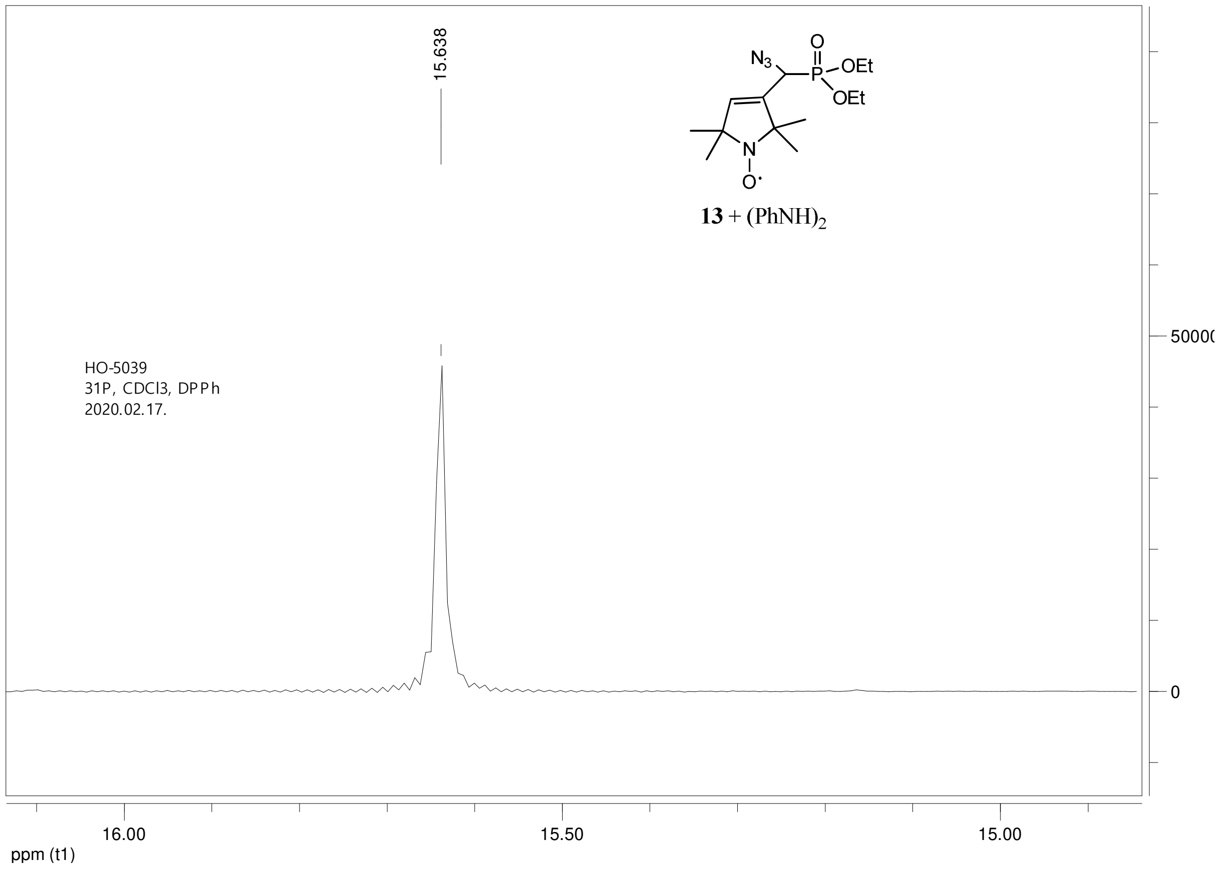
15.638
13 + $(PhNH)_2$
HO-5039
31P, CDCl3, DPPh
2020.02.17.
50000
0
16.00
15.50
15.00
ppm (t1)

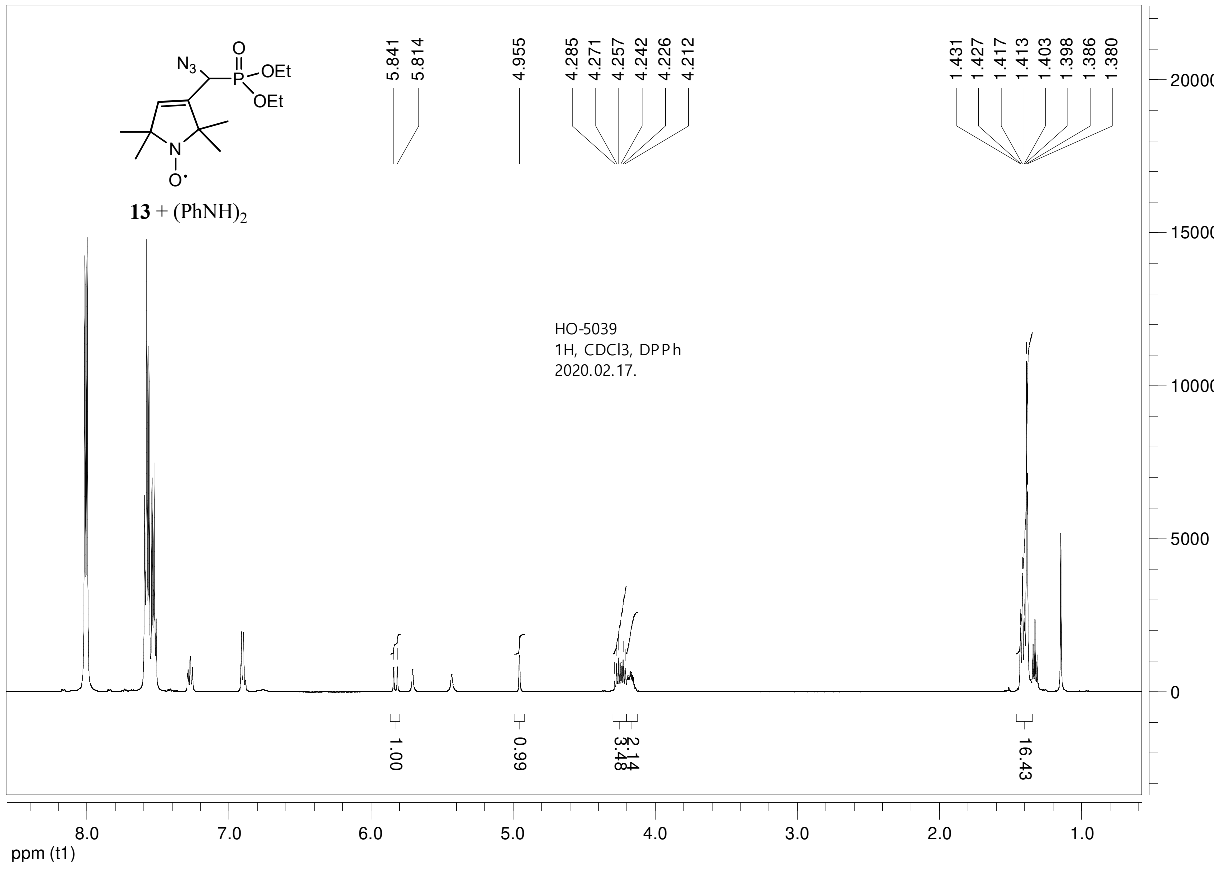
13 + $(PhNH)_2$
5.841
5.814
4.955
4.285
4.271
4.257
4.242
4.226
4.212
1.431
1.427
1.417
1.413
1.403
1.398
1.386
1.380
HO-5039
1H, CDCl3, DPPh
2020.02.17.
1.00
0.99
3.48
2.14
16.43
8.0
7.0
6.0
5.0
4.0
3.0
2.0
1.0
ppm (t1)

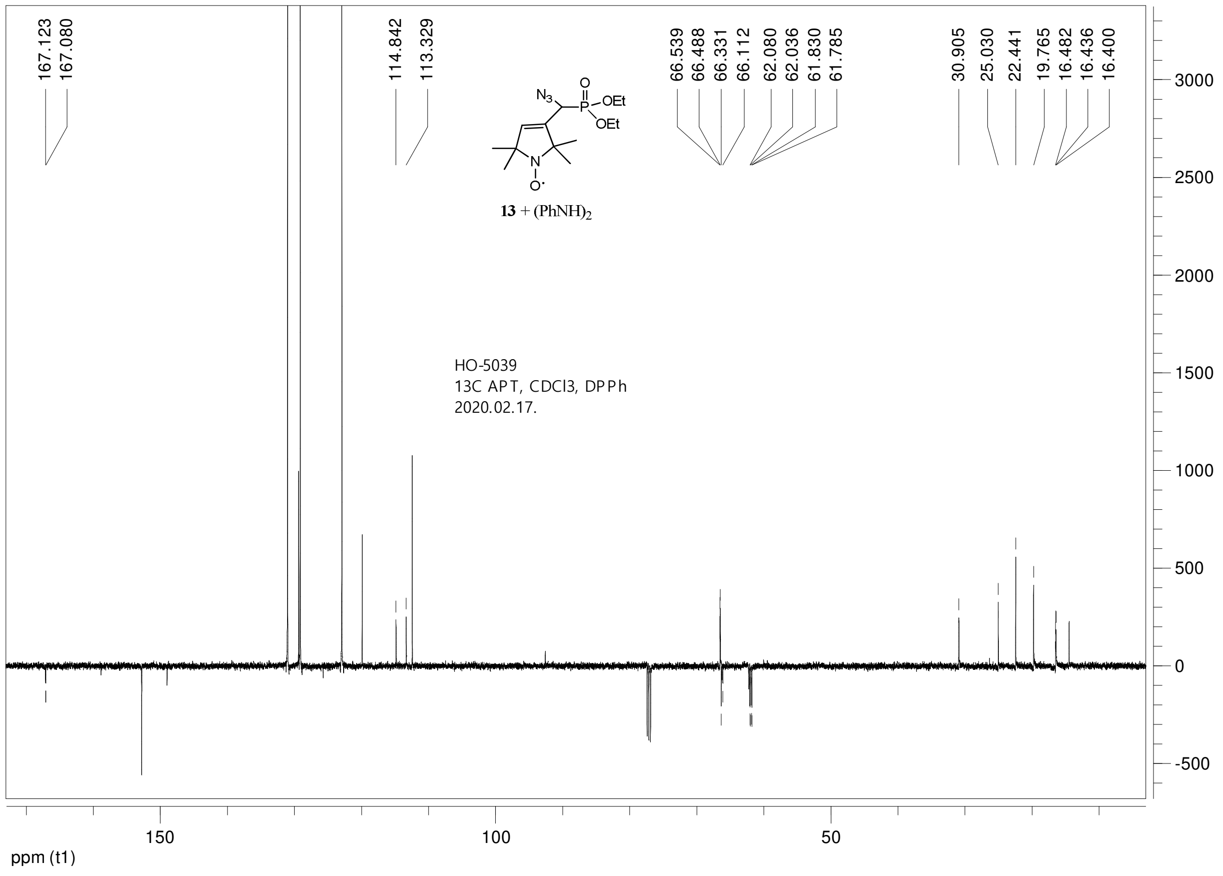

167.123
167.080
114.842
113.329
66.539
66.488
66.331
66.112
62.080
62.036
61.830
61.785
30.905
25.030
22.441
19.765
16.482
16.436
16.400
13 + (PhNH)2
HO-5039
13C APT, CDCl3, DPPh
2020.02.17.
ppm (t1)

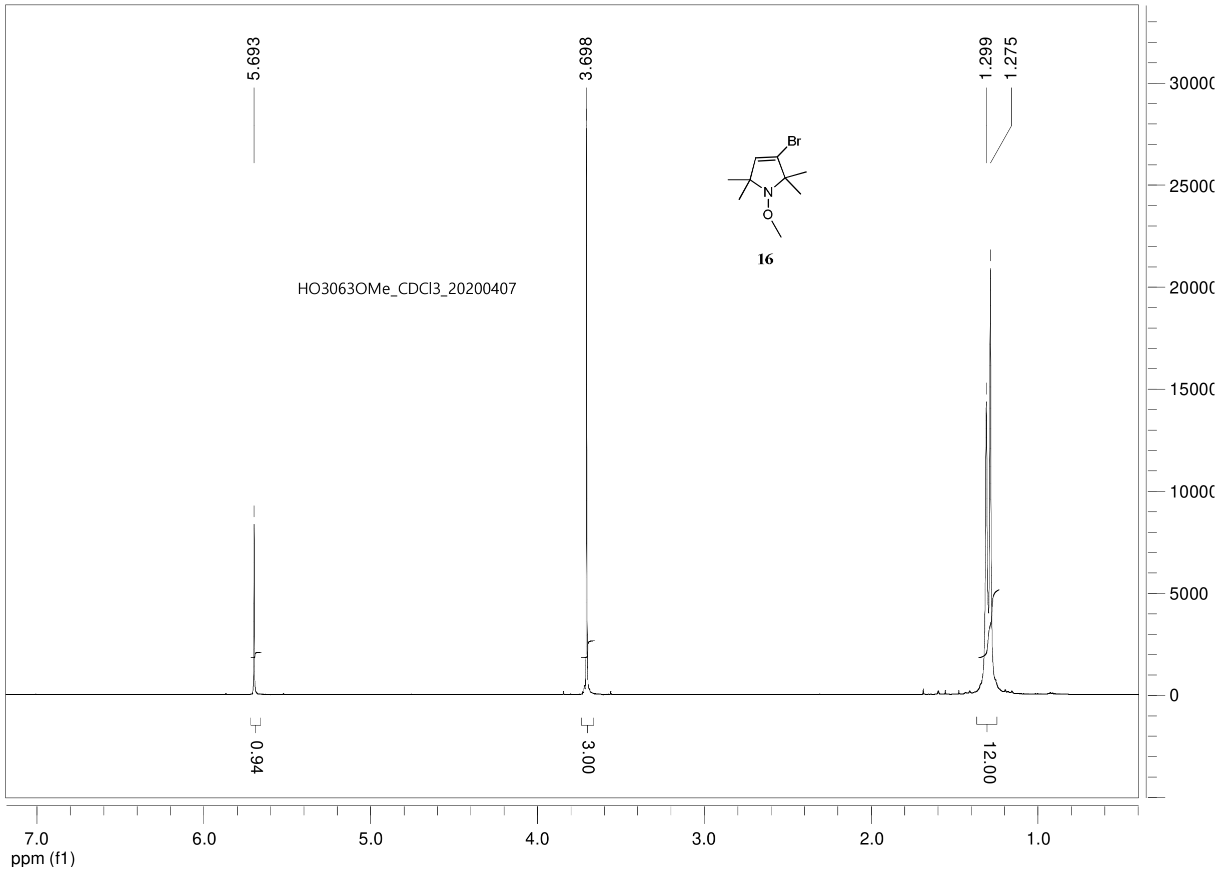

5.693
3.698
1.299
1.275
Br
16
HO3063OMe_CDCl3_20200407
0.94
3.00
12.00
ppm (f1)

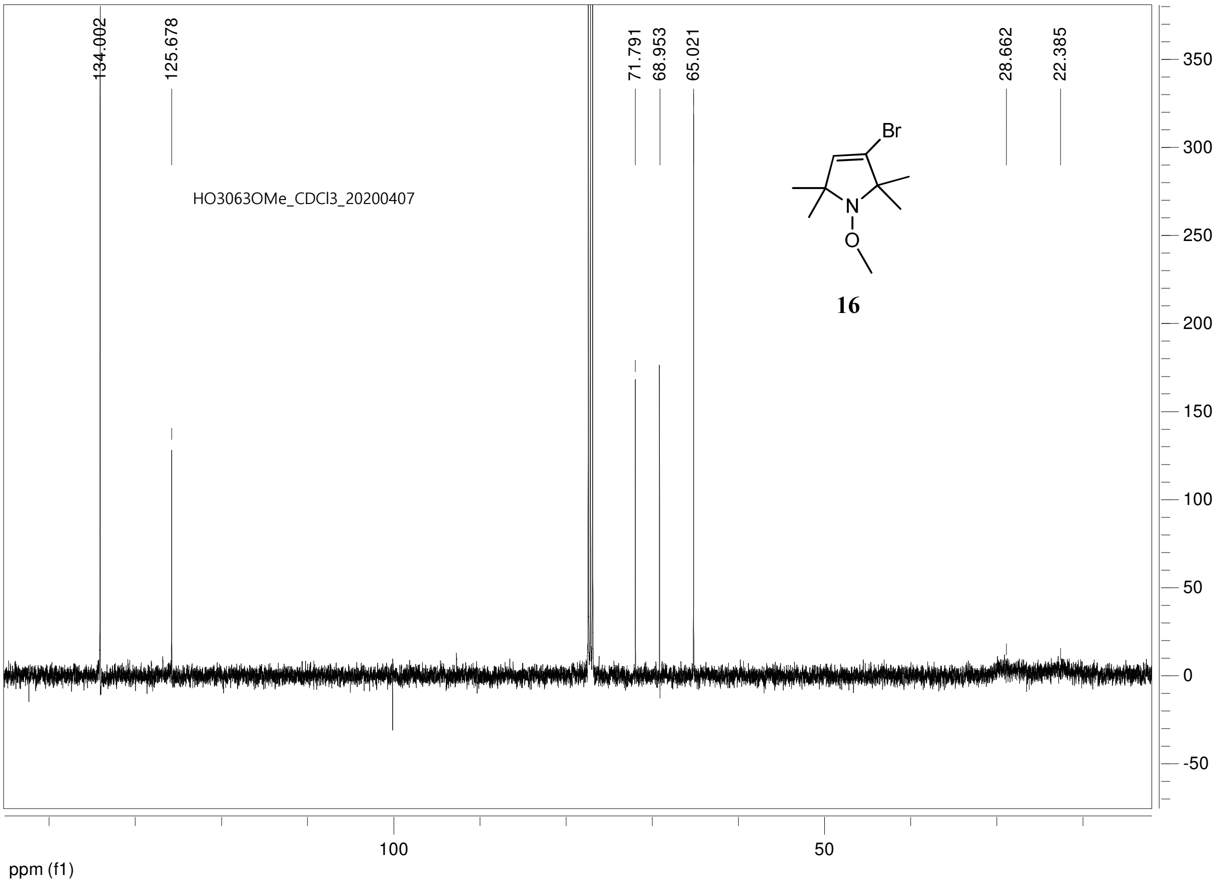

134.002
125.678
71.791
68.953
65.021
28.662
22.385
HO3063OMe_CDCl3_20200407
Br
N
O
16
350
300
250
200
150
100
50
0
-50
100
50
ppm (f1)

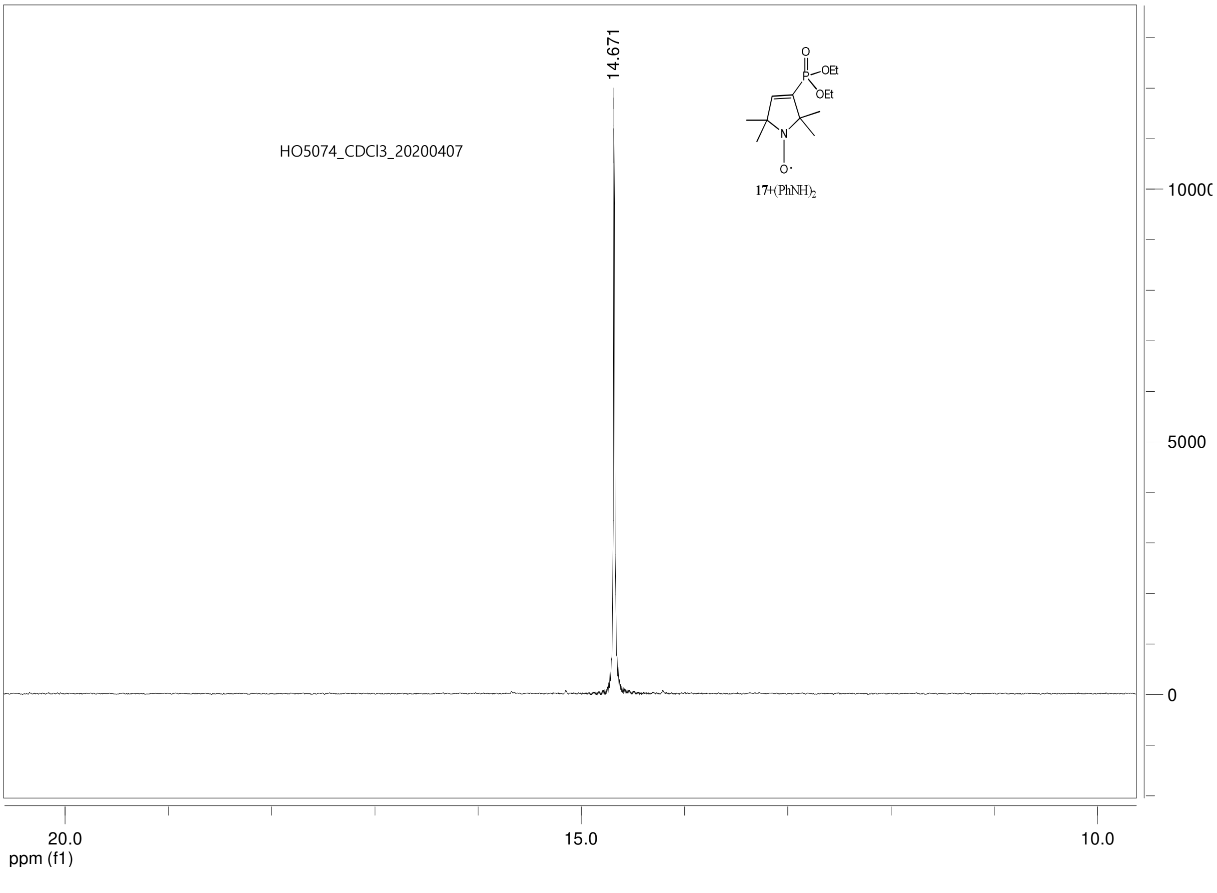

14.671
HO5074_CDCl3_20200407
O
P
OEt
OEt
N
O·
17+(PhNH)2
10000
5000
0
20.0
15.0
10.0
ppm (f1)

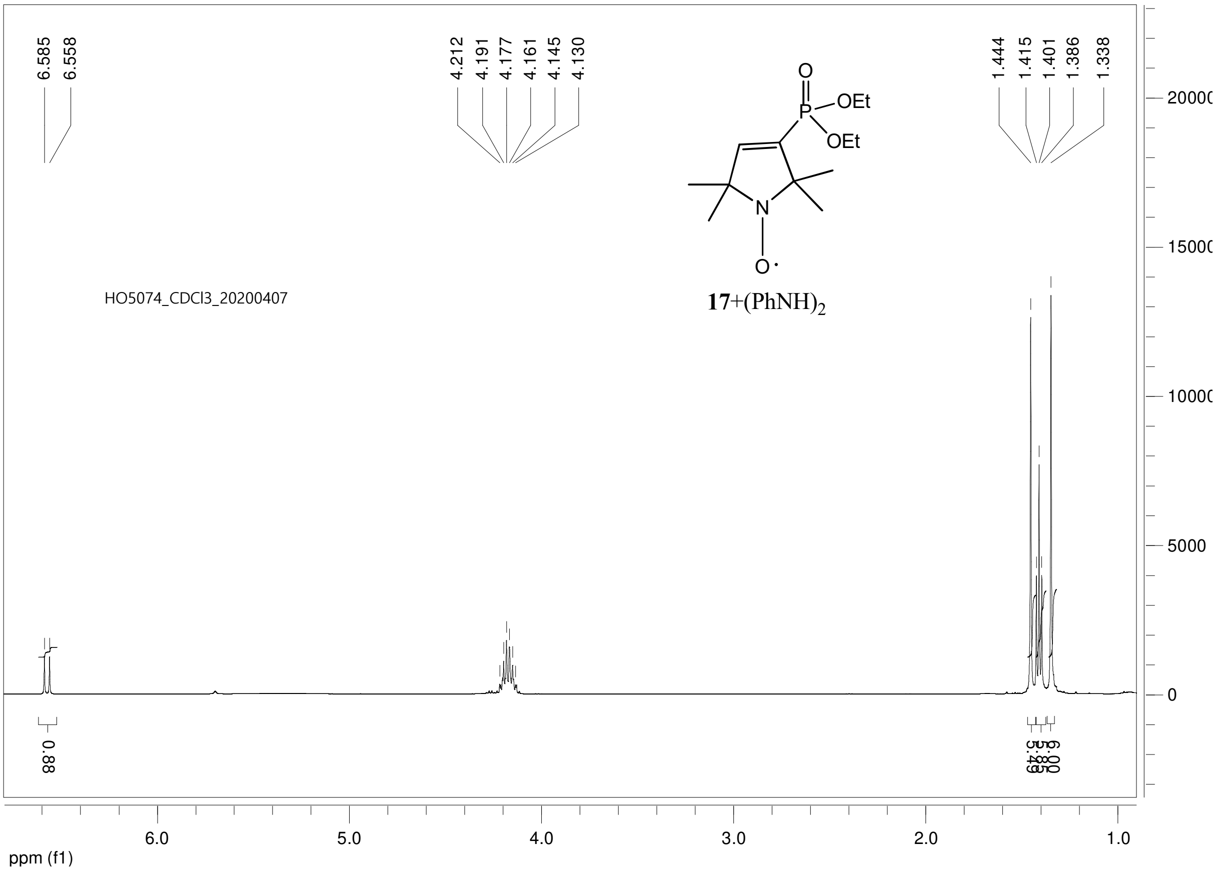
6.585
6.558
4.212
4.191
4.177
4.161
4.145
4.130
1.444
1.415
1.401
1.386
1.338
O
P
OEt
OEt
N
O·
HO5074_CDCl3_20200407
17+(PhNH)2
0.88
6.00
20000
15000
10000
5000
0
6.0
5.0
4.0
3.0
2.0
1.0
ppm (f1)

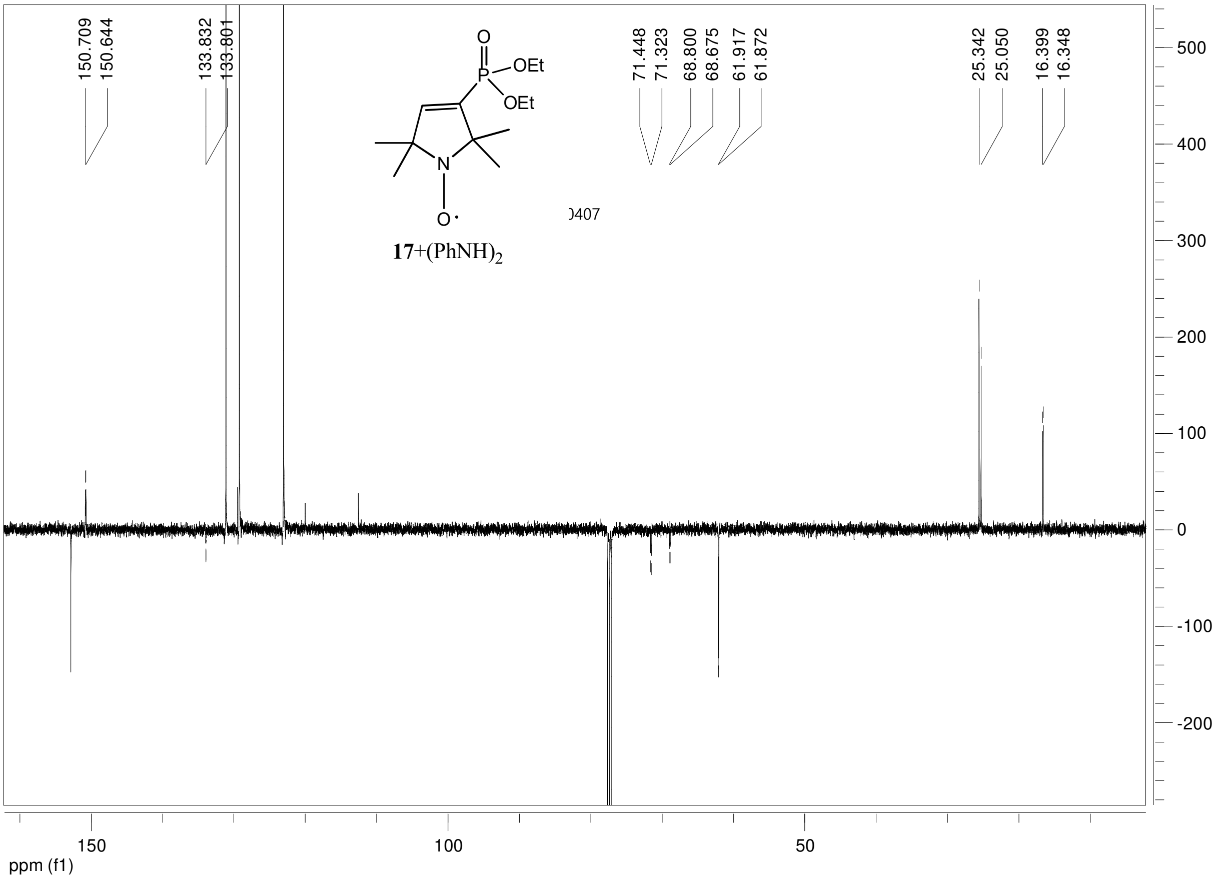
150.709
150.644
133.832
133.801
71.448
71.323
68.800
68.675
61.917
61.872
25.342
25.050
16.399
16.348
O
P
OEt
OEt
N
O·
17+(PhNH)2
500
400
300
200
100
0
-100
-200
150
100
50
ppm (f1)

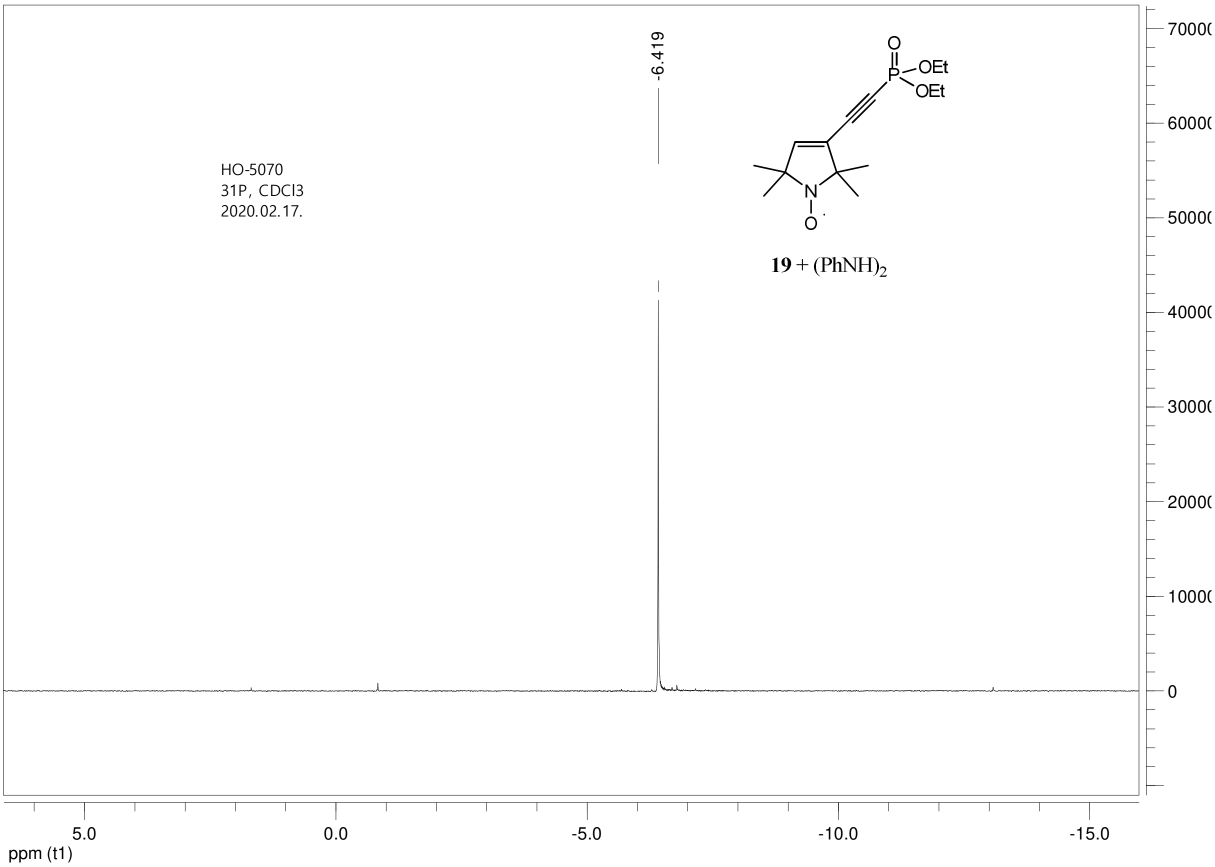
-6.419
HO-5070
31P, CDCl3
2020.02.17.
OEt
OEt
19 + (PhNH)2
5.0
0.0
-5.0
-10.0
-15.0
ppm (t1)

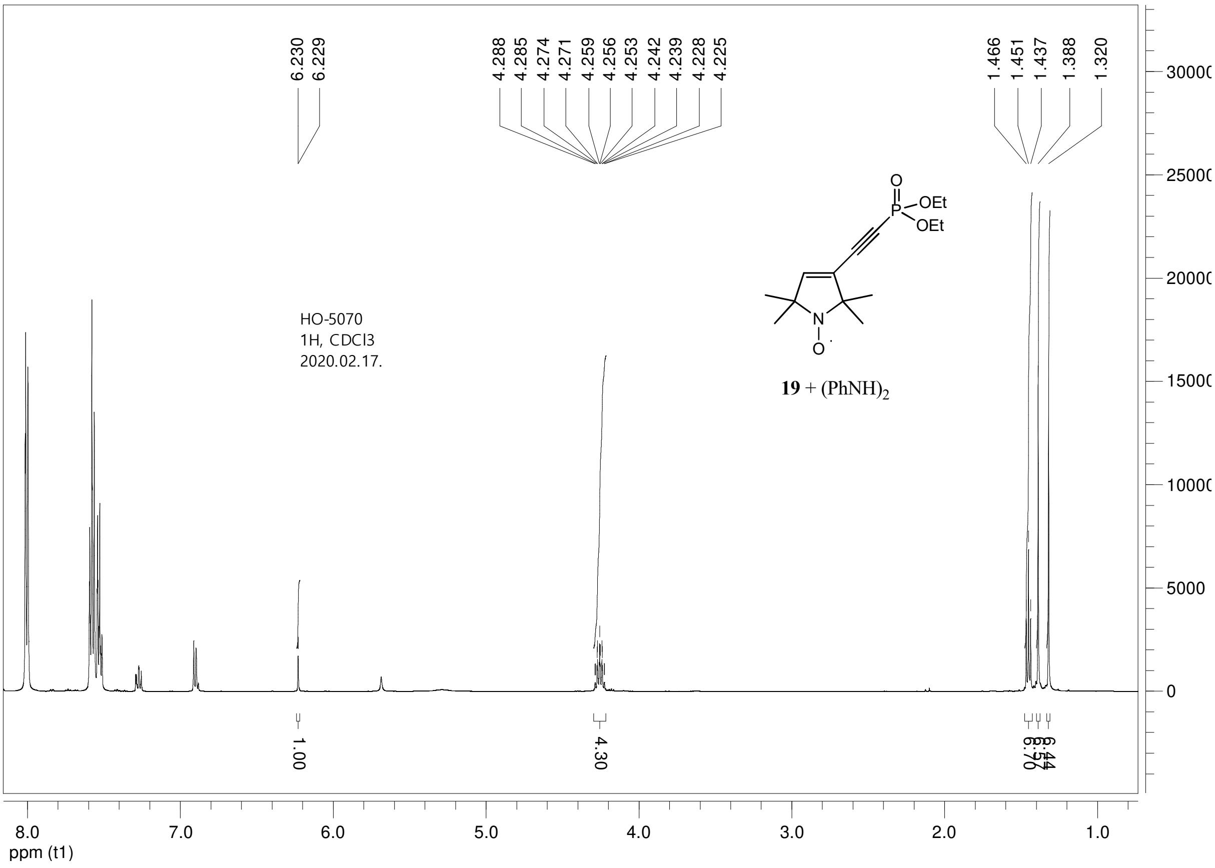
6.230
6.229
4.288
4.285
4.274
4.271
4.259
4.256
4.253
4.242
4.239
4.228
4.225
1.466
1.451
1.437
1.388
1.320
HO-5070
1H, CDCl3
2020.02.17.
OEt
OEt
19 + (PhNH)2
1.00
4.30
8.0
7.0
6.0
5.0
4.0
3.0
2.0
1.0
ppm (t1)

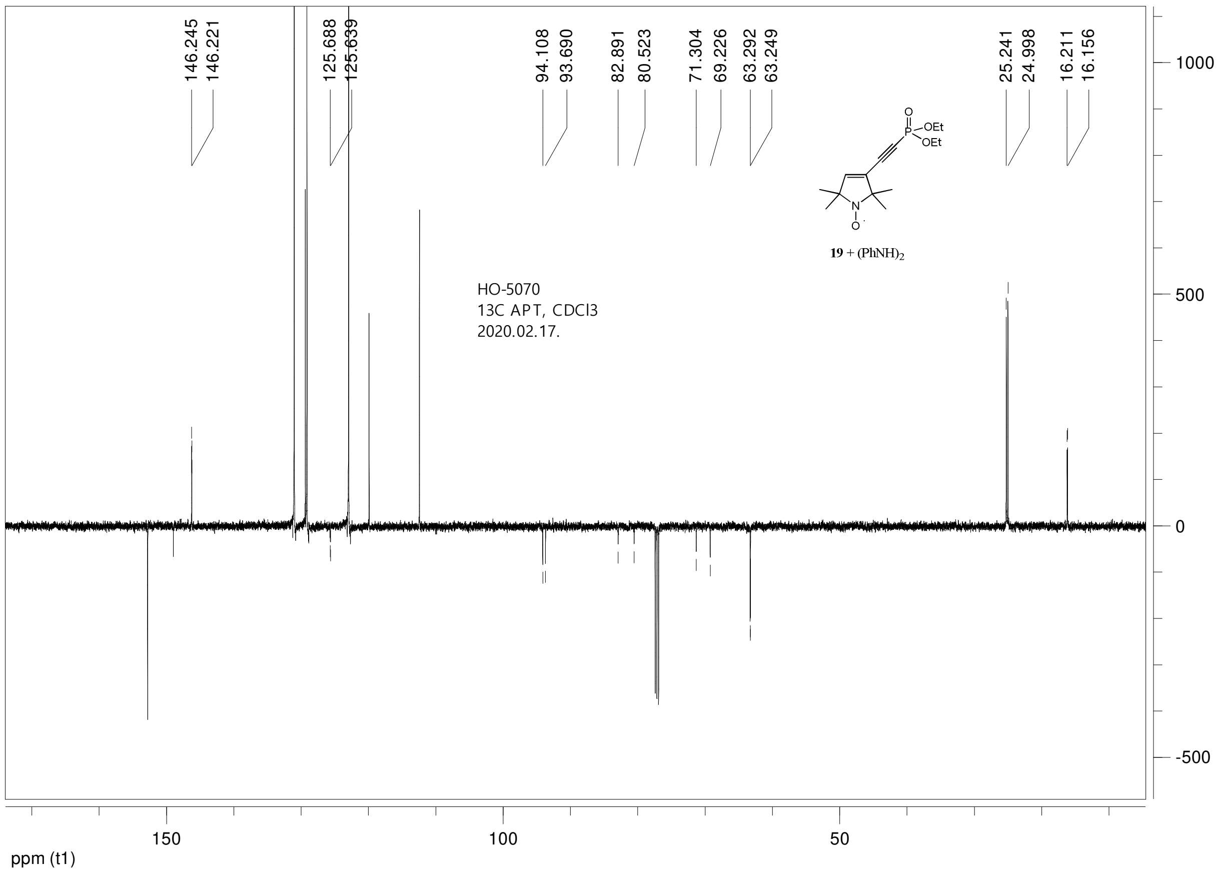
146.245
146.221
125.688
125.639
94.108
93.690
82.891
80.523
71.304
69.226
63.292
63.249
25.241
24.998
16.211
16.156
HO-5070
13C APT, CDCl3
2020.02.17.
19 + (PhNH)2
ppm (t1)

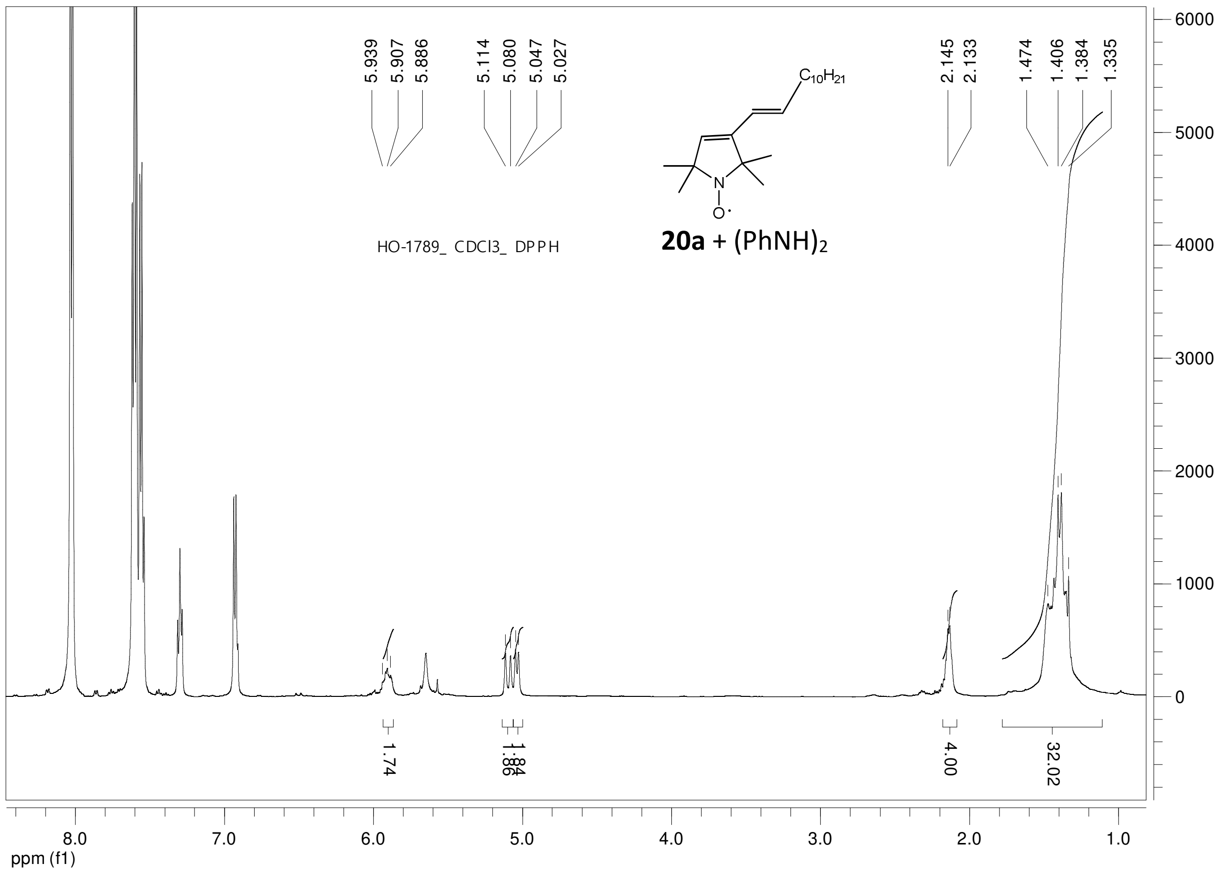
5.939
5.907
5.886
5.114
5.080
5.047
5.027
2.145
2.133
1.474
1.406
1.384
1.335
C10H21
HO-1789_ CDCl3_ DPPH
20a + (PhNH)2
1.74
1.86
1.84
4.00
32.02
ppm (f1)

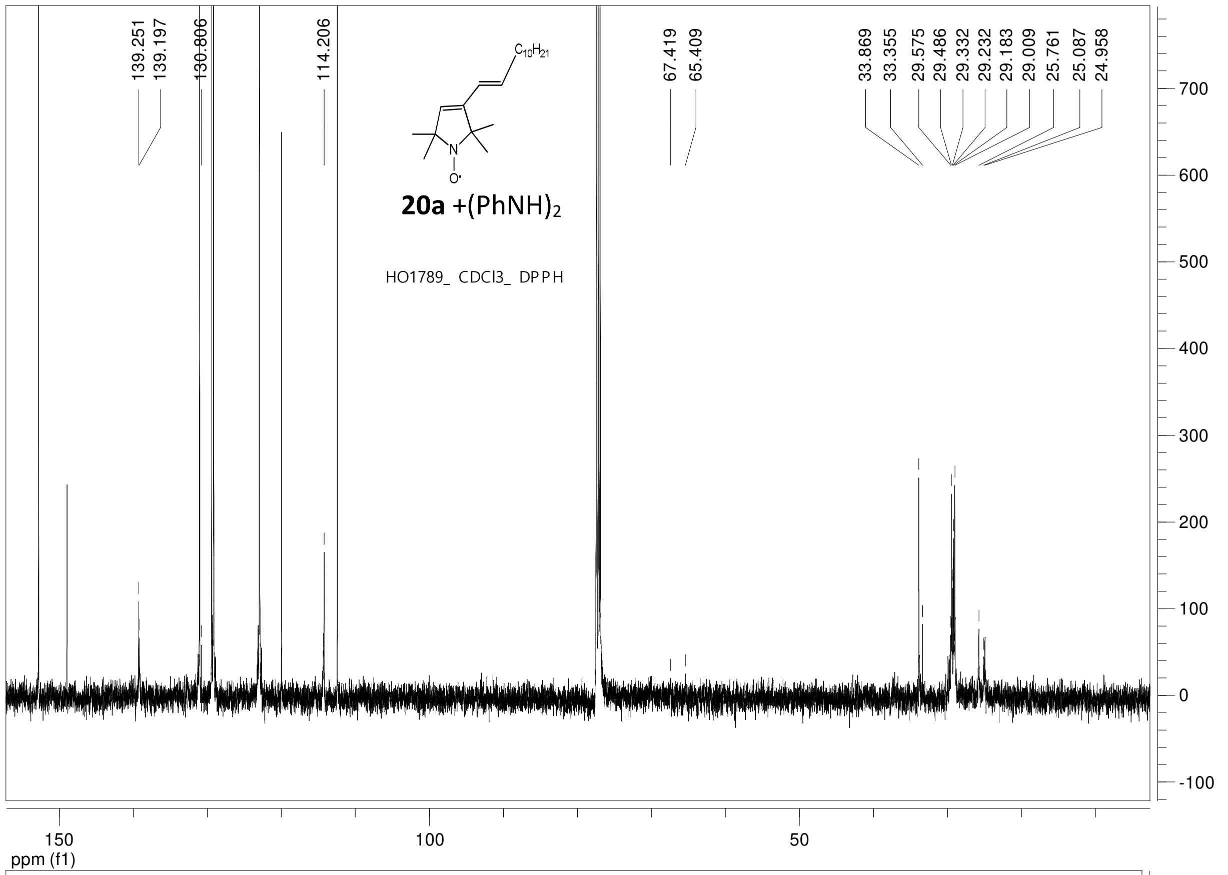
139.251
139.197
130.806
114.206
67.419
65.409
33.869
33.355
29.575
29.486
29.332
29.232
29.183
29.009
25.761
25.087
24.958
$C_{10}H_{21}$
N
O•
20a +$(PhNH)_2$
HO1789_ CDCl3_ DPPH
700
600
500
400
300
200
100
0
-100
150
100
50
ppm (f1)

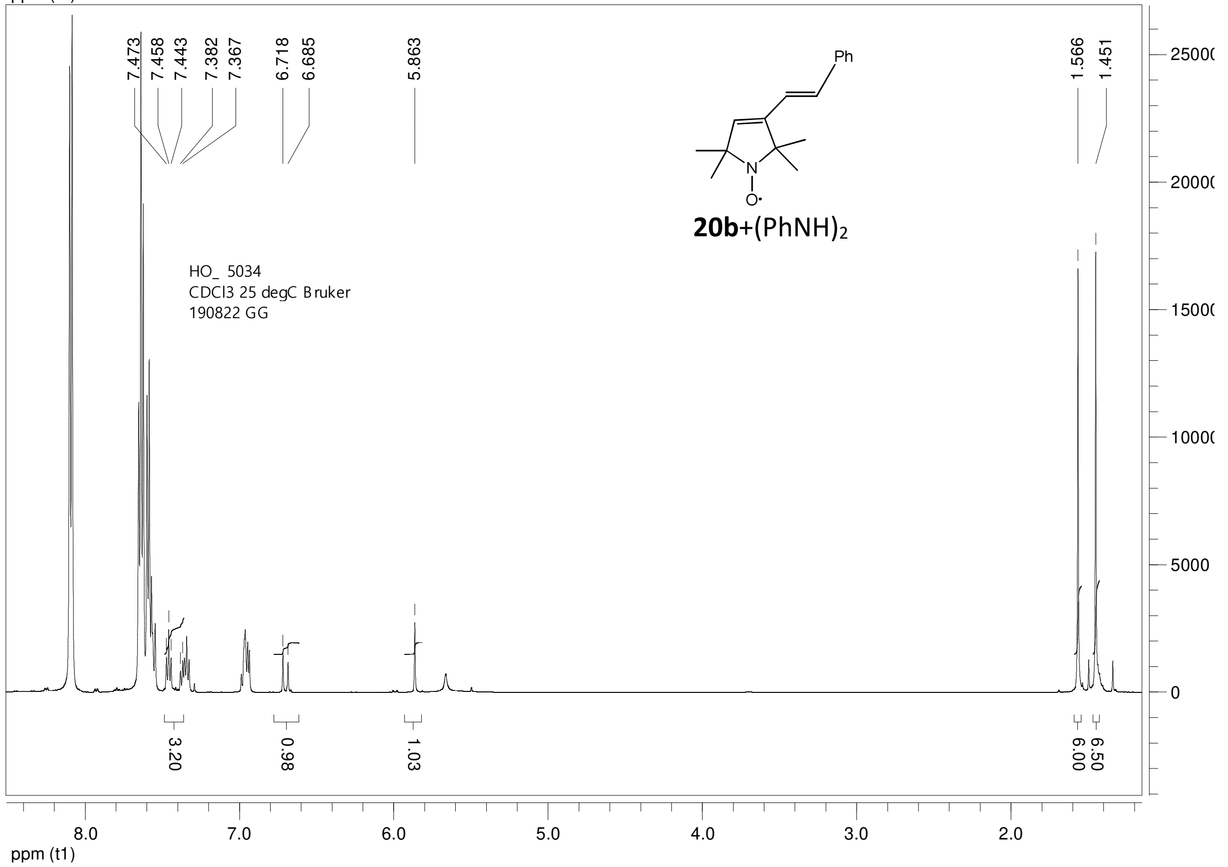
7.473
7.458
7.443
7.382
7.367
6.718
6.685
5.863
1.566
1.451
Ph
N
O•
20b+$(PhNH)_2$
HO_ 5034
CDCl3 25 degC Bruker
190822 GG
3.20
0.98
1.03
6.00
6.50
25000
20000
15000
10000
5000
0
8.0
7.0
6.0
5.0
4.0
3.0
2.0
ppm (t1)

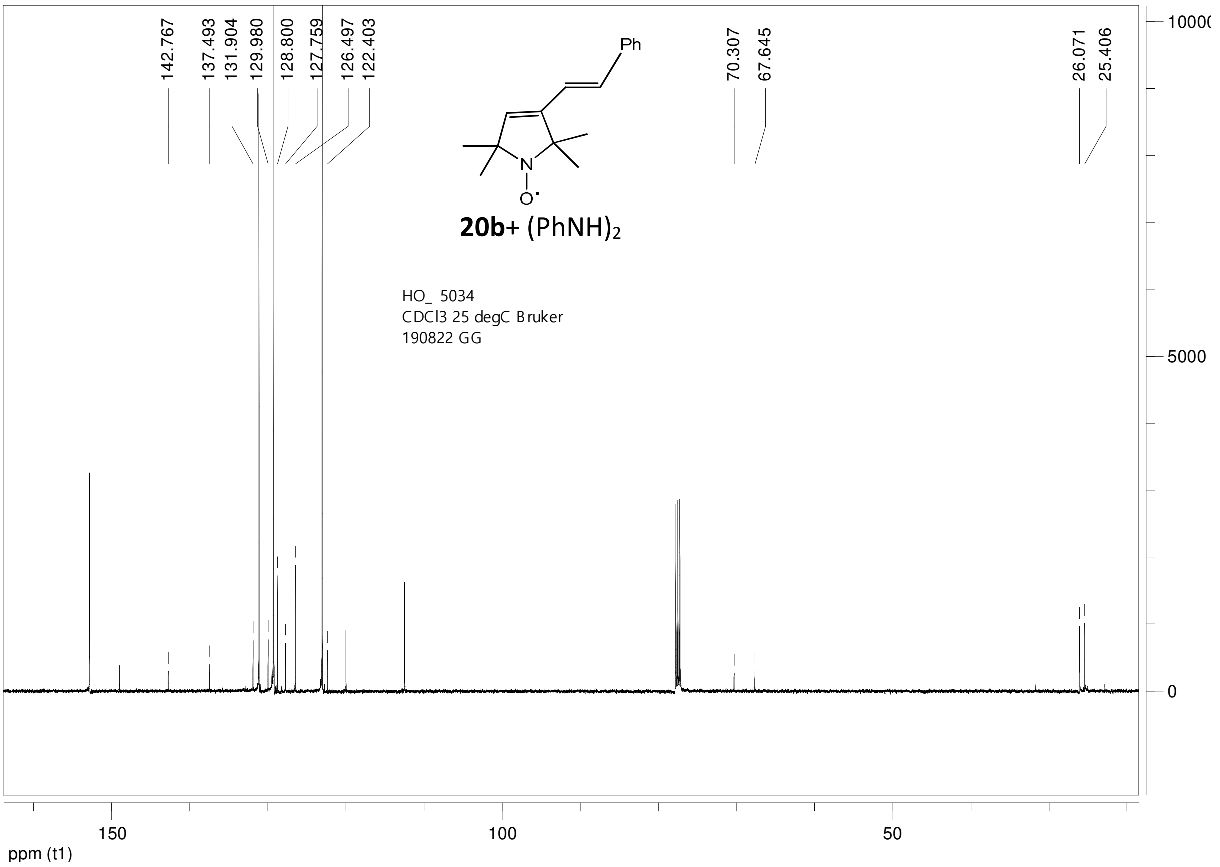
142.767
137.493
131.904
129.980
128.800
127.759
126.497
122.403
70.307
67.645
26.071
25.406
Ph
N
O•
20b+ (PhNH)$_2$
HO_ 5034
CDCl3 25 degC Bruker
190822 GG
10000
5000
0
150
100
50
ppm (t1)

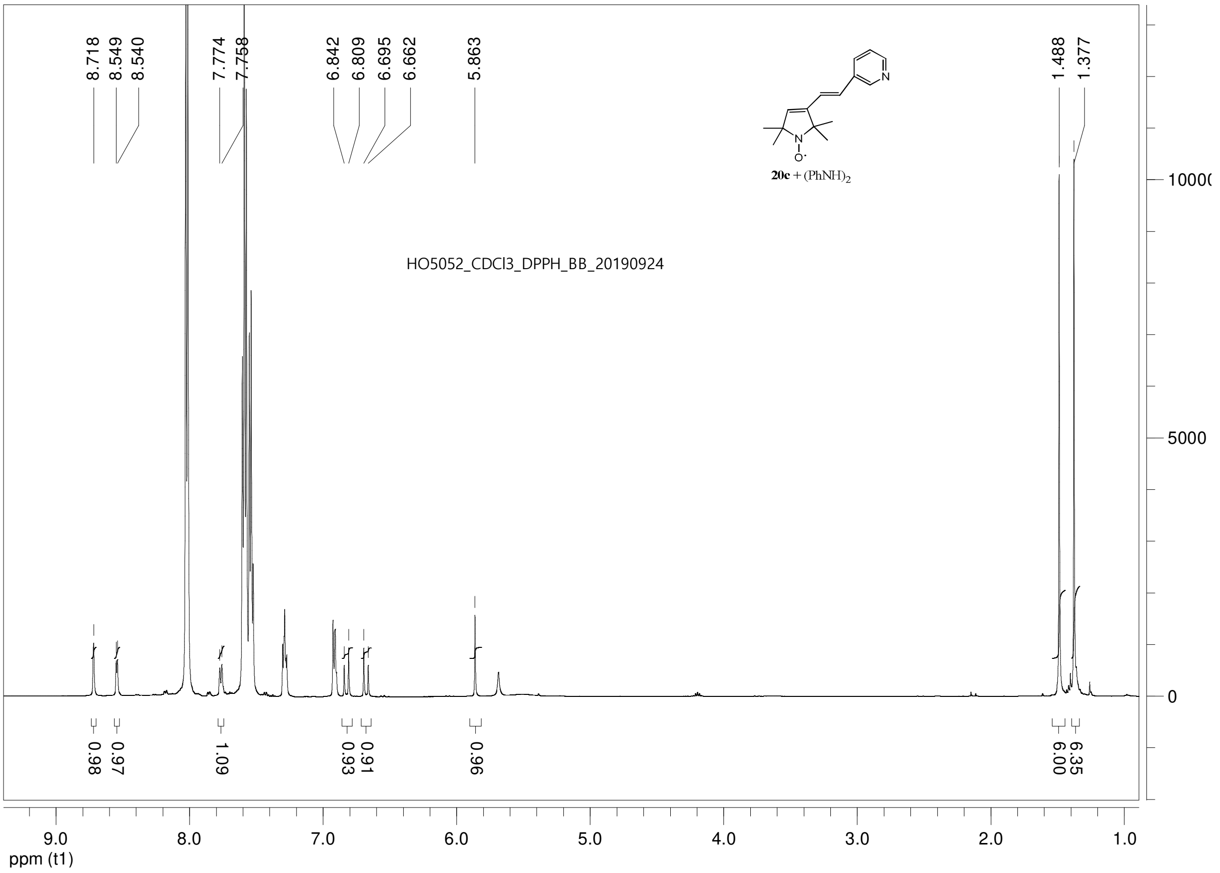
8.718
8.549
8.540
7.774
7.758
6.842
6.809
6.695
6.662
5.863
1.488
1.377
N
O•
20c + (PhNH)$_2$
HO5052_CDCl3_DPPH_BB_20190924
10000
5000
0
0.98
0.97
1.09
0.93
0.91
0.96
6.00
6.35
9.0
8.0
7.0
6.0
5.0
4.0
3.0
2.0
1.0
ppm (t1)

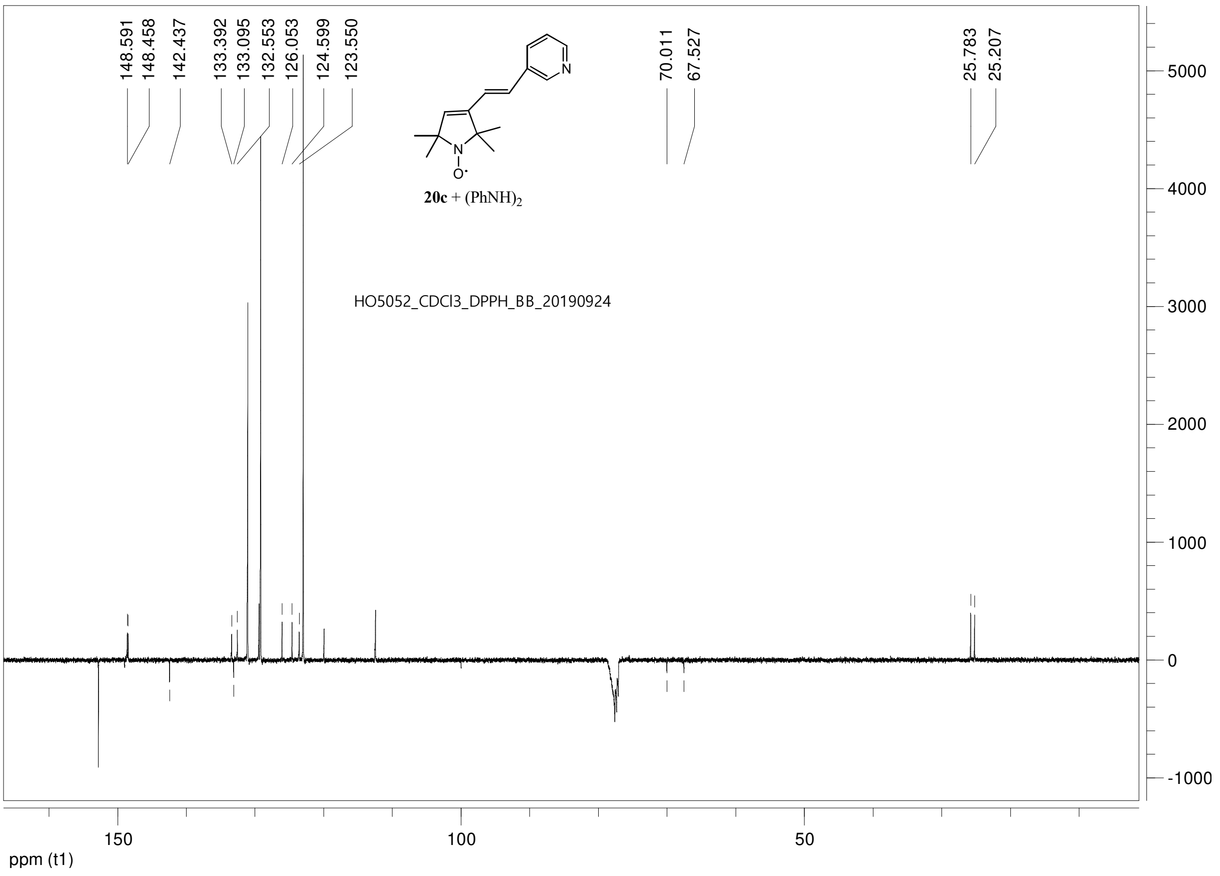
148.591
148.458
142.437
133.392
133.095
132.553
126.053
124.599
123.550
70.011
67.527
25.783
25.207
20c + (PhNH)2
HO5052_CDCl3_DPPH_BB_20190924
ppm (t1)

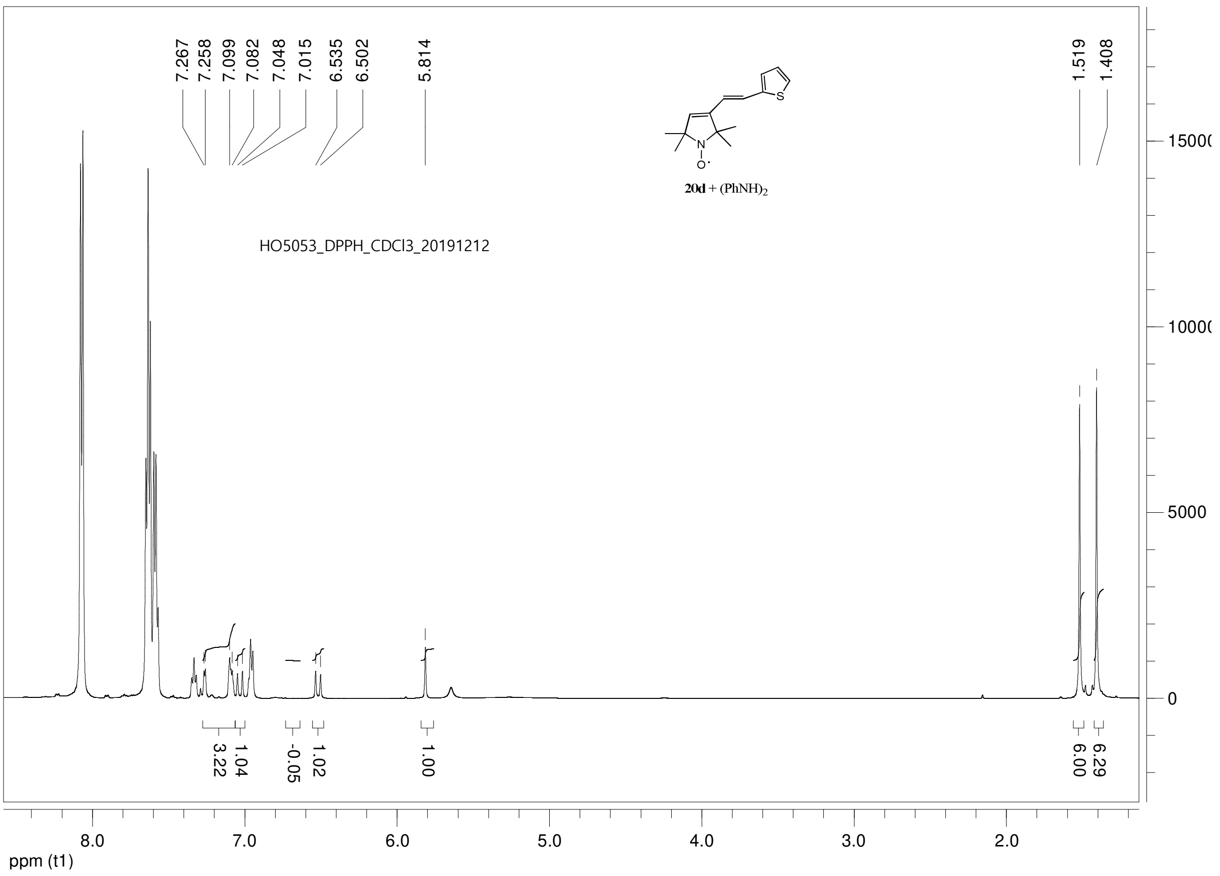
7.267
7.258
7.099
7.082
7.048
7.015
6.535
6.502
5.814
1.519
1.408
20d + (PhNH)2
HO5053_DPPH_CDCl3_20191212
3.22
1.04
-0.05
1.02
1.00
6.00
6.29
ppm (t1)

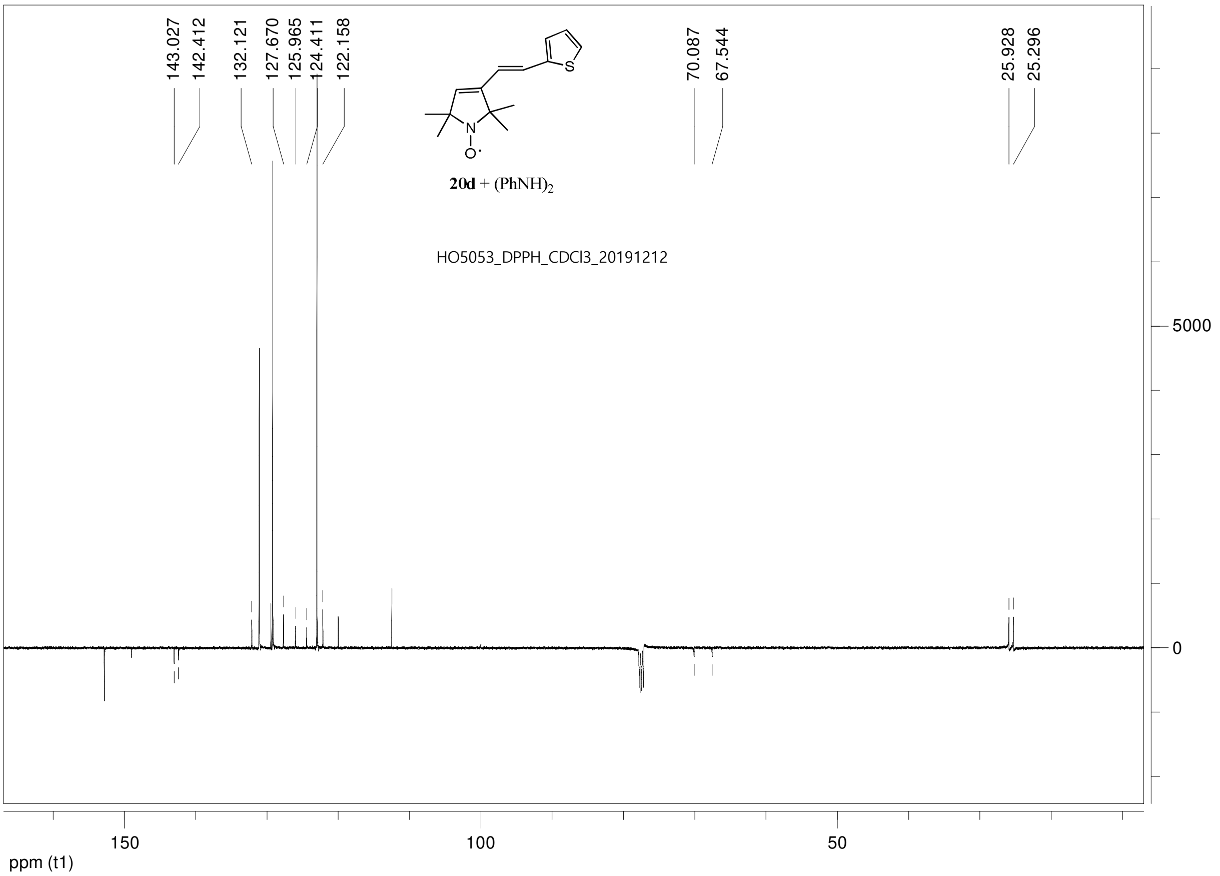
143.027
142.412
132.121
127.670
125.965
124.411
122.158
70.087
67.544
25.928
25.296
S
N
O
20d + (PhNH)$_2$
HO5053_DPPH_CDCl3_20191212
5000
0
150
100
50
ppm (t1)

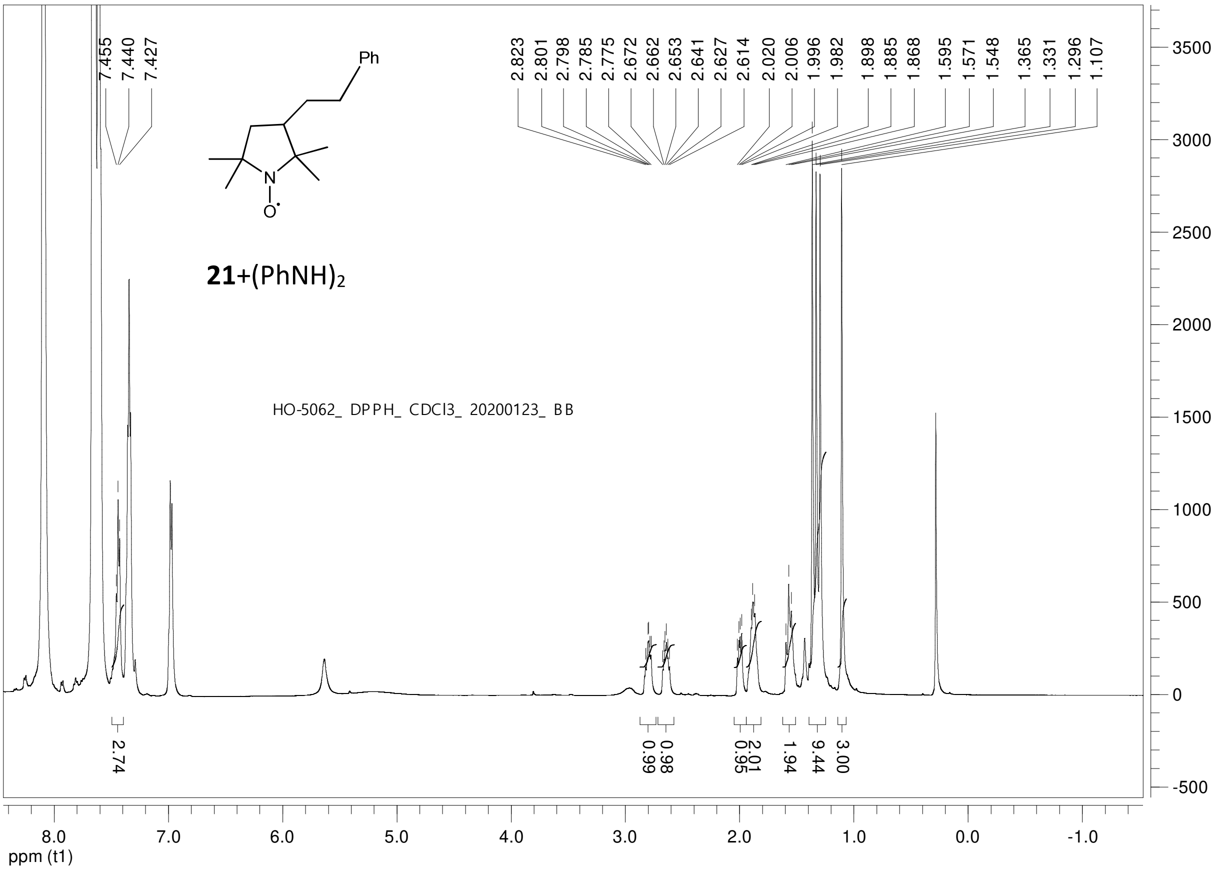
7.455
7.440
7.427
Ph
N
O
21+(PhNH)$_2$
2.823
2.801
2.798
2.785
2.775
2.672
2.662
2.653
2.641
2.627
2.614
2.020
2.006
1.996
1.982
1.898
1.885
1.868
1.595
1.571
1.548
1.365
1.331
1.296
1.107
HO-5062_ DPPH_ CDCl3_ 20200123_ BB
3500
3000
2500
2000
1500
1000
500
0
-500
2.74
0.99
0.98
0.95
2.01
1.94
9.44
3.00
8.0
7.0
6.0
5.0
4.0
3.0
2.0
1.0
0.0
-1.0
ppm (t1)

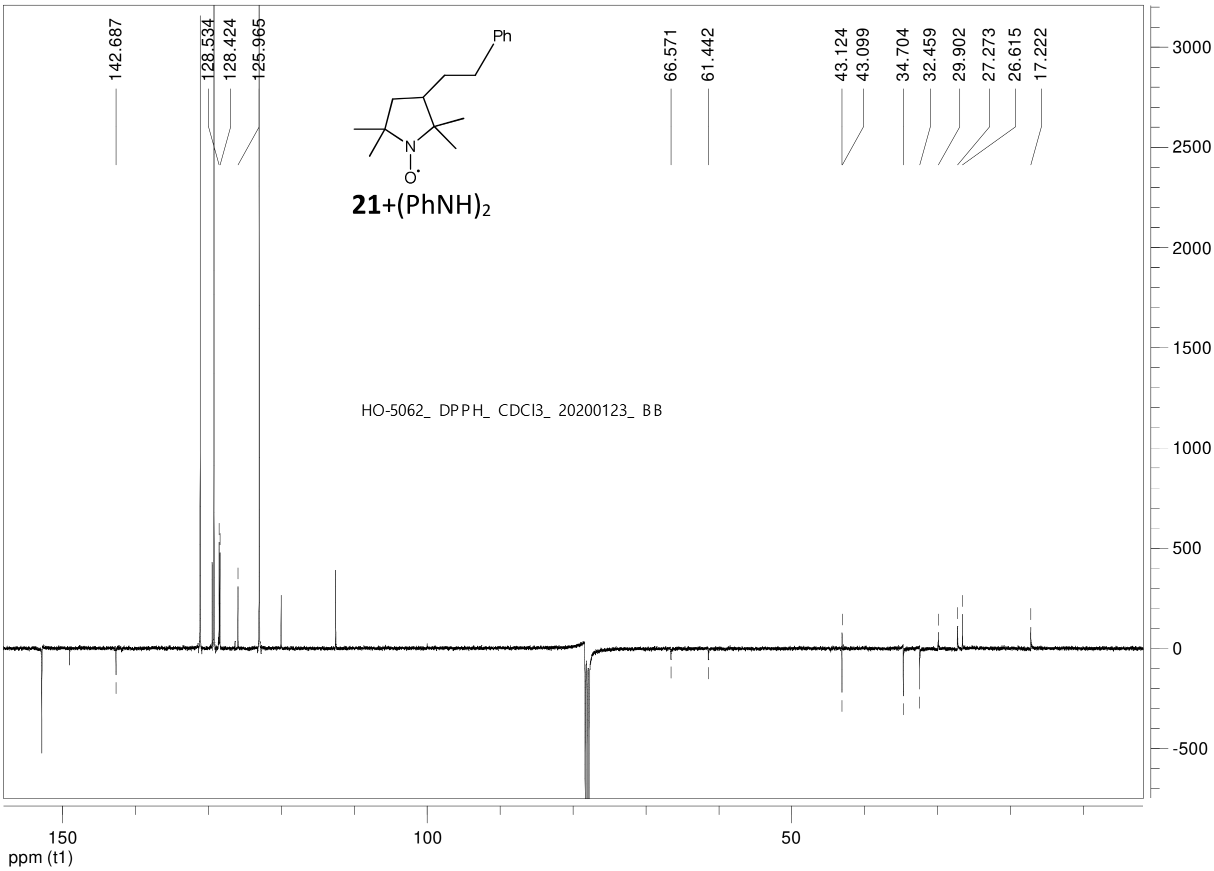
142.687
128.534
128.424
125.965
66.571
61.442
43.124
43.099
34.704
32.459
29.902
27.273
26.615
17.222
Ph
N
O
21+(PhNH)2
HO-5062_ DPPH_ CDCl3_ 20200123_ BB
3000
2500
2000
1500
1000
500
0
-500
150
100
50
ppm (t1)

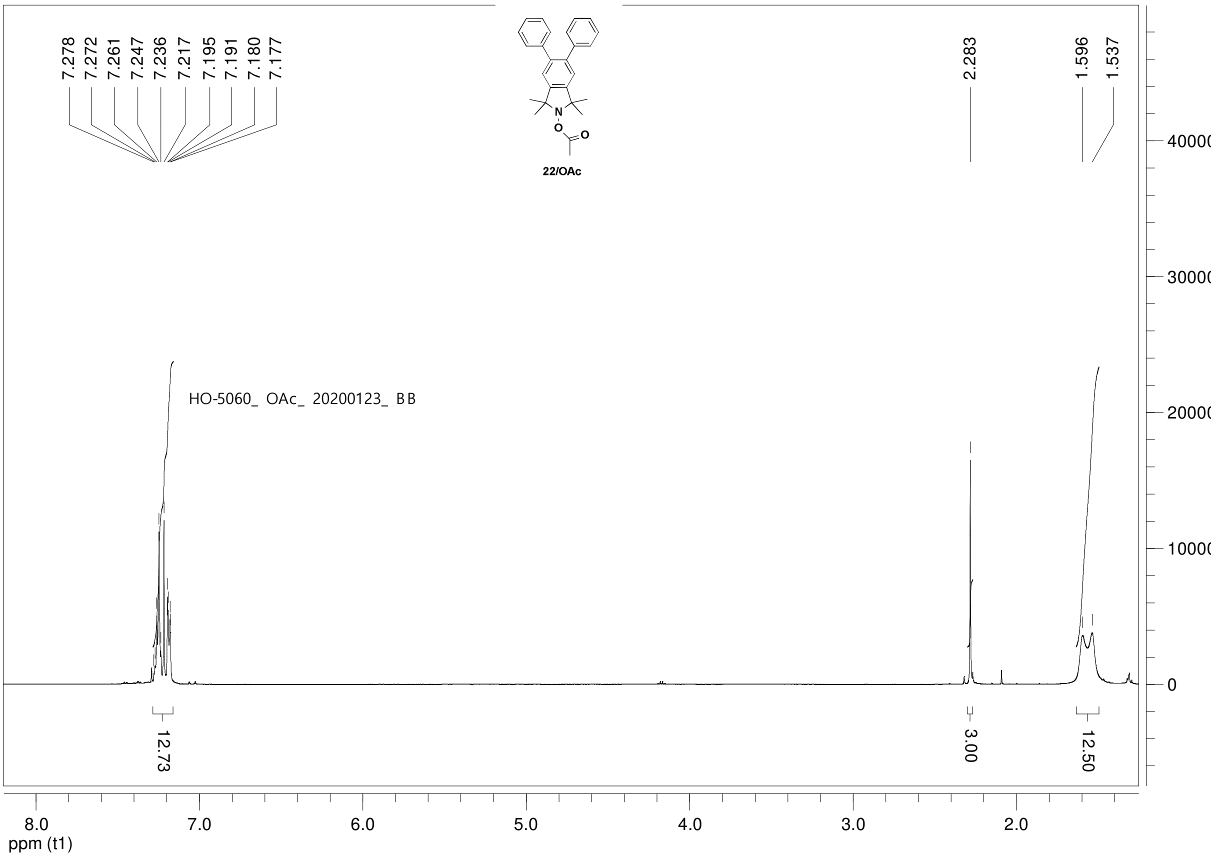
7.278
7.272
7.261
7.247
7.236
7.217
7.195
7.191
7.180
7.177
2.283
1.596
1.537
N
O
O
22/OAc
HO-5060_ OAc_ 20200123_ BB
12.73
3.00
12.50
8.0
7.0
6.0
5.0
4.0
3.0
2.0
ppm (t1)

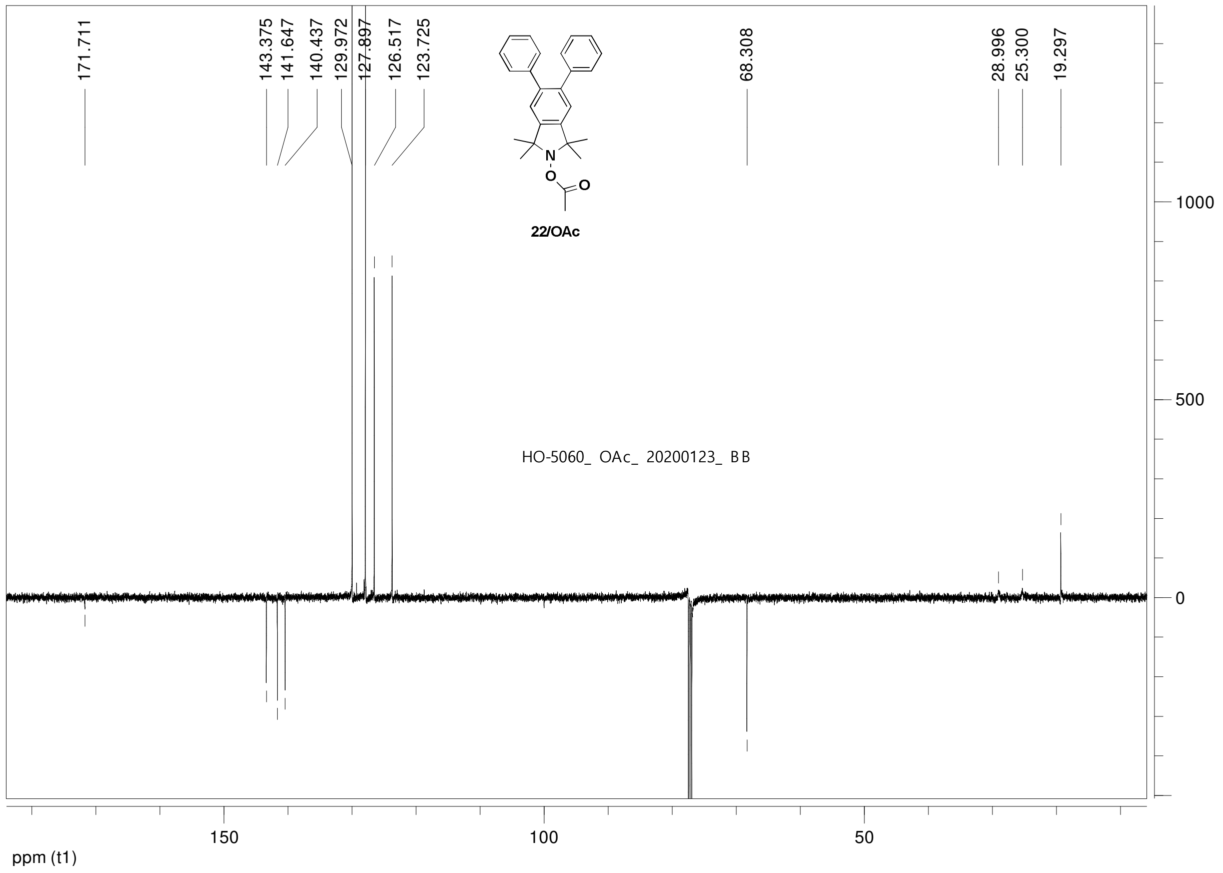
171.711
143.375
141.647
140.437
129.972
127.897
126.517
123.725
68.308
28.996
25.300
19.297
22/OAc
HO-5060_ OAc_ 20200123_ BB
1000
500
0
150
100
50
ppm (t1)

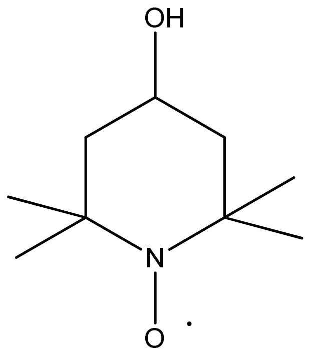

TEMPOL

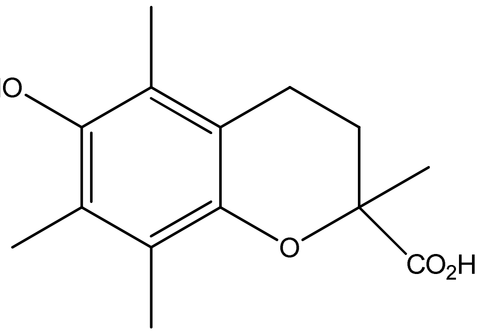

Trolox
